# Assembling programmable FRET-based photonic networks using designer DNA scaffolds

**DOI:** 10.1038/ncomms6615

**Published:** 2014-12-11

**Authors:** Susan Buckhout-White, Christopher M Spillmann, W. Russ Algar, Ani Khachatrian, Joseph S. Melinger, Ellen R. Goldman, Mario G. Ancona, Igor L. Medintz

**Affiliations:** 1 Center for Bio/Molecular Science and Engineering, Code 6900, U.S. Naval Research Laboratory, 4555 Overlook Avenue, South West, Washington DC, Washington 20375, USA; 2 College of Science, George Mason University, 4400 University Drive, Fairfax, Virginia 22030, USA; 3 Department of Chemistry, University of British Columbia, 2036 Main Mall, Vancouver, British Columbia V6T 1Z1, Canada; 4 Electronic Science and Technology Division, Code 6800, U.S. Naval Research Laboratory, 4555 Overlook Avenue, South West, Washington DC, Washington 20375, USA; 5 Sotera Defense Solutions, Inc., 7230 Lee DeForest Drive, Columbia, Maryland 21046, USA

## Abstract

DNA demonstrates a remarkable capacity for creating designer nanostructures and devices. A growing number of these structures utilize Förster resonance energy transfer (FRET) as part of the device's functionality, readout or characterization, and, as device sophistication increases so do the concomitant FRET requirements. Here we create multi-dye FRET cascades and assess how well DNA can marshal organic dyes into nanoantennae that focus excitonic energy. We evaluate 36 increasingly complex designs including linear, bifurcated, Holliday junction, 8-arm star and dendrimers involving up to five different dyes engaging in four-consecutive FRET steps, while systematically varying fluorophore spacing by Förster distance (*R*_0_). Decreasing *R*_0_ while augmenting cross-sectional collection area with multiple donors significantly increases terminal exciton delivery efficiency within dendrimers compared with the first linear constructs. Förster modelling confirms that best results are obtained when there are multiple interacting FRET pathways rather than independent channels by which excitons travel from initial donor(s) to final acceptor.

## Abstract (web summary)

DNA is a useful molecule with which to construct nanomaterials with controllable functionalities. Here, the authors fabricate photonic wires by appending dye molecules at set positions along DNA structures, and show how FRET performance can be tuned by modifying dye separation.

## Graphical abstract

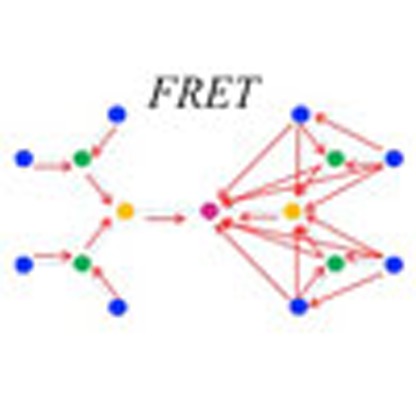

Structural DNA technology can create nanoassemblies having almost any conceivable multi-dimensional shape ranging from nanoscale world maps to gears and nanoflasks^1^,^2^,^3^,^4^,^5^. Capitalizing on this capability, DNA-based applications are being explored for biocomputing, sensing, electronic, biosynthetic, drug delivery and plasmonic devices^2^,^6^,^7^,^8^,^9^. Making these applications possible is not just Watson–Crick base pairing, but also that DNA can be custom synthesized and site-specifically modified with dyes, nanoparticles or a library of functional groups^10^,^11^ along with access to design tools (for example, cadnano, Nanoengineer and Uniquimer). Functionally, an increasing number of these structures incorporate multiple dyes and rely on Förster resonance energy transfer (FRET) occurring between them as part of the device's optical function/readout or to interrogate the assembly itself. Applications where DNA-based FRET have been demonstrated or exhibit strong potential include optical data storage^12^; molecular computing^6^,^8^,^13^; biosensing^14^,^15^; cryptography^16^; multicolour fluorescent probes^17^,^18^,^19^,^20^; photodynamic therapy^21^; nanoscale structural analysis^22^,^23^; signal transduction within nanoactuating devices^24^,^25^,^26^,^27^; chemistry^7^,^8^,^28^; light harvesting and charge conversion^29^,^30^; plasmonics^31^,^32^ and theranostics^2^,^21^,^33^,^34^. As DNA devices grow increasingly sophisticated so do the concomitant FRET requirements. Thus it is important to ascertain how complex FRET networks assemble on DNA scaffolds and what functional constraints will be imposed.

The most advanced use of DNA-organized fluorophores and FRET has been photonic wires, in which dyes are arranged with the goal of producing directed or sequential energy transfer^35^. A typical design involves 3–6 dyes linearly attached to DNA at separations typically less than their Förster distances^35^,^36^. Given the close spacing, predicted FRET efficiencies at each step should be >90%, but were typically found to be <40%, primarily because of structural heterogeneity, which led to the simultaneous presence of both highly and poorly efficient FRET subpopulations along with photobleaching issues. The addition of intercalator dyes within the DNA duplex by Albinsson^37^ improved energy migration, and Burley^38^ expanded this with tethered polyamides that controlled intercalator placement. We attached such wires to semiconductor quantum dots (QDs) and showed that end-to-end exciton transfer efficiency could be improved from ≤0.1% to ~10% by optimizing dye pairings/spacings and wire display valency around the central light-harvesting nanocrystal^39^,^40^. In more complex work, DNA origami-based rectangles have been used to demonstrate transfer pathway selection by placement of intermediary jumper dyes^41^. Directional transfer along three-way junctions, hexagons and tetrahedra have also been shown^17^,^20^,^38^. Perhaps the most elegant display originates from the study by Liu and co-workers^30^ who demonstrated a cyclic light-harvesting array organized by a seven-helix DNA bundle, allowing for estimated FRET efficiencies of ~90% based on donor quenching measurements.

Here, we utilize the power of DNA architecture to move beyond linear photonic wires to achieve more sophisticated DNA-arranged networks involving as many as 85 organic dye molecules engaged in programmable FRET cascades. We evaluate 36 antenna designs of increasing complexity by assembling >550 different DNA constructs. These include linear, bifurcated, Holliday junction, 8-arm star and 2:1, 3:1 or 4:1 branching dendrimer structures with either two-, four- or five-dye types, engaged in one-, three- or four-consecutive FRET steps, respectively, where inter-fluorophore spacings are systematically varied in increments of dye-pair Förster distances (*R*_0_). Optimizing dye placement by decreasing their spacing while increasing effective collection area with multiple initial donors in different geometries provides for >500-fold increase in terminal exciton delivery efficiency within the dendrimer structures in direct comparison to the first linear four-dye construct (one initial donor) placed at 1.5 × *R*_0_. Detailed Förster modelling reveals that crucial to the enhancement is the number of FRET pathways, where the best results are observed when there are multiple interacting rather than independent channels by which excitons travel from initial donor(s) to final acceptor. These studies also reveal certain non-idealities that appear to be explainable by formation inefficiency, inadequate fluorophore performance and a lack of control over dye orientation. Our results suggest possible design criteria by which increasingly sophisticated FRET-based DNA devices may be achievable.

## Results

### Fluorophores and DNA structures

In choosing the dyes, the possibility of them being photophysically perturbed/quenched by DNA is well-known although this phenomena still lacks a predictive understanding^42^,^43^. These effects are influenced by placement within or at the end of a sequence (that is, end capping) and by the nature of the dye itself (that is, hydrophobicity) along with its chemical attachment. Given our goal of assembling complex FRET networks displaying multi-dye cascades, it was not feasible to test every potential dye–DNA sequence interaction beforehand. We thus opted to rely predominantly on cyanine dyes as these have shown a higher overall resistance to DNA quenching than other dye families (even increasing in some cases)^42^,^44^,^45^,^46^,^47^,^48^,^49^, while anticipating some exceptions. As a precaution, dye-displaying DNA sequences were designed to minimize dye proximity to potentially quenching G and C bases wherever possible. The dyes used were chosen because they are commercially available and can be site-specifically attached to DNA, and most have been used previously in photonic wire studies, providing some archival background on their performance^30^,^35^,^37^,^39^,^40^. Dyes were inserted into DNA as succinimidyl ester modifications to terminal or internal amines or directly into the sequence itself using phosphoramidite chemistry based upon vendor availability. For terminal placement, succinimidyl ester dyes were used to modify 3′ or 5′ amines displayed at the end of a C6 or C7 alkane chain within an an amino linker. For phosphoramidites, terminal placement was as a directly inserted base mimic and for internal placement an unpaired A base was inserted in the complementary strand opposite the dye to minimize hybridization issues. Phosphoramidite insertion tends to constrain dye movement due to its two-point 5′–3′ attachment when internal and the short length of its linkers while the other dyes have more freedom of movement due to their single-point alkane chain linker attachments. This approach provides sufficient spacing such that for the 0.5 × *R*_0_ constructs the dyes have enough separation so that we did not observe adverse fluorophore–fluorophore interactions such as heterodimer formation (data not shown). See Supplementary Fig. 1 for chemical structures of the dyes and linkers and Supplementary Note 1 for DNA sequences/modifications.

**Figure 1:**
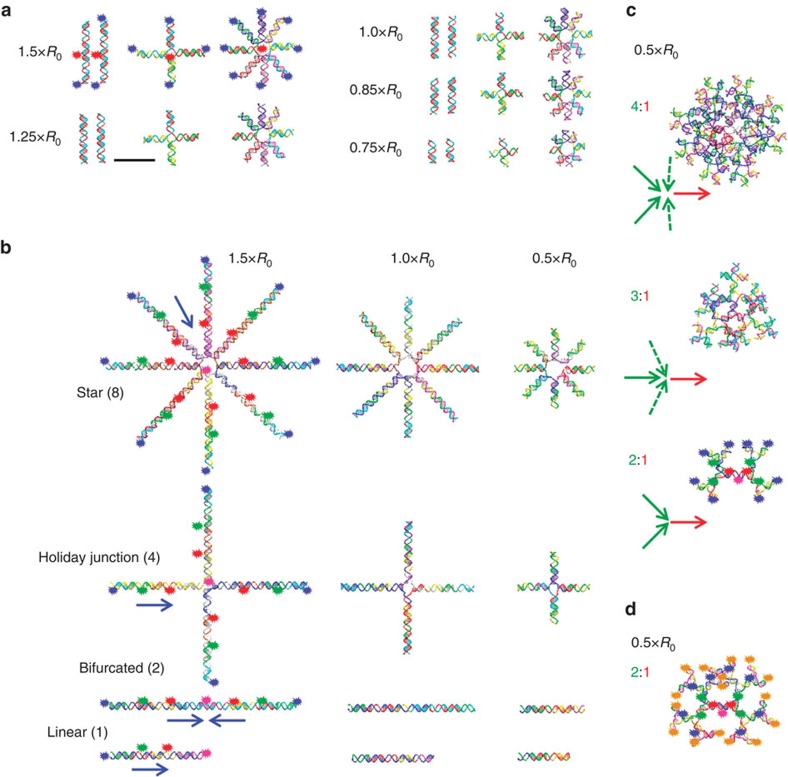
DNA structures. (**a**) Cy3_*n*_→Cy5 two-dye single Förster resonance energy transfer (FRET) step system consisting of *n* Cy3 donors (blue) paired to a single central Cy5 acceptor (red). The *n*=Cy3/Cy5 ratio is incrementally increased using linear (*n*=1), bifurcated (*n*=2), Holliday junction (*n*=4) and star (*n*=8) structures. Donor–acceptor spacings were also varied for each structure in increments of the Förster distance or *R*_0_~54 Å (0.75 × , 0.87 × , 1.0 × , 1.25 × and 1.5 × *R*_0_). The 1.5 × *R*_0_ structures show approximate dye locations relative to the DNA. (**b**) [Cy3→Cy3.5→Cy5]_*n*_→Cy5.5 four-dye, three FRET step system with sequential donor–acceptor arrangements of Cy3 (blue), Cy3.5 (green), Cy5 (red) and Cy5.5 (pink) in photonic wire configurations. The number of [Cy3→Cy3.5→Cy5]_*n*_ wires leading into each terminal Cy5.5 dye increases similarly from one to eight using linear, bifurcated, Holliday junction and eight-arm star constructs. The blue arrows show the directionality of the FRET cascade(s) along each wire in each structure as they converge on the terminal Cy5.5 acceptor. Donor–acceptor spacing varied as 0.5 × , 1.0 × and 1.5 × *R*_0_. The 1.5 × *R*_0_ schematic shows the approximate dye positions. (**c**) Branched 0.5 × *R*_0_ dendrimer-based FRET systems utilizing Cy3, Cy3.5, Cy5 and Cy5.5 dyes in configurations were each dye preceding the central-terminal Cy5.5 has two, three, or four donors. Donor–acceptor spacings for the dendrimers were fixed at 0.5 × *R*_0_, and the 2:1 structure shows approximate dye locations. (**d**) Dendrimer-based five-dye FRET system utilizing AF488 (orange), Cy3 (blue), Cy3.5 (green), Cy5 (red) and Cy5.5 (pink) dyes in a configuration, where each dye preceding the central-terminal Cy5.5 has two donors. An alternate version was assembled with AF647 replacing Cy5. Arrows schematically highlight the general donor (green) to acceptor (red) architecture. The following descriptions for the structures are used interchangeably throughout the text: linear or 1-way or unidirectional; bifurcated or 2-way or bidirectional; Holliday or 4-way or Holliday junction; star or 8-arm or 8-way or 8-arm star or 8-way junction; 2:1 or 2-1 dendrimer: 3:1 or 3-1 dendrimer and 4:1 or 4-1 dendrimer. Photonic wire, Holliday junction and eight-arm stars are sometimes referred to generically as stars. Black line in **a** is a 10 nm size reference and all DNA structures are scaled accordingly.

With downstream utility in mind, our design approach was kept simple: all structures should self-assemble by one-pot hybridization without requiring enzymatic ligation, and to avoid potential degradation in yield, structures would be directly assessed without post-assembly purification. The three different structural systems studied here (Fig. 1) are based on *de novo* structures and adaptations from previous reports; see Supplementary Tables 1–37 for sequences and Supplementary Methods. Structures were designed so that approximate dye spacing for each FRET donor-acceptor pair in a given construct was a fixed fraction of the Förster distance or *R*_0_ (see Methods for calculation), characterizing the range of FRET interactions in the point-dipole approximation^50^.

The two-dye single-FRET step system, designated Cy3_*n*_→Cy5 (Fig. 1a), consisted of *n* Cy3 donors surrounding a single-central Cy5 acceptor with *n=*1,2,4,8 and termed linear, bifurcated, Holliday junction and star or eight-arm star, respectively. Linear/bifurcated structures were organized by linear DNA templates while Holliday junction/eight-arm star designs were adapted from ref. 51. Donor–acceptor spacings in each structure varied as fractions of the Cy3–Cy5 *R*_0_ (~54 Å) at nominal values of 0.75 × , 0.85 × , 1.0 × , 1.25 × and 1.5 × *R*_0_ with predicted FRET efficiencies ranging from ~80% to 5%, respectively. The second system (Fig. 1b) involved photonic wires with up to four dyes and three FRET steps in the configuration [Cy3→Cy3.5→Cy5]_*n*_→Cy5.5 with *n* three-dye photonic wires feeding the central Cy5.5 dye and again varying in ratio from 1 to 8 in linear, bifurcated, Holliday junction and eight-arm star designs, respectively. Nominal donor–acceptor spacings were varied here as 0.5 × , 1.0 × and 1.5 × *R*_0_ for each given dye pair (*R*_0_ values, Table 1) corresponding to predicted energy transfer efficiencies of ~95%, 50% and 5%, respectively^50^. The 0.5 × *R*_0_ designs aim to maximize efficiency, while still avoiding undesirable interactions (that is, dye homo/heterodimers). Given that these assemblies were designed to sample a linearly increasing range of either initial discrete Cy3_*n*_ donor or three-dye [Cy3→Cy3.5→Cy5]_*n*_ photonic wire donors, the latter 1.0 × and 1.5 × *R*_0_ spacings were included to specifically evaluate if multiple donors at longer spacings could augment energy transfer by increasing the probability that FRET occurs.

The third FRET system was dendrimers (Fig. 1c,d), with most designs utilizing the same four dyes but arranged as [[Cy3_*n*_→Cy3.5]_*n*_→Cy5]_*n*_→Cy5.5 with *n*=2,3,4 (that is, each dye preceding the central Cy5.5 has two to four donors). Due to the structural complexity (for example, when *n*=4 there are a total of 85 dyes), only dye spacings of 0.5 × *R*_0_ were studied. One five-dye dendrimer design (Fig. 1d) added an initial Alexa Fluor 488 dye (AF488) in a 2:1 [[AF488_2_→Cy3]_2_→Cy3.5]_2_→Cy5/AF647]_2_→Cy5.5 configuration with either Cy5/Alexa Fluor 647 (AF647) in the fourth penultimate position. In addition to the two-, four- and five-dye photonic wire/dendrimer constructs with their full complement of dyes, we analyzed all partially functionalized forms, with dye display evolving from initial donor to fully decorated structure. Structures with one- or two-dyes missing were also examined to estimate longer range FRET contributions. Unlabelled DNA was always substituted for labelled DNA to retain the same underlying structure. Although depicted in a flat two-dimensional (2D) perspective (Fig. 1), these structures are 3D with variability due to flexibility at the junctions between the relatively stiff DNA duplexes, and the analysis specifically incorporates this conformational space.

**Figure 2:**
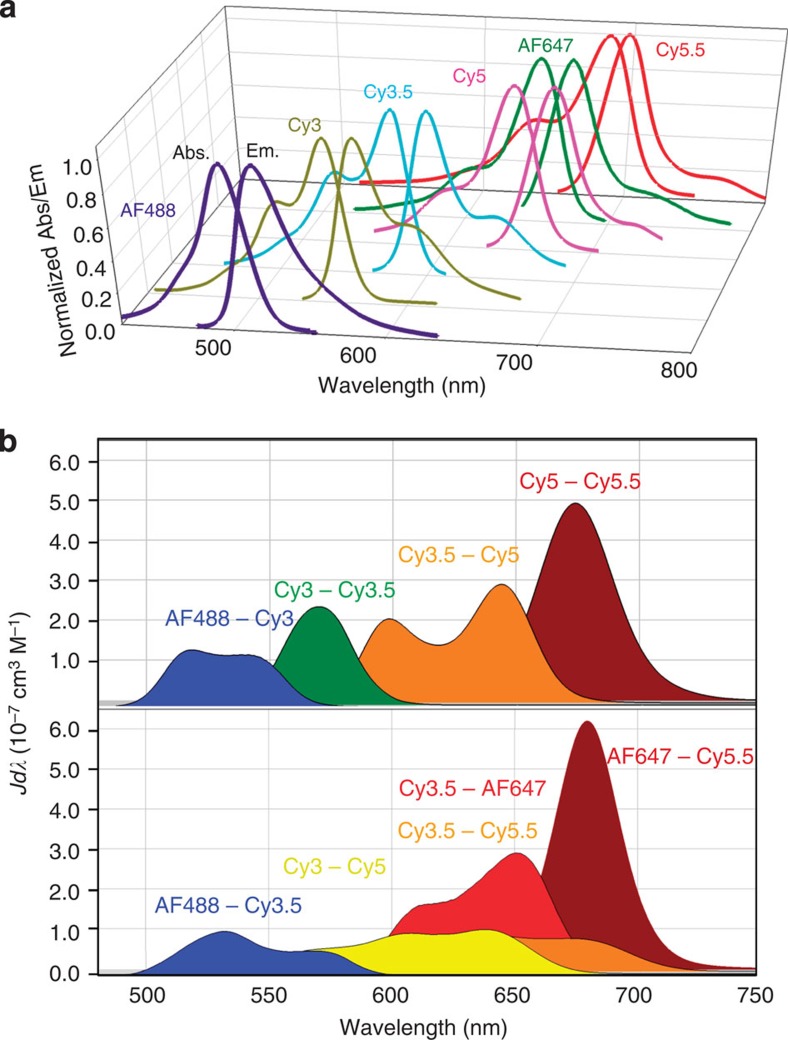
Fluorophore photophysical properties. (**a**) Normalized absorption/emission spectra for each of the dyes used. (**b**) Plots of the integrand of the *J* integral as a function of wavelength for the indicated donor–acceptor combinations.

Absorption/emission spectra for the dyes (Fig. 2a) highlight the potential of Cy3 to be excited at ~515–550 nm, and to transfer excitonic energy in one step to Cy5 (*λ*_max em._ 670 nm) or in a three-step FRET cascade across a ~180 nm portion of the spectrum to a terminal Cy5.5 (*λ*_max em._ 694 nm). Alternatively, AF488 can be excited at 460–490 nm and transfer energy across ~250 nm in a four-step cascade providing for Cy5.5 sensitization. Relevant dye photophysical parameters allowed calculation of spectral overlap integrals (*J*) and *R*_0_ values for each donor–acceptor pair (Table 1). *R*_0_ values varied between ~40–70 Å while *J* varied from 8.9 × 10^−14^ to 2.2 × 10^−12^ cm^3^ M^−1^. Plots of the *J* integrand versus wavelength for pertinent donor–acceptor pairs reinforce the concept of transferring exciton energy sequentially over a ~250 nm portion of the spectrum using multiple ET steps (Fig. 2b). The sequentially arrayed nature of the system suggests that longer range FRET, that is, skipping over an intermediary dye, should not be significant.

### Cy3_*n*_→Cy5 single-FRET step systems

**Figure 3:**
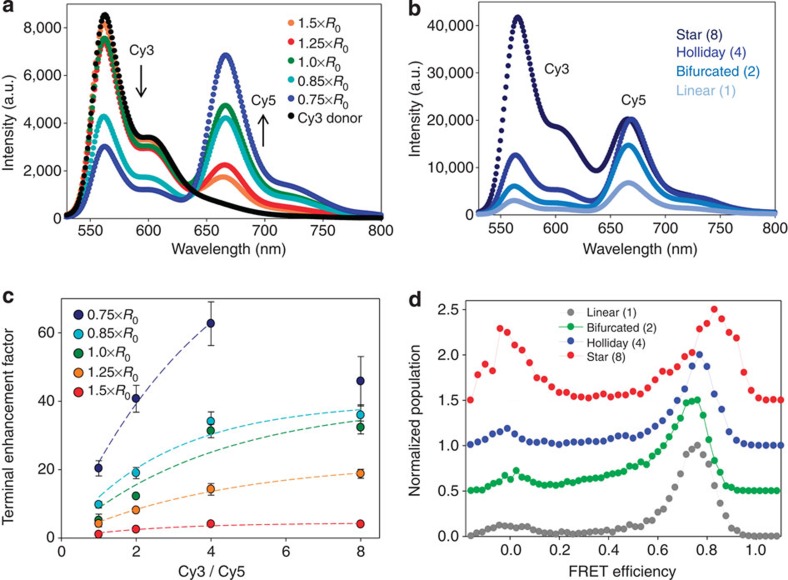
Two-dye single-FRET step systems. (**a**) Representative spectra excited at 515 nm, showing the effect of altering Cy3–Cy5 donor–acceptor spacing in the linear system as a function of *R*_0_ (~54 Å). The direct Cy3-only emission in each was used to normalize the data. (**b**) Representative spectra from linear, bifurcated, Holliday junction and eight-arm star Cy3–Cy5 constructs where donor–acceptor spacing was maintained at ~0.75 × *R*_0_. Data were normalized to the Cy5 alone emission. (**c**) Plot of the Cy5 terminal enhancement factor (TEF) as a function of the number of Cy3 donors per Cy5 acceptor for each of the donor–acceptor spacings as compared with the initial 1.5 × *R*_0_ Cy3_1_→Cy5 system. Data are averaged from at least three independently assembled constructs and are plotted with the s.d. Trend lines are added to aid the eye. (**d**) Representative single-pair or spFRET histograms for all 0.75 × *R*_0_ constructs, see Supplementary Note 4. The maximum number of FRET events for each histogram has been normalized to unity. The green, blue and red curves are stacked upward for comparative presentation. Note the growth of the zero-FRET efficiency population (left) in the star structure and the shift to higher efficiency (right) from the linear to the star structure.

This system evaluated sensitization of a single acceptor as a function of increasing donor number and dye spacing. The DNA scaffold (Fig. 1a) arrayed one, two, four or eight Cy3 donors around a central Cy5 acceptor at separation distances ranging from 0.75 × to 1.5 × *R*_0_ (~40–81 Å). All samples were prepared in 2.5 × PBS without Mg^2+^ to avoid dye quenching issues at high millimolar ionic concentrations^52^,^53^, and emission spectra were collected using 515 nm excitation (Methods). Spectra were collected from the Cy3_1_→Cy5 system as dye spacing was varied in comparison to Cy3 alone (Fig. 3a). Here, Cy3 donor emission decreases while Cy5 acceptor sensitization increases with decreasing separation distance (Supplementary Table 38). Normalized spectra for the closest 0.75 × *R*_0_ assemblies as Cy3 to Cy5 ratio increases, where direct Cy3 emission also increases concomitantly with ratio/valence, reveals that Cy5 sensitization saturates at *n*=4 (Fig. 3b). Representative raw data plots for the two-dye constructs are presented in Supplementary Figs 2–6. Normalized spectra at all other spacings are presented in Supplementary Fig. 7.

To quantitatively compare FRET network performances, we assess the energy harvesting/sensitizing characteristics using metrics of terminal enhancement factor (TEF), antenna effect (AE) and exciton end-to-end efficiency (*E*); definitions in Methods, Supplementary Note 2. TEF and AE are relative measures of performance giving the enhancement of final acceptor output over either a reference system (TEF) or in comparison to direct excitation of terminal dye (AE), while *E* estimates efficiency of exciton delivery. TEF utilizes a single upstream excitation and monitors output from the final acceptor with the 1.5 × *R*_0_ linear constructs typically used as reference. Since the latter has the fewest initial donors and largest dye separations, it manifests weakest sensitization and so TEF will give large values. Its merits are that it thus amplifies improvements while also providing perspective on final output performance across all assemblies in a given system as donor number(s), dye spacing and geometries vary. AE looks at relative efficiency increases between a higher energy excitation/sensitization of multiple donors and direct excitation of a given acceptor, providing a measure of efficiency within the same structure. Plotting TEF as a function of donor number for each donor–acceptor spacing in the Cy3_*n*_→Cy5 system shows that acceptor output can be magnified >60 times by decreasing inter-fluorophore separation to 0.75 × *R*_0_ and increasing donor number to four (Fig. 3c). The 0.75 × *R*_0_ Holliday junction manifests the best AE at ~3 while the 0.75 × *R*_0_ bifurcated system achieves an *E* of ≥50% (Table 2, Supplementary Fig. 8). Excepting the 0.75 × *R*_0_ structures, AE generally increases with Cy3 donor number, while *E* values are relatively constant up to a ratio of 4 and then decrease at 8.

As donor number increases we expect TEF and AE to rise^50^,^54^,^55^, so their drop when going from 4 to 8 in the 0.75 × *R*_0_ structure appears anomalous (Fig. 3c, Table 2). In terms of defective hybridization, we estimate formation efficiencies for the eight-arm star of ~10%/~40% for complete/partial structures from gel electrophoresis (Supplementary Fig. 9, Supplementary Table 39, Supplementary Note 3), thus one still expects Cy5 to have sufficient sensitizing-Cy3 donors to at least equal the Holliday structure. An alternative explanation comes from noting that this star has a large ring-like opening at its center of ~30 Å (Fig. 1b), which, in conjunction with its phosphoramidite attachment, could force Cy5 into an asymmetric position and thereby lower the average FRET efficiency^51^. This effect should become less important as dye spacings increase, which is seen in the data. Further evidence for this comes from single-pair FRET (spFRET) measurements of the 0.75 × *R*_0_ Cy3_*n*_→Cy5 series (Fig. 3d, Supplementary Note 4). Most interesting are the eight-arm star data, where a bimodal histogram reveals that total efficiency is an average of high FRET and low FRET pathways that correspond to excitation of Cy3 dyes located near or far from the Cy5 in the eight-arm structure. Thus, it seems likely that structural non-ideality is responsible for much of the performance drop-off seen when going from the four-arm structure to the eight-arm (Fig. 3c, Table 2); a similar but smaller effect is expected to operate in the Holliday junctions.

spFRET data also provided insight into underlying structural heterogeneity. If the DNA structures consisted of multiple subpopulations assuming different shapes based upon incomplete hybridization, incomplete assembly, slippage, structural breathing and/or different conformations, one would expect the multiple donor–acceptor distances to manifest as a broader near-continuous peak due to the cumulative underlying range of smeared FRET efficiencies^56^. In contrast, FRET efficiencies manifest as narrow distributions that shift as expected indicating that the structures are fairly homogeneous (Fig. 3d).

### [Cy3→Cy3.5→Cy5]_*n*_→Cy5.5 photonic constructs

**Figure 4:**
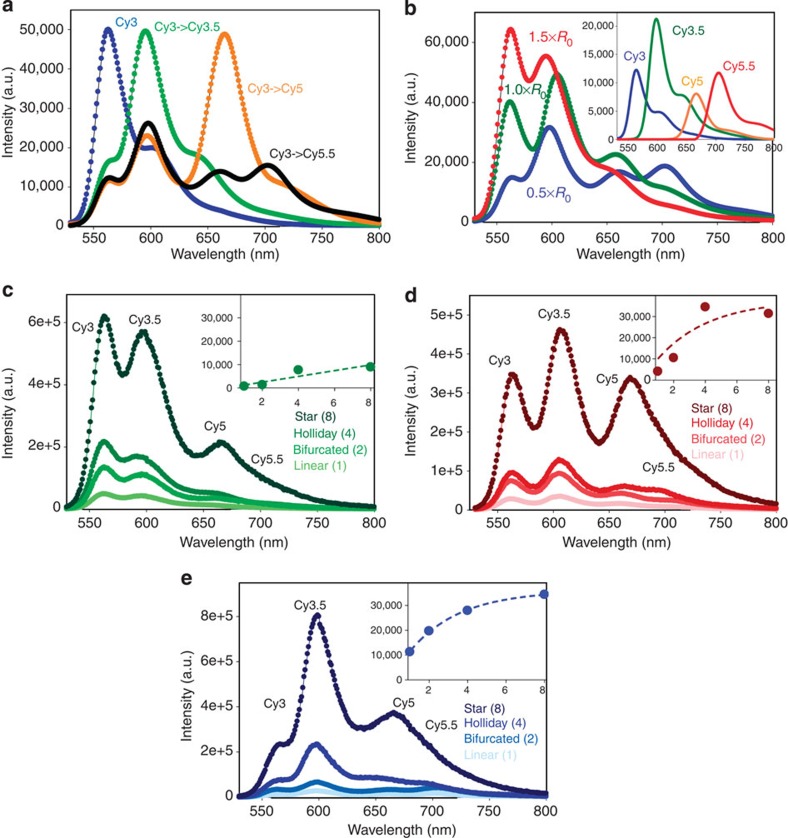
Four-dye photonic wire systems. (**a**) Representative data showing the spectral evolution of the 0.5 × *R*_0_ linear [Cy3→Cy3.5→Cy5]_1_→Cy5.5 system as consecutive acceptor dyes are added to the initial Cy3 donor. (**b**) Representative comparative spectral data for the 0.5 × , 1.0 × and 1.5 × *R*_0_ linear systems. Data were normalized to the direct Cy5.5 emission at the same excitation. Inset, decomposed individual component spectra for the 0.5 × *R*_0_ linear system. Representative spectra showing the FRET evolution of the (**c**) 1.5 × *R*_0_, (**d**) 1.0 × *R*_0_ and (**e**) 0.5 × *R*_0_ systems as the number of arms for each was increased from one to eight. The insets in panels **c**–**e** plots the increase in Cy5.5 sensitized emission as a function of the number of arms, all on the same scale. For comparison these data are all normalized to the direct Cy5.5 excitation component.

This system is the simplest generalization of the two-dye system into an antenna with more dyes, a larger collection area and potentially better performance. The spectral evolution of the initial 0.5 × *R*_0_ linear system as consecutive acceptor dyes are added to the initial Cy3 donor shows clear loss of donor emission and concurrent sensitization with sequential acceptor addition (Fig. 4a). Comparing spectra for the 1.5 × , 1.0 × and 0.5 × *R*_0_ linear constructs confirms that closer dye spacings do indeed improve FRET efficiency as expected (Fig. 4b, Supplementary Figs 10–21). Although the 1.5 × *R*_0_ system does show some increase in *E* for the star structures, only the 1.0 × *R*_0_ Holliday/star and 0.5 × *R*_0_ constructs reveal substantial Cy5.5 sensitization (Fig. 4c–e). See Supplementary Tables 40–52 for individual donor loss, acceptor sensitization and end-to-end efficiency and the scaled photoluminescent intensity of each fluorophore. The increased effect of wire display valency (from 1–8 for structures in Fig. 1b) along with decreasing respective inter-dye spacings from 1.5 × to 1.0 × and then 0.5 × *R*_0_ provide for increased *E* at each increment while avoiding direct dye–dye interactions. These plots confirm that the DNA appears to accurately control spacing of the four dyes with the net effect propagating easily through the three-step FRET transfer. Formation efficiencies (Supplementary Fig. 9, Supplementary Table 53) ranged from 20–90% for fully formed structures based on gel electrophoresis and fast protein liquid chromatography analysis (FPLC).

Subtracting the directly excited Cy3.5 component from the 0.5 × *R*_0_ bifurcated structure’s spectrum (Fig. 4e) finds no residual Cy3.5 emission, indicating it’s executing near-unity energy transfer. The 0.5 × *R*_0_ bifurcated system also shows the best AE at ~3. AE and *E* metrics for these data (Table 3) also reflect the better performance of the 0.5 × *R*_0_ construct. Most interesting is that efficiency *E* is roughly the same irrespective of the number of arms, suggesting that the arms act as independent photonic wires without appreciable exciton transfers between them.

### Dendrimers with increasing branching ratio

**Figure 5:**
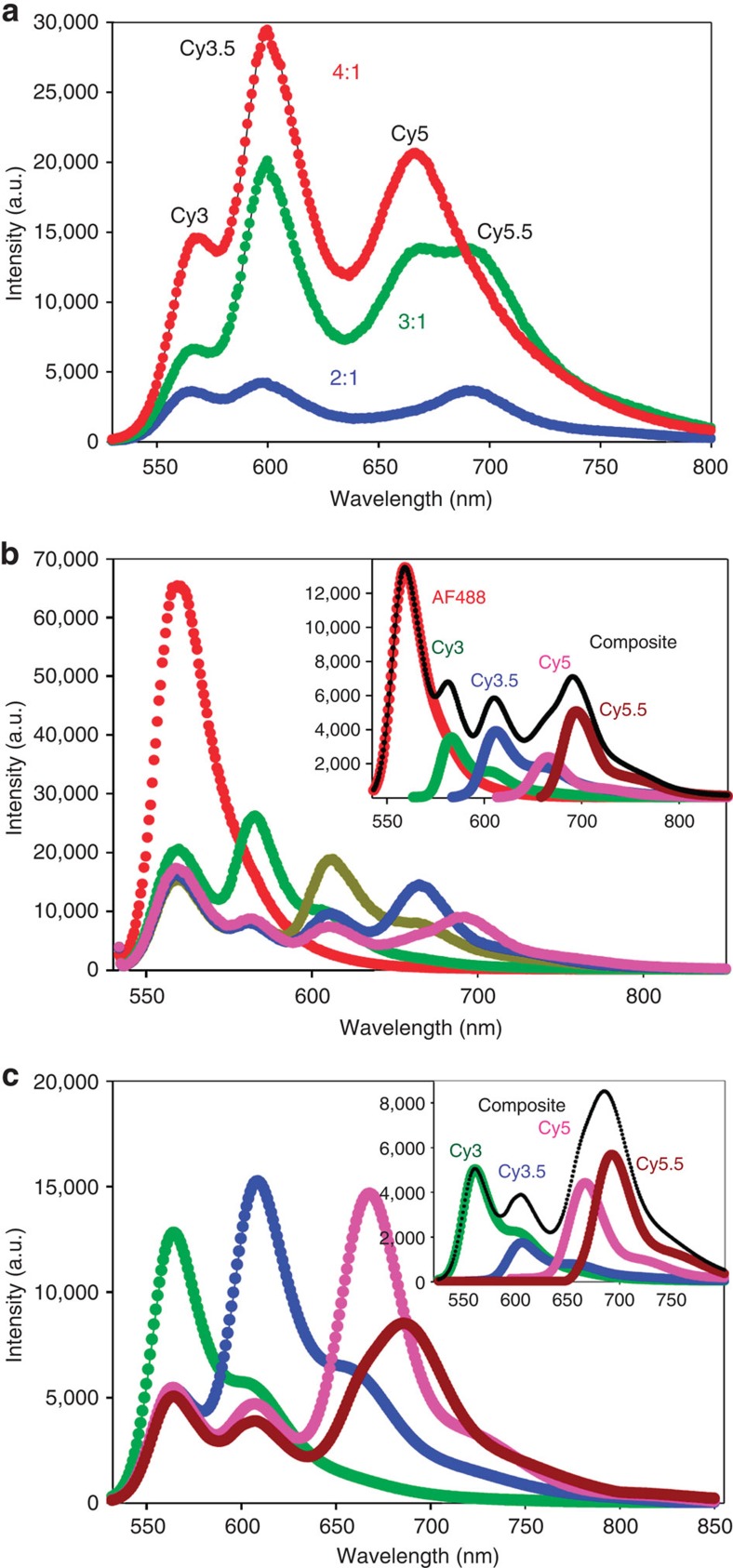
Four-dye and five-dye-branched dendrimer systems. (**a**) Representative comparative spectral data for the fully assembled 2:1, 3:1, and 4:1 0.5 × *R*_0_ dendrimer structures. Dye stoichiometries for dendrimer structures: 2:1=Cy3_8_→Cy3.5_4_→Cy5_2_→Cy5.5_1_; 3:1=Cy3_27_→Cy3.5_3_→Cy5_3_→Cy5.5_1_ and 4:1=Cy3_64_→Cy3.5_16_→Cy5_4_→Cy5.5_1_. Note: all data in **a**–**c** were normalized to the direct Cy5.5 emission. (**b**) Representative spectral data following the evolution of the five-dye 2:1 0.5 × *R*_0_ dendrimer AF488_16_→Cy3_8_→Cy3.5_4_→Cy5_2_→Cy5.5_1_ system. Constructs with dyes present and their corresponding colours are AF488 only (red), AF488→Cy3 (green), AF488→Cy5 (olive), AF488→Cy3 (navy) and AF488→Cy5.5 (pink). (**c**) Representative four-dye 0.5 × *R*_0_ Cy3_8_→Cy3.5_4_→Cy5_2_→Cy5.5_1_ 2:1 dendrimer systems. Constructs with dyes present and their corresponding colours are Cy3 only (green), Cy3→Cy3.5 (navy), Cy3→Cy5 (pink) and Cy3→Cy5.5 (brown). The construct in **c** has the Cy3.5 ester dye replaced by a Cy3.5 phosphoramidite within the oligos. Inset in **b**,**c** shows the spectra of fully formed structure along with each of the contributing component dyes. Constructs with Cy3 as the initial dye were excited at 515 nm, while those with AF488 were excited at 465 nm.

The dendrimeric systems have larger numbers of chromophores arranged with higher packing densities. Designs are based on Luo’s structures^57^, with each dye preceding the Cy5.5 sensitized by 2, 3 or 4 donors, and with dye spacings maintained at ~0.5 × *R*_0_ for high efficiency. Reflecting their complexity, within the [[Cy3_*n*_→Cy3.5]_*n*_→Cy5]_*n*_→Cy5.5 designs, the ratio of Cy3:Cy5.5 grows exponentially with branching ratio from 8 (2^3^) to 27 (3^3^) to 64 (4^3^) (Fig. 1c), and initial donor absorption cross-section is likewise amplified by a factor of ~6, 21 and 50 over that of the single terminal Cy5.5 acceptor (Table 3). Normalized spectra collected from the four-dye dendrimer structures show that spectral profiles tend towards a bimodal appearance (Fig. 5a, see Supplementary Figs 22–24 for raw data profiles). Sensitized Cy5.5 (>700 nm) is most prominent in the 3:1 assembly with AE ~4 and the highest *E* at 23% (Table 3, Supplementary Tables 54–56). We estimate formation efficiencies of 70, 20 and 10% for the 2:1, 3:1 and 4:1 structures, respectively (Supplementary Fig. 25, Supplementary Table 53), and partially attribute the fall-off at higher branching number to increased design complexity.

We extended the 2:1 dendrimer design by adding an initial AF488 donor to create a [[AF488_2_→Cy3]_2_→Cy3.5]_2_→Cy5]_2_→Cy5.5 construct with 0.5 × *R*_0_ dye spacing. Spectra collected as they evolve to incorporate all five dyes (Fig. 5b, Supplementary Fig. 26) show significant emission from the terminal Cy5.5 with a formation efficiency of ~60% (Supplementary Table 53). Despite the added FRET step, *E* and AE values are comparable to the previous four-dye/three-FRET step dendrimers at 19% and 1.8%, respectively (Supplementary Table 57). Modelling studies discussed below suggested non-ideal Cy5 performance as being potentially responsible for performance issues. This motivated an alternate construct where AF647 was substituted for Cy5 in the penultimate step (Supplementary Figs 27 and 28). Subsequent *E* and AE values remained essentially unchanged (16% and 1.4, respectively), suggesting that the problem is associated with DNA assembly rather than dye performance (Supplementary Table 58). Lastly, we recapitulated the [[Cy3_2_→Cy3.5]_2_→Cy5]_2_→Cy5.5 four-dye 2:1 dendrimer structure with the Cy3.5 ester dye (flexible tether) replaced by one inserted into the DNA as a localized phosphoramidite (Fig. 5c, Supplementary Fig. 29). Here *E* and AE values appear improved at 28% and 3.5, respectively (Supplementary Table 59). The increase in FRET is partly due to the higher fluorescence quantum yield (~2 × ) of the Cy3.5 dye, which may originate from the more rigid double phosphoramidite attachment in the context of this DNA sequence (*vide infra*).

### Comparison of energy migration

**Figure 6:**
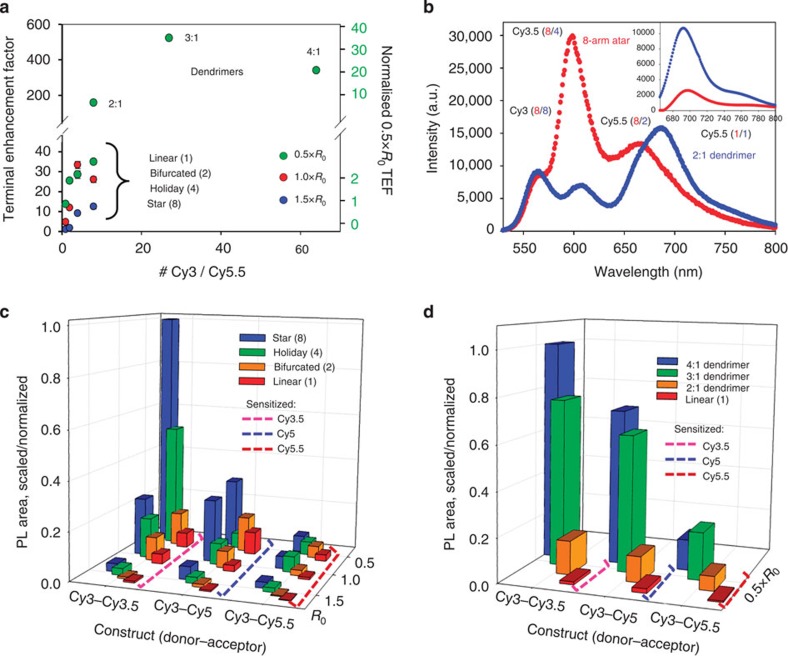
Comparative energy transfer in the photonic wire and dendrimer systems. (**a**) Plot of the Cy5.5 terminal enhancement factor (TEF) for the [Cy3→Cy3.5→Cy5]_*n*_→Cy5.5 photonic wire and the 2:1, 3:1 and 4:1 0.5 × *R*_0_ dendrimer structures as compared with the initial 1.5 × *R*_0_ linear system (left axis). Note the break in vertical scale. The right scale plots TEF for only the 0.5 × *R*_0_ structures as compared with the 0.5 × *R*_0_ linear four-dye system. Data are averaged from at least three independently assembled constructs and are plotted with the s.d. (**b**) Comparison of the normalized emission profiles for the 0.5 × *R*_0_ 2:1 dendrimer and eight-arm photonic wire star structures. Dye ratios corresponding to each position in each structure are indicated with red or blue numbers in parenthesis. Inset: the decomposed Cy5.5 sensitization for the 2:1 dendrimer (blue) is much larger than for the eight-arm star (red). (**c**) Comparative plot of the sensitized components at each step for the [Cy3→Cy3.5→Cy5]_*n*_→Cy5.5 photonic wire system. Dye emissions are scaled and normalized to the highest intensity contribution, which is the Cy3.5 sensitized emission in the eight-arm star structure. (**d**) Comparative plot of the sensitized components at each step for the 0.5 × *R*_0_ 2:1, 3:1 and 4:1 branching dendrimer systems, respectively. Dye emissions are scaled and normalized to the highest intensity contribution, which is the Cy3.5 sensitized emission in the 4:1 structure. S.d. for **c**,**d** are <5%.

Direct comparison of AE, *E* and TEF for the photonic wire and dendrimer systems (Table 3, Fig. 6a) shows the dendrimers to be superior. The 3:1 dendrimer stands out with a TEF of >500 achieved using the sensitized emission of Cy5.5 in the linear 1.5 × *R*_0_ construct as a reference (left axis). These data also show that TEF can still be enhanced by ~12 × even in the linear 1.5 × *R*_0_ constructs despite three intervening FRET steps if donor wire number and geometry are optimized. This enhancement approaches 40 × at 0.5 × *R*_0_ dye spacing. If the sensitized emission of Cy5.5 from the linear 0.5 × *R*_0_ construct is used as a reference (right axis), a nearly 12-fold enhancement is observed in the 2:1 dendrimer along with a 40-fold enhancement in the 3:1 dendrimer, in a manner that exceeds a simple ratiometric relationship between initial donor(s) and terminal acceptor. This is a striking example of how DNA can pattern molecular dyes to markedly increase fluorescent output. Normalized emission spectra for the 0.5 × *R*_0_ 2:1 dendrimer (Cy3.5 phosphoramidite) and the eight-arm photonic wire star structures were directly compared (Fig. 6b), and although both have eight initial Cy3 donors and the eight-arm star has 10 more Cy3.5/Cy5 intermediary dyes the dendrimer still provides significantly enhanced terminal Cy5.5 sensitization. Comparison of the sensitization magnitude in the photonic wire and dendrimer systems (Fig. 6c,d) as energy is transferred stepwise and where dye emissions are scaled and normalized to the highest component in each again reflect the superior performance of the dendrimers, and especially the 3:1 construct.

### Förster analysis

To understand ET processes in these structures we undertook a detailed analysis similar to that of ref. 40 and described more thoroughly in the Methods (see also Supplementary Note 5, Supplementary Discussion). Given the complexities and sample uncertainties, our goal in modelling was not a perfect fit to data, but rather to semi-quantitatively address whether these systems are describable, in whole or part, by Förster theory, and whether designs perform as expected from first principles or show evidence of limitations. Förster analysis was applied to all constructs and overall we obtained a reasonably consistent interpretation. Three levels of simulation are considered with the first being an ideal simulation assuming ideal parameter values and perfect yield. The second levels are adjusted-parameter simulations accounting for possible discrepancies by plausibly small adjustments to dye spacings, *R*_0_, assembly yield or other parameters. Lastly, low-yield simulations are used when parameter adjustments are unable to fit the data, and we conclude that yield is deficient whether from assembly issues, self-quenching of dyes and so on. Simulated ensembles must then include various partial structures and leftover free dyes in addition to target structure. For brevity, only results for the photonic wire and dendrimer structures are summarized here (rest in Supplementary Figs 30–50, Supplementary Tables 60–64 and Supplementary Discussion).

**Figure 7:**
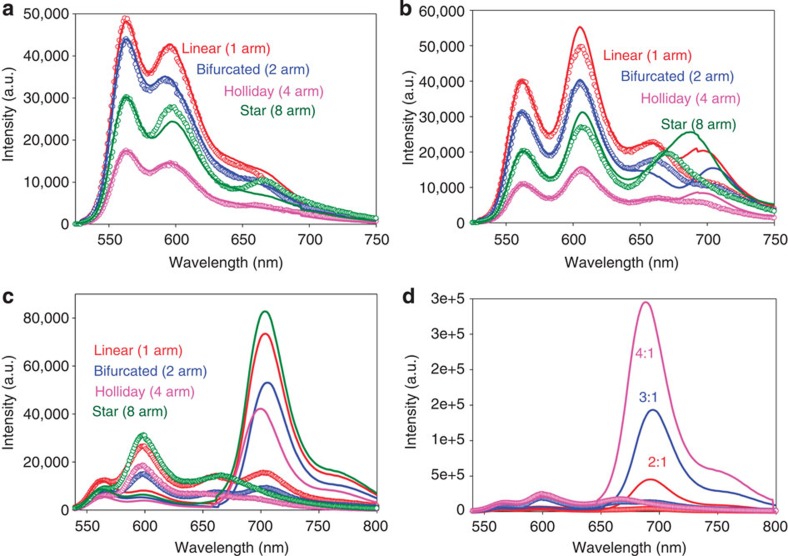
Energy transfer analysis. Comparison of experimental data (circles) with spectra predicted by ideal simulation (lines) for one-, two-, four-, and eight-arm [Cy3→Cy3.5→Cy5]_*n*_→Cy5.5 multi-dye structures with dye spacings of (**a**) 1.5 × *R*_0_, (**b**) 1.0 × *R*_0_ and (**c**) 0.5 × *R*_0_ and (**d**) for dendrimers with dye spacing of 0.5 × *R*_0_ and branching ratios of 2:1, 3:1 and 4:1, respectively. In general, it is observed that agreement between experiment and ideal simulations worsens as dye spacing gets smaller and structural complexity increases.

We began by comparing ideal simulations with experimental spectra for the four-dye photonic wire structures with one, two, four, and eight arms at 0.5 × , 1.0 × and 1.5 × *R*_0_, and for dendrimers (0.5 × *R*_0_) with branching ratios of 2:1, 3:1 and 4:1 (Fig. 7). When photonic wire dye spacing is 1.5 × *R*_0_ (Fig. 7a), ideal simulations are in excellent agreement with data, which is not surprising given both the weakness of the FRET processes and the better assembly obtained with these less dense structures. At 1.0 × *R*_0_ dye spacing (Fig. 7b), agreement is again good for Cy3 and Cy3.5 emission, but less so for the other dyes and especially Cy5.5. Finally, for the 0.5 × *R*_0_ dye spacings, ideal simulations of both photonic wires and dendrimers (Fig. 7c,d) completely failed to capture the observed spectra.

**Figure 8:**
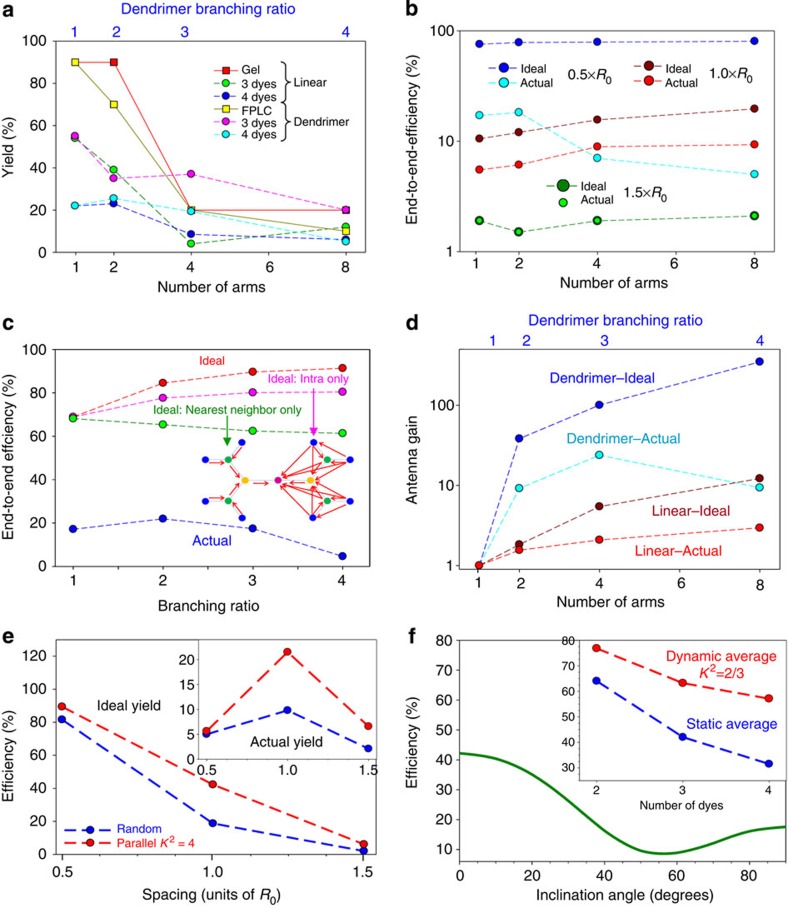
Ideal yield, efficiencies and dipole orientation. (**a**) Yield of target structures as assessed by gel electrophoresis or fast protein liquid chromatography (FPLC) for multi-dye photonic wire structures and dendrimers with 0.5 × *R*_0_ dye spacing (squares with solid lines) versus the number of arms or branching ratio, as compared with the corresponding yields derived from fitting the emission spectra when the structures are functionalized with either three or four dyes (circles with dashed lines). (**b**) Actual and ideal anywhere-to-end efficiency computed for the four-dye linear structures as a function of the number of arms with the dye spacing as a parameter. (**c**) Actual and ideal end-to-end efficiency computed for dendrimers as a function of branching ratio. Highlighting the importance of parallel FRET pathways, three ideal curves are shown, one assuming only nearest-neighbour FRET (schematically depicted inset, left), one including only intra-arm FRET (schematically depicted inset, right) and one including all FRET processes (ideal). (**d**) Actual and ideal antenna gains as computed for the four-dye linear structures and dendrimers with 0.5 × *R*_0_ dye spacing. Note, the 0.5 × *R*_0_ linear photonic wire structure corresponds to the one-arm dendrimer. (**e**) Effect of dipole orientation on FRET efficiency assuming ideal formation for the four-dye eight-arm star. Dipole orientation is either random (*κ*^2^=2/3) or for the parallel case dipoles on the same arm are coupled with *κ*^2^=4. Inset plot shows the same effect on efficiency assuming actual yield. (**f**) Anywhere-to-end efficiency as the inclination angle of the dyes with respect to the DNA axis varies for the four-dye eight-arm star at 1.0 × *R*_0_ dye spacing, assuming ideal yield. Inset plot shows the difference between dynamic (assuming *κ*^2^=2/3) and static averaging, assuming ideal formation for a 0.75 × *R*_0_ photonic wire assuming ideal yield as the number of dyes is increased from an initial Cy3–Cy3.5 configuration of two dyes with the addition of Cy5 (two dyes) and Cy5.5 (four dyes).

Parameter-adjusted simulations cannot account for the large discrepancies seen, and we conclude that the perfect yield assumption must be dropped, an inference that qualitatively matches our assembly efficiency data (Supplementary Tables 39 and 53). For low-yield modelling we took the simulated ensembles to be made up of target structure plus one or more partial structures, with all unincorporated dyes treated as free. For simplicity, we restrict partial structures to having each dye in full complement but with fewer dye types present, approximating the composite contribution of a wide variety of potential non-fully formed structures. With this approach, one obtains excellent agreement with experiment, and to interpret the results we compare target structure yields derived in this way (with three/four dyes) to those estimated from gel electrophoresis/FPLC (Fig. 8a). In general, yield characteristics for the photonic wire and dendrimer structures are similar and suggest a common failure mechanism. That yields for two-dye structures (not shown) and for total (target+partial) product for all structures are uniformly high indicates that the Cy3 and Cy3.5 dyes assemble with high fidelity (at the structures periphery) and that the observed non-ideal behaviour may be entirely due to Cy5 and/or Cy5.5. Moreover, since simulated yields with three- and four-dyes are similar, Cy5 becomes the likely culprit since it must function for downstream Cy5.5 to do so. The decline of assembly yield with increased structural complexity also suggests a crowding effect due to impaired hybridization and/or poor Cy5 properties/(self)quenching as noted before^40^,^58^,^59^. That performance did not improve when Cy5 was replaced with AF647 in the five-dye 2:1 dendrimer (Figs 1d and 5c) indicates assembly problems. We note that Cy5 position in the star and dendrimer structures is near the center, supporting the notion of problems with steric accessibility for it and the single Cy5.5 during assembly.

Presuming low-yield simulations constitute a plausible understanding of the system photophysics, we estimated their actual and ideal efficiencies along with gain parameters. When *E* is plotted for four-dye wires as a function of arm number, ideal results show the expected strong boost in efficiency as dye spacing is reduced (Fig. 8b). The ideal curves are relatively flat, which indicates that the arms act mostly independently, supporting our previous conjecture. Actual efficiencies are greatly reduced in the 1.0 × and 0.5 × *R*_0_ cases by the yield issues discussed. Comparing dendrimer *E* to the low actual values again highlight this discrepancy resulting from poor yield (Fig. 8c). In the ideal case, efficiency rises with increasing branching ratio by about 30%, although the 3:1/4:1 cases are not especially different. The reason for both the rise and saturation are the parallel paths in the structure. To investigate further, we examined additional simulations in which FRET was variously restricted (Fig. 8c). When only nearest-neighbour dye couplings were included (inset structure—left), no efficiency enhancements due to branching were observed. When couplings were instead restricted to dyes on the same branch (inset structure—right), a large fraction of the full ideal curve was realized. Thus, both intra- and inter-branch parallel paths contribute to efficiency enhancement making the dendrimers inherently more efficient than the photonic wire constructs, where the arms act largely independently. Next, we look at the antenna properties using an antenna gain (AG) metric analogous to TEF but relative to the equivalent linear photonic wire (that is, equal dye spacing, Supplementary Note 2). Comparing ideal and actual AG for the four-dye photonic wire and dendrimer structures (Fig. 8d) shows that the ideal curve for the wires is close to the unity slope expected if all arms operated independently; the slightly higher slope reflects a small contribution from parallel paths. Actual AG is much lower, again because of the yield. For the dendrimers, we see potential for dramatic (exponential) increases in collection with the 4:1 structure ideally producing a gain of ~400. Yield again causes the AG realized to be worse, with the 4:1 dendrimer AG exhibiting a decline.

## Discussion

Focusing on the photonic wire and the dendrimer systems (Table 3), performance generally trends upward with increases in initial donor absorption profile and collection area, (*ε*_initial donor_)_*n*_, reflecting basic antenna properties. However, increases are not continuous as issues plague the more complex structures. Net exciton delivery efficiency *E* over three or four FRET steps is substantially improved compared with that in our previous work^39^,^40^, however, this is qualified by significant structural, directional (that is, inward versus outward focusing) and material differences. On their own, an *E* of ~20% across four-FRET steps combined with AE of 3.9 and TEF of 550 (*albeit* going from a linear 1:1 stoichiometry at 1.5 × *R*_0_ to multi-donor dendrimers at 0.5 × *R*_0_, Table 3) reflect the potential these architectures have for understanding and improving energy flow in FRET networks. To better understand the underlying FRET, we undertook numerical simulations of exciton transport using Förster theory, which, insofar as can be determined from the consistency of the overall interpretation, seems to be an appropriate basis. The simulated ideal characteristics show there is much room for improvement, with observed shortfalls likely arising from assembly formation inefficiencies, dye photophysical performance and dipole orientation. Further investigation was undertaken to understand the extent to which these contributed.

Our experimental approach did not include DNA assembly purification but instead looked at ensemble properties of unpurified product. Our structures are relatively simple in the number of oligos per construct, which both simplifies experiments and complicates analysis. This was necessitated by the difficulties of separating these fully formed products from partial structures of similar size and charge. In contrast, when using an origami assembly-based approach, separation from excess staple strands is easier due to the dramatic difference in size of the final assembly, but the cost associated with hundreds of different oligos for each construct and the need for excess dye-labelled staples made this approach impractical (cost prohibitive). Far denser dye-networks are probably achievable on origami. It should be noted, however, that insight into one of our more interesting and potentially important findings, that multiple redundant FRET pathways compensate for assembly deficiencies in the dendrimer architecture (*vide infra*), would not have been provided with use of only purified structures regardless of the assembly method. Following a first pass of construct formation, we did attempt to improve FRET *E* and formation efficiency through optimization of hybridization. Several different hybridization permutations were attempted with select linear and dendrimer structures, with improvements only seen for two of the dendrimers. Increasing to 12 h hybridization resulted in a doubling of the 3:1 and 4:1 dendrimer *E* values although FPLC analysis revealed assembly was only ≤10% more efficient (data not shown), suggesting improvement in placement of key internal FRET dye components (that is, the aforementioned Cy5 and Cy5.5). Data values of the latter are only used here for brevity. No improvements to the 2:1 dendrimer assembly or *E* were seen. Systematic-parametric testing of hybridization protocols could certainly help improve formation efficiency for a desired structure, however, this was beyond the current scope due to the variety of different systems involved.

We next examined dye photophysical performance. Since modelling and literature reports indicate Cy5 can exhibit photophysical properties detrimental to FRET^40^,^58^,^59^, A647 substitution in the five-dye 2:1 dendrimer (Fig. 5c, Supplementary Figs 27 and 28) was tested but did not improve efficiency, although A647 has previously demonstrated appreciable FRET-sensitized properties^54^. Other reports indicate that constraining cyanine dye movement may help enhance fluorescence^60^, we thus altered the Cy3.5 in the [[Cy3_2_→Cy3.5]_2_→Cy5]_2_→Cy5.5 four-dye 2:1 dendrimer from ester attachment via a flexible alkyl linker (QY~0.15) to a more rigid version by using a double phosphoramidite insertion (QY~0.30, this chemistry only became available recently). This improved *E* and AE by ~60% and 65%, respectively (Fig. 5c). We note, paradoxically, that Cy3.5 in the 0.5 × *R*_0_ bifurcated [Cy3→Cy3.5→Cy5]_2_→Cy5.5 wire system, which demonstrated unity FRET (Fig. 4e), was attached through a flexible alkyl linker (QY~0.15), whereas the Cy5 dyes utilized here were almost exclusively constrained phosphoramidites. Moreover, Cy5 (as an ester) has demonstrated excellent acceptor properties in a previous multichromophore DNA wire^49^.

Lastly, the issue of dipole orientation and its potential contribution is complex especially when considering multipathway dendrimer structures. Even with perfect control, the effect of dipole orientation (*κ*^2^) is relatively small, primarily because of the 1/6 power dependence over separation, which in the best case of parallel dipoles results in *R*_0_ being only ~35% larger than when randomly oriented. To examine the impact of having oriented dipoles on the anywhere-to-end efficiencies of FRET networks more closely, we compared simulations with oriented dipoles to results obtained for the dynamically averaged random dipoles (*κ*^2^=2/3) limit; the latter were used for all other simulations (Fig. 8e). This example studies an eight-arm star network with three dyes (Cy3, Cy3.5 and Cy5) on each arm and a single-central Cy5.5 dye. The efficiency versus dye spacing when dipole angles are assumed random (*κ*^2^=2/3) is compared with that obtained when optimally oriented parallel to the DNA axis of each arm. Since these arms are allowed to bend out of the plane, the optimal dipole orientation will be maintained only within a given arm and not between arms. The main plot (Fig. 8e) assumes ideal formation efficiency, while the inset assumes actual formation efficiencies. In both, dipole orientation has the biggest effect when dye spacing nears 1.0 × *R*_0_ because this is where Förster coupling is most sensitive to all parameters. Critically, this observation represents another reason why dipole orientation is a secondary consideration in FRET network design in that one typically looks to maximize the FRET efficiency of a network by decreasing dye spacing, and as the analyses show, the importance of the dipole factor drops as the spacing falls below 1.0 × *R*_0_.

Achieving parallel dipole orientations in a photonic wire is unlikely given the flexible linker attachments. To study misalignment effects, we examined anywhere-to-end efficiency for the [Cy3→Cy3.5→Cy5]_8_→Cy5.5 1.0 × *R*_0_ star as a function of dipole angle relative to DNA axis (Fig. 8f). Here the azimuthal angle is random so that as the dipoles incline away from the DNA axis they go out of parallel alignment. Nevertheless, if the misalignment is <20° the effect on efficiency remains small. Lastly, representation of random dipoles by the averaged *κ*^2^=2/3 assumes that dipole re-orientation time is fast compared with lifetime; referred to as dynamic averaging. The opposite limit occurs when dipole re-orientation time is slow compared with lifetime so that the dipoles will be random but fixed in orientation. The averaging that occurs is then over the ensemble and is referred to as static averaging^61^,^62^. In general, these averages differ and which is most relevant (or if the actual lies between these two limits) is not clear. We assume dynamic averaging is appropriate; however, for insight we also simulated photonic wires where dynamic and static averaging are compared (Fig. 8f inset). This difference can have a significant effect, with static averaging generally lowering efficiency. There have been elegant demonstrations of controlling dipole orientation in linear DNA duplexes with custom synthesized dyes^63^, however, such control would still not apply to interactions between different arms or dendrimer pathways. Clearly, understanding dipole contributions in such complex geometries needs far more attention. Overall, assembly efficiency, dye performance, and dipole orientation will contribute (sometimes unpredictably) with the magnitude of each depending on the complexity of a desired DNA-based FRET network. Nevertheless, complex-efficient FRET networks can still be achieved.

Our results suggest potential rules-of-thumb regarding design of DNA-based FRET networks. Dye spacing is the most profound consideration, especially in comparison to dipole orientation given the lack of control over the latter within a network and the smaller magnitude of its effects. Attempting to improve *E* by increasing donor number only increases FRET to a finite point since it only increases the probability that FRET will happen and not the efficiency of that transfer step (assuming unchanged distances)^54^. Multiple donors at further distances can only partially compensate for fewer donors with closer separation (see two-dye results, Fig. 3c). Nevertheless, if a given structure forces separation constraints, improvements can be accessed even at 1.5 × *R*_0_. For example, in the two-dye system (Fig. 3c), AE and TEF increase three and four times, respectively, when going from one to four donors at 1.5 × *R*_0_ and this increases to 3 and 6.3 times at 1.0 × *R*_0_, respectively. Controlling fluorophore positioning relative to other donors–acceptors in a given network is important. Asymmetry significantly affects performance as shown in the two-dye eight-arm star system (Fig. 3d). Moreover positional uncertainty will have a diminishing contribution as structures become larger and denser; dye-to-dye spacing again becomes far more important here. Fluorophore performance will remain a potential limiting factor and may necessitate empirical testing. Although, important work has provided insight into this issue^42^,^43^, it still remains unpredictable. This is underscored by a recent example where a dye with a QY of 90% acted as a localized quencher within a DNA photonic wire^64^. However, enough structural diversity now exists across dye families that many common donor–acceptor alternatives are available^52^. As shown with Cy3.5, examples can also be encountered where dye emission will increase on the DNA^44^,^48^,^60^. For optimal efficiency, having multiple parallel FRET pathways interacting is significantly better than if they act independently. One reason is the direct improvement in efficiency that comes with more paths (Fig. 8d) with possible contributions from homoFRET (Supplementary Fig. 39, Supplementary Discussion). Redundancy in the interacting paths makes the structure better able to tolerate defects when assembly issues arise. This may explain the improved performance seen in the 2:1, 3:1 and 4:1 dendrimers (*E* ~17%, 23%, 8%; AE ~220, 390, 160 and TEF ~150, 520, 340, respectively, for the Cy3.5 ester versions), despite poor assembly (~70%, 20% and 10%, respectively). There is only a small gain in ideal *E* when going from a 3:1 to 4:1 dendrimer (Fig. 8c) and full assembly yield between these structures is not that different; however, the 3:1 architecture shows far better performance, suggesting the correct trade-off between number of FRET pathways and mass conversion. Assembly deficiencies are also more severe with the complex multi-arm or dendrimeric structures. It is important to again point out that use of alternate structures (that is, origami-based) or purification could help address this. The five-dye 2:1 dendrimer (Fig. 5b) represents perhaps the best functional compromise among all these issues as it is structurally simple (10 oligos), has high assembly yield (~60%), while providing *E* (16–19%) comparable to the four-dye dendrimers even with an extra FRET step. Finally, controlling dipole orientation remains challenging. For a single-FRET step, a random static distribution of dipoles can limit energy transfer efficiency because more ways to achieve relatively poor dipole–dipole orientations exist than favourable orientations. This penalty accrues with the length of the FRET cascade (Fig. 8f), and is anticipated to be important for denser networks like dendrimers, where spatially constrained dyes have less freedom to undergo dynamic averaging.

Despite all these issues, when compared with using alternatives such as chemically synthesized dendrimers or macromolecular protein scaffolds to create FRET networks^11^,^65^,^66^, the ability of DNA to rapidly prototype almost any configuration of dye positions provides a powerful tool for studying FRET network performances as recently discussed^67^. Here, we exploit this approach to construct FRET dye networks of unprecedented complexity. We realize that the work here represents only a small fraction of what should be possible, given the rich space of possibilities available using DNA structures. Extensions could include larger networks assembled on origami incorporating far more dyes/FRET steps, increased ranges of donor–acceptor ratios and incorporation of other nanomaterials such as QDs, whose inherent light harvesting and nanoplatform/display capabilities have much to offer in this context^39^,^40^,^68^,^69^. Another exciting avenue involves putting such structures to work for biosensing, charge conversion, molecular logic and computing or even as focusing networks built around a biocompatible scaffold for sensitizing photodynamic therapy agents^2^,^8^. Given their ability to tolerate defects, it is perhaps here amongst targeted applications where DNA-based biophotonic networks may have the most to offer.

## Methods

### Materials

Labelled and unlabelled DNA were obtained from Integrated DNA Technologies (Coralville, IA, USA) with the exception of the Cy3.5 and internal labelled Cy5.5-functionalized strands, which were purchased from Operon Biotechnologies, Inc. (Huntsville, AL, USA). 2.5 × PBS (342.5 mM NaCl, 25 mM phosphate and 6.75 mM KCl) was diluted from a 10 × DNAse/RNAase-free stock purchased from Sigma-Aldrich (St Louis, MO, USA). DNAse/RNAase-free water was also obtained from Sigma-Aldrich. Black Corning 96 and 384 well nonbinding surface microtitre plates were obtained from Sigma-Aldrich.

### Sample assembly and hybridization

Stock solutions of DNA were diluted into 2.5 × PBS at 20 μM working concentration. Individual samples were assembled stepwise from component DNA (20 μM) in 0.5 ml PCR or 1.5 ml Eppendorf tubes to a final concentration of 1 μM in ~110 μl of 2.5 × PBS. This concentration range was empirically tested to match the linear portion of fluorescent response in later instrumental data collection, see Supplementary Methods and Supplementary Fig. 52. This high salt concentration is used to raise DNA melting temperatures. MgCl_2_ was excluded due to the potentially deleterious effects high concentrations of such ions can have on dye fluorescence^52^. Use of high salt concentrations (such as 2.5 × PBS) to maintain DNA structures without MgCl_2_ has been previously validated^53^. Samples were vortexed repeatedly, microfuged and then placed in a heating block with boiling water in the wells. The block was removed after 1 min and the samples were allowed to cool to ambient temperature for 2 h followed by brief microcentrifugation to collect the volume and 1 h incubation at 4 °C. A similar procedure was also used substituting a PCR thermal cycler for the heating block.

For the 2 h PCR-based annealing conditions used for the dendrimer structures, solutions were annealed on an Eppendorf Mastercycler Thermal cycler. The standard annealing conditions were 95 °C for 4 min with a 1 °C ramp down per minute until 4 °C was reached. For the 12 h annealing condition, the same thermal cycler was used, but the program was modified such that it was held at 95 °C for 5 min and ramped down to 60 °C at a rate of 1 °C for every 4 min. The temperature was held at 60° for 2 h. The temperature was ramped down again to 50° at a rate of 1 °C per 4 min and held at 50 °C for 2 h. A final ramp at 1 °C every 4 min was done until a final temperature of 4 °C was reached. The hold temperatures of 60° and 50° were chosen as they are just below the average melt temperature of the medium- and short-length strands, respectively. Replicate structures were assembled for the full constructs and all control permutations thereof with one or more dyes missing to estimate other FRET pathways.

### Data collection

Each structure to be tested was typically independently assembled at least in triplicate, which usually entailed performing experiments over several days. Assembled structures were aliquoted into microtitre well plates and fluorescence was collected on a Tecan Infinite M1000 Dual Monochromator Multifunction Microtiter Plate Reader (Tecan, Research Triangle Park, NC, USA) equipped with a xenon flash lamp using 515 nm excitation for Cy3 and 465 nm for AF488. 400 Hz flash frequency was used with a 40 μs integration time. Emission spectra were collected from 530 to 850 nm in 1 nm increments and exported into an Excel spreadsheet for later data processing and analysis. The Tecan performs an automatic, calibrated adjustment on collected data for nonlinear detector response in the near-infra red, that is, internally corrected. spFRET analysis is described in Supplementary Methods.

### Förster distances

Donor emission, acceptor absorption and molar extinction coefficients of each dye were used to calculate the spectral overlap integral, *J(λ)*, for FRET conjugates. The Förster distance (*R*_0_), corresponding to donor–acceptor separation resulting in 50% energy transfer efficiency was also calculated for each donor–acceptor pair using the expression^50^:


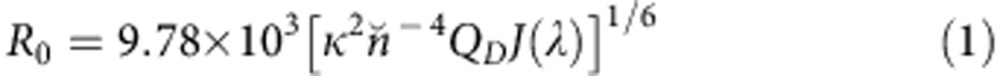


Where, 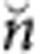 is the refractive index of the medium, *Q*_D_ is the fluorescence quantum yield (QY) of the donor, and *κ*^2^ is the dipole orientation factor that we often, but not always, take to be 2/3 as is appropriate for the quasi-random dipole orientations characteristic of these constructs^50^.

### Spectral decomposition

Each construct was processed by collecting spectra from each configuration along with the direct excitation components of each dye; the latter (except for the initial donor) were then subtracted from the composite. Finally, corrected spectra were decomposed into component parts corresponding to the quenched initial dye and sensitized remaining dyes. See Supplementary Methods and Supplementary Fig. 53 for a representative example of this process. Composite PL spectra of fluorophore-DNA FRET constructs were fit with a series of Gaussian curves using the Multipeak Fitting tool in Igor Pro (version 6.31)^39^,^40^,^70^. The series of Gaussian peaks were then fit to the experimental data via minimization of the *χ*^2^-values. Satisfactory fits were confirmed by residual plots, which consisted of the fit data subtracted from the raw data, and were reduced to baseline noise with respect to the experimental data sets. The decomposed components of the best-fit curves were numerically integrated to quantify the PL contribution from individual fluorophores within a given construct. These data were then used to determine several parameters including donor PL loss, acceptor sensitization, *E* and TEF. The general approach closely follows the analysis of FRET-based DNA photonic wires previously reported^39^,^40^.

### End-to-end energy transfer efficiency

The end-to-end energy transfer efficiency, *E*, of each construct was calculated using the expression^37^,^39^,^40^:


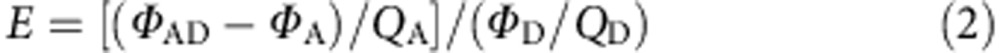


where *Φ*_AD_ is the integrated PL area of the terminal acceptor in the presence of donor, *Φ*_A_ is the integrated PL area of the terminal acceptor in the absence of donor, *Φ*_D_ is the integrated PL area of the donor in the absence of acceptor, and *Q*_A_ and *Q*_D_ are the QY of the terminal acceptor and donor, respectively. *E* provides a means to assess the terminal acceptor re-emission following sensitization from an upstream fluorophore, while also accounting for the QY of the initial donor and terminal acceptor. The values of *Φ*_D_ and *Φ*_A_ were determined by numerical integration of PL area fits from molar equivalent samples containing only the donor or acceptor of interest, respectively. In multiFRET step constructs *Φ*_AD_ in equation (2) remains the integrated PL intensity of the terminal acceptor in the presence of the primary FRET donor.

In constructs possessing multiple donors, *E* accounts for energy that has entered the system through the initial donor as well as from direct excitation of intermediary fluorophores serving as donors. For example, in full Cy3–Cy3.5–Cy5–Cy5.5 constructs the majority of energy is introduced to the system via direct excitation of Cy3. However, some energy is introduced through direct excitation of Cy3.5 and to a lesser extent Cy5. Therefore, the magnitude of *E* reflects the amount of terminal acceptor sensitization arising from energy that has entered the system through any upstream fluorophore, of which the Cy3 emission provides the significant majority.

### FRET donor efficiency and acceptor sensitization

A similar analysis was employed to quantify the average FRET donor efficiency, *E*_D,_ and acceptor re-emission efficiency, *E*_A_, for each donor–acceptor pair within a particular construct. Direct excitation spectra fits for each fluorophore (determined from molar equivalent control samples) were subtracted from fit emission data sets to minimize contributions to the composite PL intensity arising from direct excitation of fluorophores subsequent to the initial donor. Following the scaling/subtraction method outlined above, the resulting spectra were decomposed to determine the contributions from the primary donor emission and the sensitized acceptor contribution in a particular configuration. These data were numerically integrated and used to calculate *E*_D_ and *E*_A_ for each donor–acceptor conjugate according to^50^:


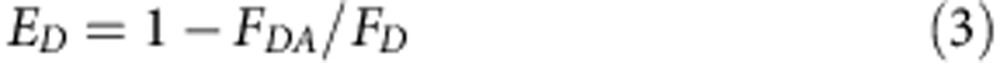


and


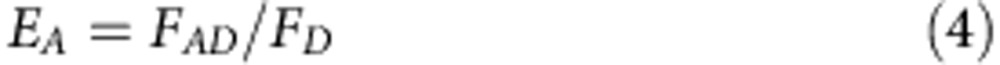


where *F*_DA_ is the integrated PL area of the donor in the presence of acceptor, *F*_D_ is the integrated PL area of the donor in the absence of acceptor, and *F*_AD_ is the integrated PL area of the acceptor in the presence of donor. Equation 4 is simplified here and must also take into account the quantum yields of the donor and acceptor as in Equation 2. In constructs with multiple fluorophores, the efficiency for each donor–acceptor pair is analyzed as an independent step, regardless of whether the donor was directly excited and/or sensitized.

### Antennae effect

The antennae effect (AE) was measured for all systems and is defined here as^30^:


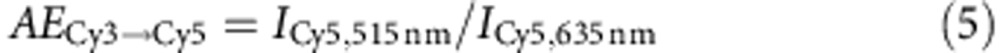


and


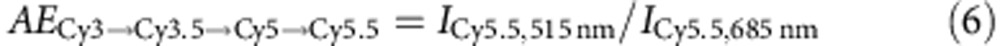


where *I*_Cy5,515 nm_, *I*_Cy5,635 nm_ and *I*_Cy5.5,515 nm_, *I*_Cy5.5,685 nm_ are the fluorescence intensities (decomposed area under the curve) of the terminal Cy5 or Cy5.5 following excitation of the initial Cy3 donor at 515 nm and direct excitation at 635/685 nm, respectively. AE are given as amplification factors.

### Terminal enhancement factor

TEF was introduced to allow for a comparison of the PL intensity of a terminal acceptor (Cy5 or Cy5.5) across all FRET constructs regardless of geometry (linear, bifurcated, Holliday junction, eight-arm star and dendrimers) or donor number. First, PL data for a given construct were normalized by the intensity of the Cy3-DNA conjugate since molar equivalence was maintained across all data sets. This accounted for any instrument variation during data collection. Next, a scaling factor was introduced to account for presumptive number of active Cy3 dyes within a particular construct: 1 for linear, 2 for the bifurcated, 4 for the Holliday junction, 8 for the eight-arm star and 2:1 dendrimer, 27 for the 3:1 dendrimer and 64 for the 4:1 dendrimer. The PL intensity of terminal acceptor, determined from decomposition of composite spectra, was subjected to this normalization and scaling procedure and tabulated. Finally, the terminal acceptor PL intensity with the lowest value (unidirectional, 1.5 × *R*_0_) was then set to unity and all other data scaled up by this value. The result is a series of normalized data points reporting the terminal acceptor PL intensity accounting for the various linear and dendrimeric constructs. TEF is applied regardless of donor number, configuration or donor–acceptor separation. TEF was determined for the two-dye and four-dye constructs by:


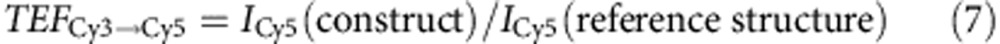



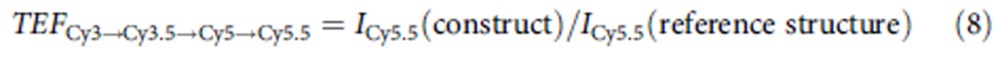


where *I*_Cy5_ and *I*_Cy5.5_ represent the processed net sensitization components for a particular construct, that is, linear, bifurcated and so on, and reference structures determined as described for the respective two-dye and photonic wire or dendrimer systems.

### Förster analysis

To better understand underlying ET processes in these structures we undertook a detailed analysis similar to ref. 40 and described thoroughly in Supplementary Note 5 and in the Supplementary Discussion. Since we sometimes treat target structures as incompletely formed and accompanied by assorted partial constructs/free dyes, we normalize the governing rate equations by total concentration and the variables then become equivalent to probabilities. For steady state, only the time-integrated probability *W*_*ik*_ that the *i*th dye on the *k*th construct will be excited is needed, and one can show that this obeys:


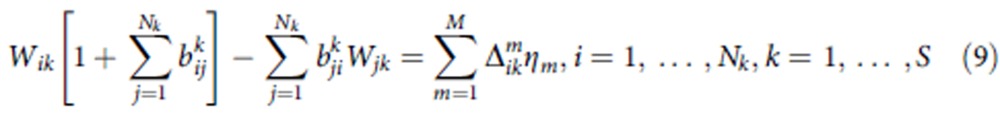


where *S* is the number of different constructs in the ensemble, *M* is the number of different types of dyes, *N*_*k*_ is the number of dyes in the *k*th construct, 
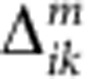
 is unity only if dye *i* on construct *k* is of type *m*, and *η*_*m*_ is the probability that an absorbed photon creates an exciton on dye *m* (Supplementary Note 5). The matrix element 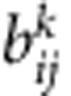 specifies the excitonic coupling between dyes *i* and *j* on construct *k*, and according to Förster theory it varies as 1/*r*^6^ (*r*=inter-dye distance). The quantities in equation (9) are related to the PL areas *Φ*_*m*_ by


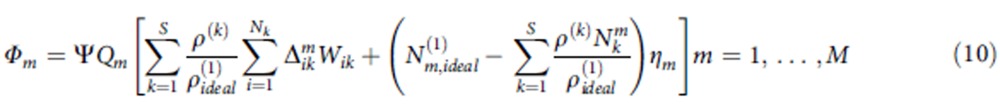


where *Q*_*m*_ is the quantum yield of dye *m*, *ρ*^(*k*)^ is the molar concentration of construct *k*, 
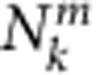
 is the number of dyes of type *m* in construct *k*, and Ψ is a scaled generation rate that like the *η*_*m*_ can be estimated from control experiments (Supplementary Note 5). Of the parameter values needed for the simulations (Supplementary Note 5), some are reasonably well-established, for example, *Q*_*m*_ and *R*_0_ (Table 1), while others like *r* are not. Rough estimates of *r* for nearest-neighbour dyes can be obtained from distances between dye attachment points as dictated by DNA design. For better accuracy, one must also account for small distances between attachment points and dyes as set by linkage chemistries and dye structures (Supplementary Fig. 51 and Supplementary Note 5). Although sophisticated correction methods exist^22^, given the number/complexity of the situations considered, we instead assert reasonable values and look for validation in the results obtained, when distances are held fixed across all structures having the same dyes/linkage chemistries. For non-nearest-neighbour dyes, things are more complicated because the interconnecting DNA scaffold can bend at flexible junctions, and we treat these by assuming they can take random angles over a specified range, and capture the aggregate effect through ensemble averaging over many configurations in three dimensions. In the case of the four-/eight-arm Holliday/star structures, we represent their central openings (Fig. 1) crudely as DNA rings to which Cy5.5 is attached and the DNA arms are allowed to take random angles.

## Author contributions

S.B.-W., M.G.A., W.R.A., E.R.G. and I.L.M. conceived the experiments and designs. All authors performed experiments and analyses of the data. S.B.-W, M.G.A., C.M.S. and I.L.M. wrote the paper with inputs from all authors.

## Additional information

**How to cite this article:** Buckhout-White, S. *et al*. Assembling programmable FRET-based photonic networks using designer DNA scaffolds. *Nat. Commun.* 5:5615 doi: 10.1038/ncomms6615 (2014).

## Supplementary Material

Supplementary Information Supplementary Figures 1-53, Supplementary Tables 1-67, Supplementary Notes 1-5, Supplementary Discussion, Supplementary Methods and Supplementary References.

## Figures and Tables

**Table 1:** Fluorophore photophysical and FRET properties.

					***R***_**0**_ **in Å/*J*(*****λ*****) in cm**^**3**^ **M**^−1^*					
**Fluorophores**	**QY**	**Ext. coeff.(M**^**−1**^**cm**^**−1**^**)**	***λ***_**max**_**abs. (nm)**	***λ***_**max**_**em. (nm)**	**AF488**	**Cy3**	**Cy3.5**	**Cy5**	**AF647**	**Cy5.5**
AF488	0.39	71,000	495	519	46/1.23e^**−**13^	61/6.94e^−13^	59/5.93e^−13^	49/1.90e^−13^	47/1.42e^−13^	43/8.92e^−14^
Cy3	0.15	150,000	550	570	—	47/3.68e^−13^	53/8.01e^−13^	54/9.37e^−13^	53/7.83e^−13^	49/4.73e^−13^
Cy3.5†	0.15	150,000	581	596	—	—	44/2.70e^−13^	60/1.69e^−12^	59/1.58e^−12^	55/1.01e^−12^
Cy5	0.28	250,000	649	670	—	—	—	65/1.39e^−12^	—	68/1.94e^−12^
AF647	0.33	239,000	650	665	—	—	—	—	65/1.17e^−12^	72/2.18e^−12^
Cy5.5	0.23	190,000	675	694	—	—	—	—	—	63/1.41e^−12^

abs., absorption; em., emission; Ext. coeff., extinction coefficients; QY, quantum yield.

^*^Förster distance (*R*_0_) and spectral overlap integral *J*(*λ*) are averages calculated from the spectra of all dye-labelled DNA used.

^†^QY of Cy3.5 phosphoramidite in Fig. 5c is ~0.30.

**Table 2:** Antenna effect and end-to-end efficiency for the Cy3–Cy5 single-FRET step system.

			**Relative Förster distance (** × *******R***_**0**_**)/predicted donor–acceptor separation**				
			**0.75/40.5 Å**	**0.85/45.9 Å**	**1.0/54 Å**	**1.25/67.9 Å**	**1.5/81 Å**
**Construct**	**Cy3/Cy5**	***ε***_**Cy3*****n***_**/*****ε***_Cy5_*	**AE/*****E*** **(%)**	**AE/*****E***	**AE/*****E***	**AE/*****E***	**AE/*****E***
Linear	1	0.6	1.1/40	0.8/22	0.5/18	0.3/9	0.2/3
Bifurcated	2	1.2	1.9/51	1.3/28	0.8/20	0.6/10	0.3/4
Holliday junction	4	2.4	2.9/37	1.2/30	1.4/23	0.8/9	0.3/4
Eight-arm star	8	4.8	2.5/15	1.6/16	1.8/15	0.8/8	0.5/2

AE, antenna effect; *E*, end-to-end efficiency.

All values are collected from at least three independently assembled structures. S.d. for AE and *E* values from replicate experiments are all <10%.

^*^Initial Cy3_*n*_ absorption at 550 nm relative to the final Cy5 absorption at 650 nm.

**Table 3:** Antenna effect and end-to-end efficiency for the four-/five-dye photonic wire and dendrimer systems.

			**Relative Förster distance**		
			**0.5 × *****R***_0_*†	**1.0 × *****R***_**0**_	**1.5 × *****R***_**0**_
**Construct**	**Wires**‡**/Cy5.5**	***ε***_**Cy3*****n***_**/*****ε***_**Cy5.5**_§	**AE/*****E*** **(%)**	**AE/*****E***	**AE/*****E***
Linear	1	0.8	1.8/16	0.5/4	0.2/2
Bifurcated	2	1.6	2.9/14	0.9/7	0.1/1
Holliday junction	4	3.2	1.1/9	0.7/9	0.2/3
Eight-arm star	8	6.3	1.1/6	0.8/6	0.3/2
**Four-dye**	**Cy3/Cy5.5**	—	—	—	—
2:1 dendrimer	8	6.3	2.2/17	(Cy3.5—ester)	
2:1 dendrimer	8	6.3	3.5/28	(Cy3.5—phosphoramidite)	
3:1 dendrimer	27	21.3	3.9/23	—	—
4:1 dendrimer	64	50.5	1.6/8	—	—
**Five-dye dendrimer**	**AF488/Cy5.5**	***ε*_AF488*n*_/*ε*_Cy5.5_**||	—	—	—
2:1 (Cy5)¶	16	6	1.8/19	—	—
2:1(AF647)¶	16	6	1.4/16	—	—

AE, antenna effect; *E*, end-to-end efficiency (in some cases this is utilized as anywhere-to-end efficiency).

All values are collected from at least three experiments of independently assembled structures.

^*^See Table 1 for individual dye–dye donor–acceptor *R*_0_ values.

^†^S.d. of all values <10%.

^‡^Wire=[Cy3→Cy3.5→Cy5]_*n*_.

^§^Initial Cy3_*n*_ absorption at 550 nm relative to the final Cy5.5 absorption at 700 nm.

^||^Initial AF488_*n*_ absorption at 550 nm relative to the final Cy5.5 absorption at 700 nm.

^¶^Displaying either Cy5 or AF647 at the fourth dye position.
