# Supplementary material for: Assembling programmable FRET-based photonic networks using designer DNA scaffolds
Source: Nat Commun. 2014 Dec 11;5:5615. doi: 10.1038/ncomms6615 (PMC4275599; doi:10.1038/ncomms6615)
Supplement: Supplementary Information — Supplementary Figures 1-53, Supplementary Tables 1-67, Supplementary Notes 1-5, Supplementary Discussion, Supplementary Methods and Supplementary References. [file ncomms6615-s1.pdf]

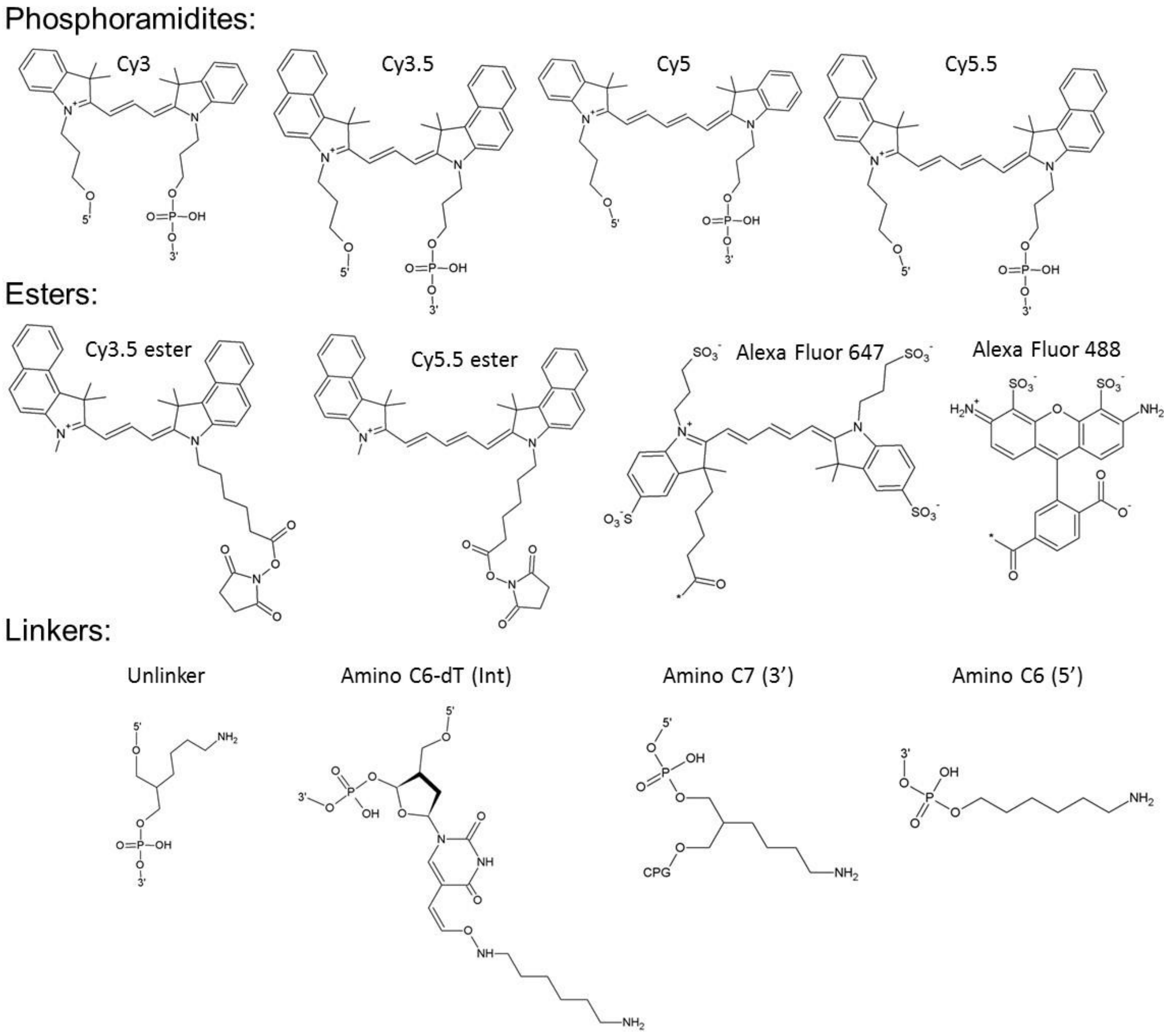

Supplementary Figure 1. Chemical structures of the organic dyes. The top row dyes are inserted directly using phosphoramidite chemistry. In this case the dyes are placed between 2 bases and are opposite an unpaired A base or just end-labeled. The middle column has structures of dyes inserted using succinimidyl ester chemistry. These dyes are attached to one of the amine-modified linkers (last row) placed at either the 5', 3' or inserted internally.

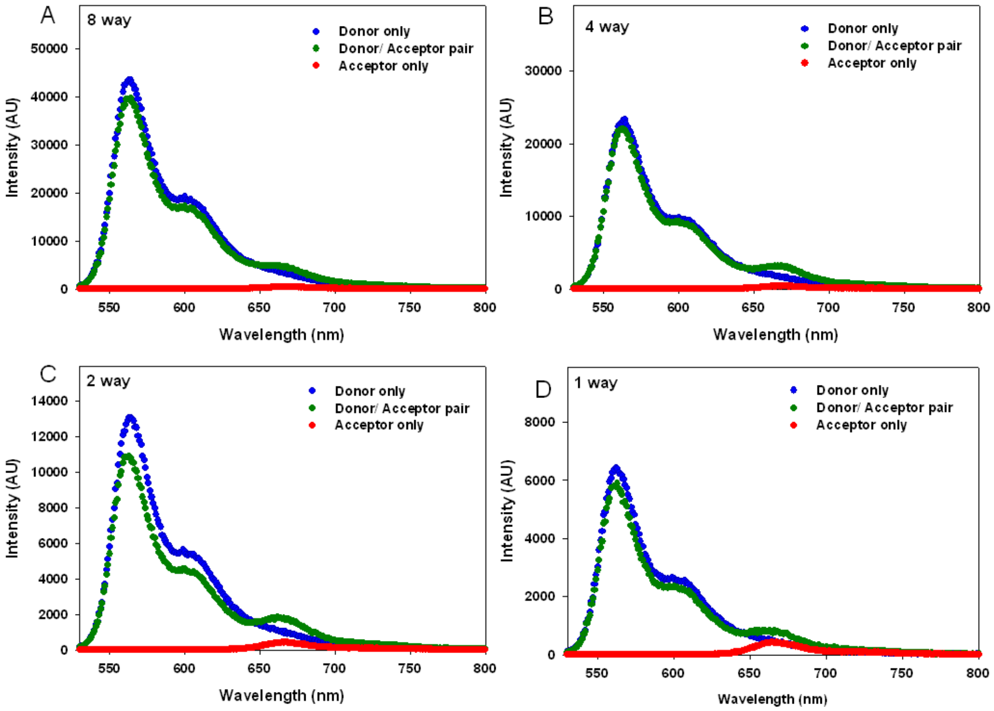

Supplementary Figure 2. Raw data for the 2-dye 1.5×R_0. Data shows the Cy3 donor alone, the Cy3-Cy5 donor acceptor pair and the Cy5 acceptor only plots. (**A**) 8-way (**B**) 4-way (**C**) 2-way (**D**) 1-way.

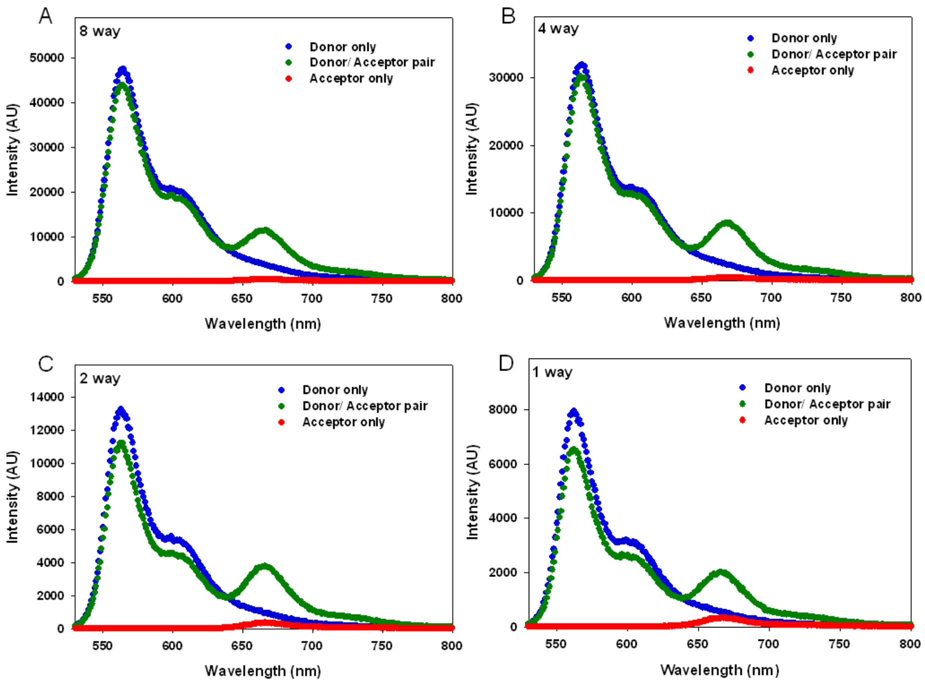

Supplementary Figure 3. Raw data for the 2-dye 1.25×R_0. Data shows the Cy3 donor alone, the Cy3-Cy5 donor acceptor pair and the Cy5 acceptor only plots. (**A**) 8-way (**B**) 4-way (**C**) 2-way (**D**) 1-way.

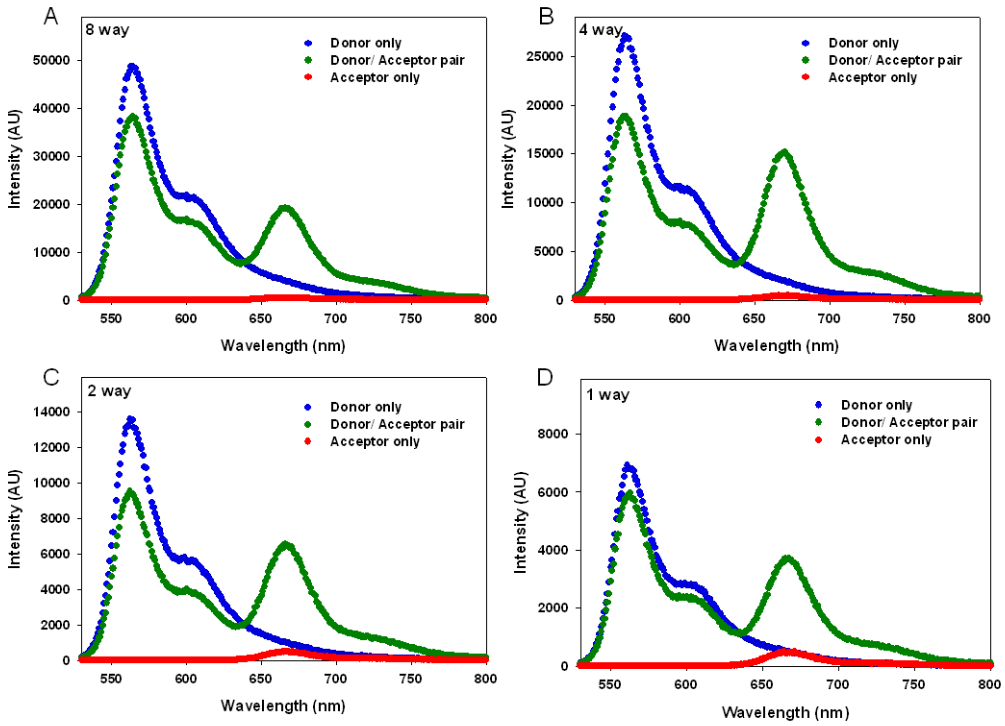

Supplementary Figure 4. Raw data for the 2-dye 1.0×R_0. Data shows the Cy3 donor alone, the Cy3-Cy5 donor acceptor pair and the Cy5 acceptor only plots. (**A**) 8-way (**B**) 4-way (**C**) 2-way (**D**) 1-way.

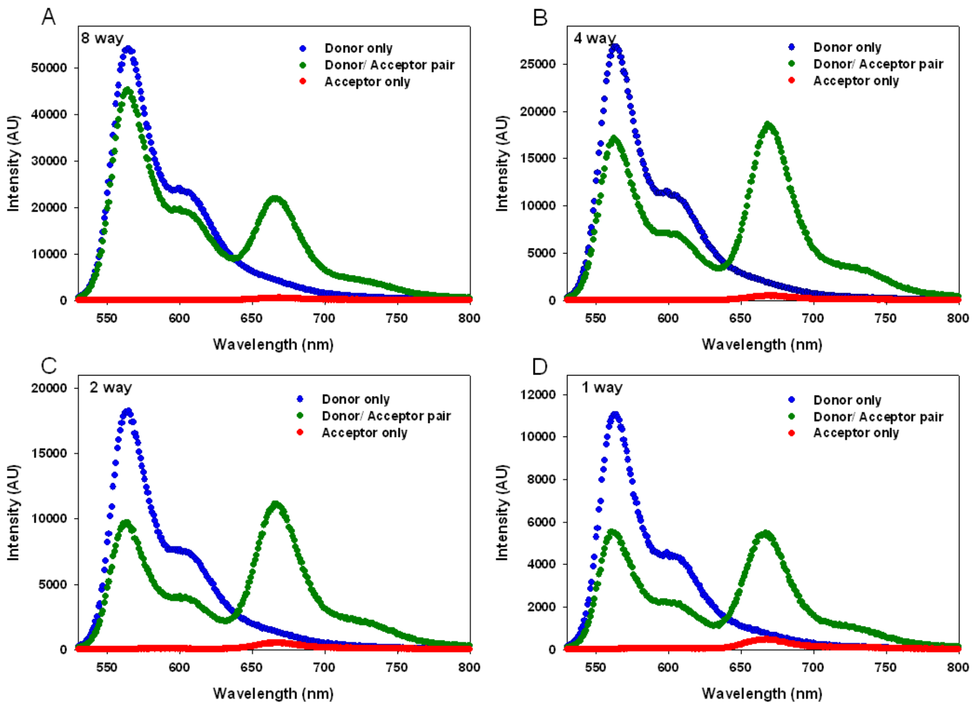

Supplementary Figure 5. Raw data for the 2-dye $0.85\times R_0$. Data shows the Cy3 donor alone, the Cy3-Cy5 donor acceptor pair and the Cy5 acceptor only plots. (**A**) 8-way (**B**) 4-way (**C**) 2-way (**D**) 1-way.

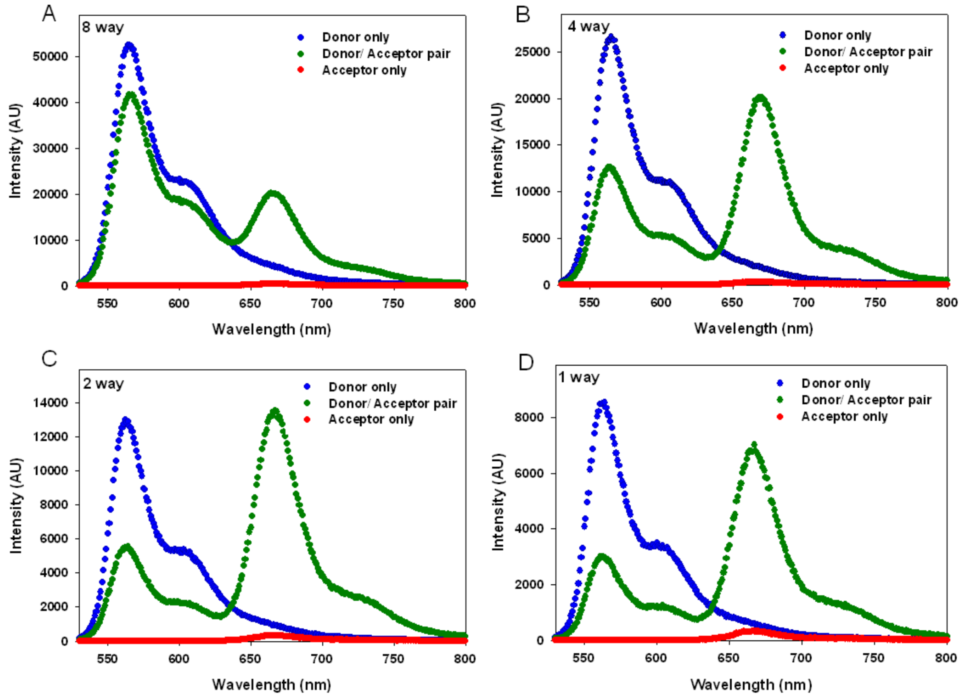

Supplementary Figure 6. Raw data for the 2-dye $0.75\times R_0$. Data shows the Cy3 donor alone, the Cy3-Cy5 donor acceptor pair and the Cy5 acceptor only plots. (**A**) 8-way (**B**) 4-way (**C**) 2-way (**D**) 1-way.

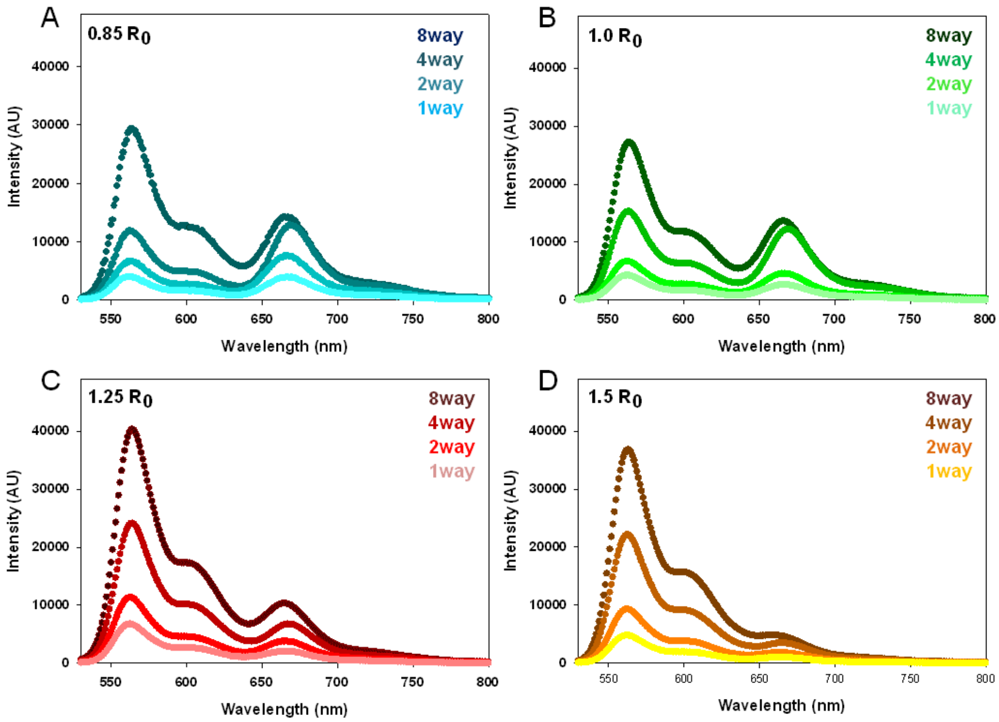

Supplementary Figure 7. 2-dye data all normalized to the 0.75×R_0 Cy3 direct excitation peak. Each separate plot indicates a different spacing (**A**) 0.85×R_0 (**B**) 1.0×R_0 (**C**) 1.25×R_0 (**D**) 1.5×R_0. Within each plot the darkest curve indicates the 8-way structure going down to the 1-way structure which is the lightest.

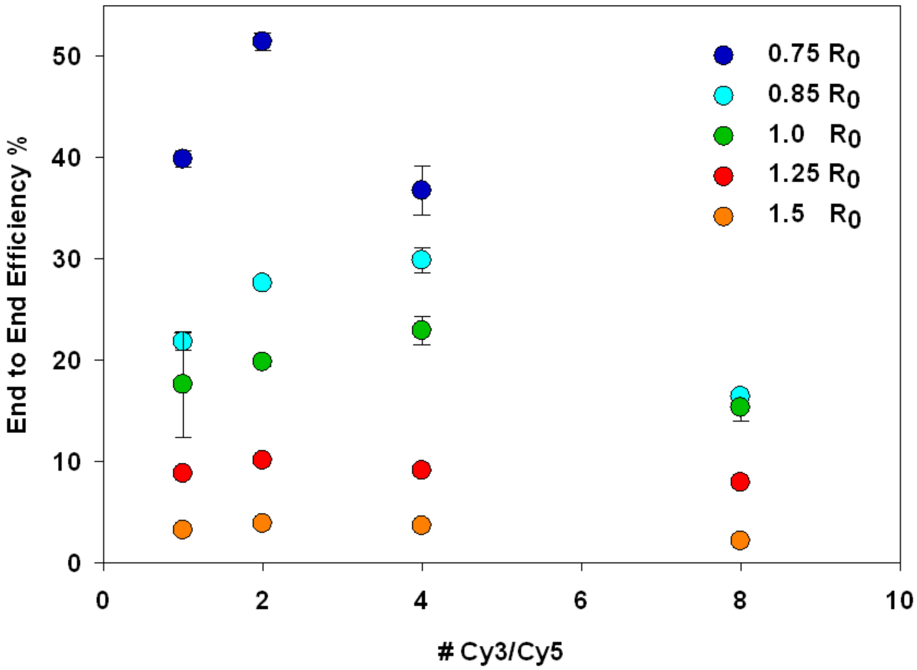

Supplementary Figure 8. End-to-end efficiency of the 2-dye constructs. Data are plotted against the ratio of Cy3 donors to Cy5 acceptors.

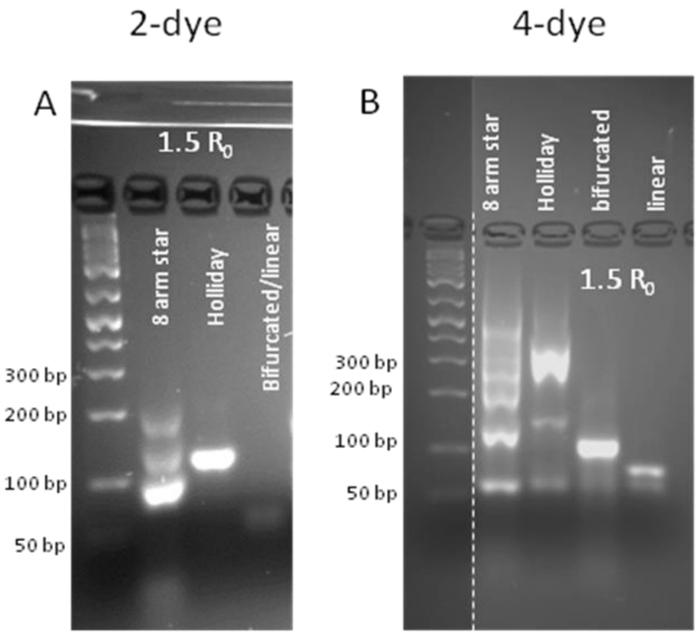

Supplementary Figure 9. Representative gel electrophorograms. (**A**) 1.5×R_0 2-dye structures and (**B**) 1.5×R_0 4-dye structures.

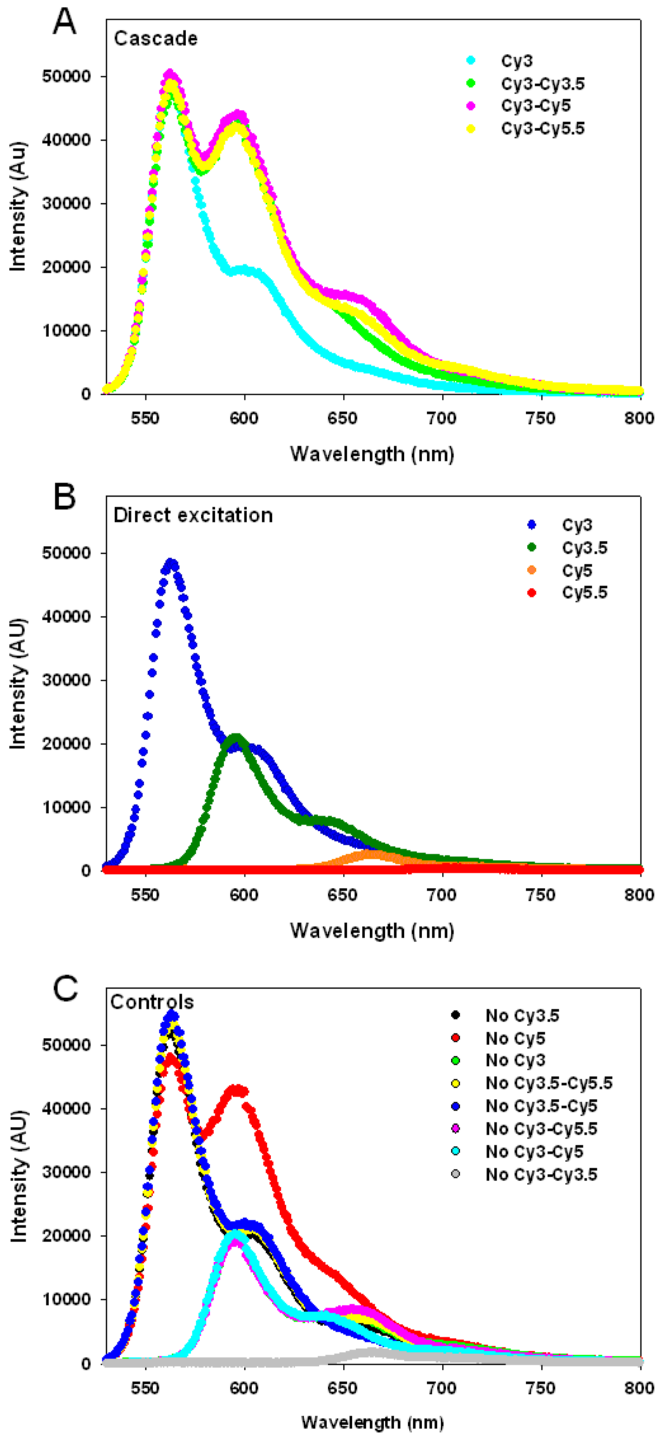

Supplementary Figure 10. Raw data for the 4-dye 1-way 1.5×R_0. (**A**) The cascade data showing the FRET progression through sequential addition of each fluorophore. (**B**) Direct excitation of each of the fluorophore assembled alone, onto the DNA construct. (**C**) Control data, where one or two dyes are missing in the 4-dye sequence.

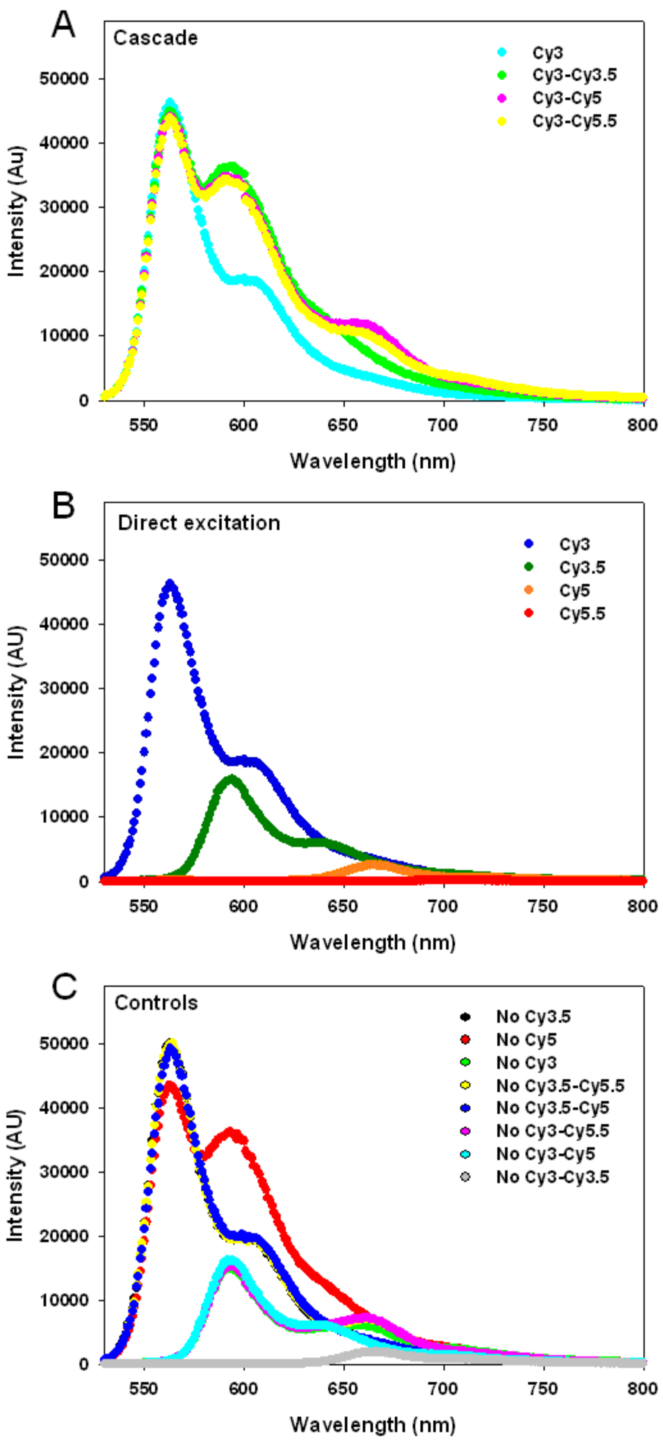

Supplementary Figure 11. Raw data for the 4-dye 2-way 1.5×R_0. (**A**) The cascade data showing the FRET progression through sequential addition of each fluorophore. (**B**) Direct excitation of each of the fluorophore assembled alone, onto the DNA construct. (**C**) Control data, where one or two dyes are missing in the 4-dye sequence.

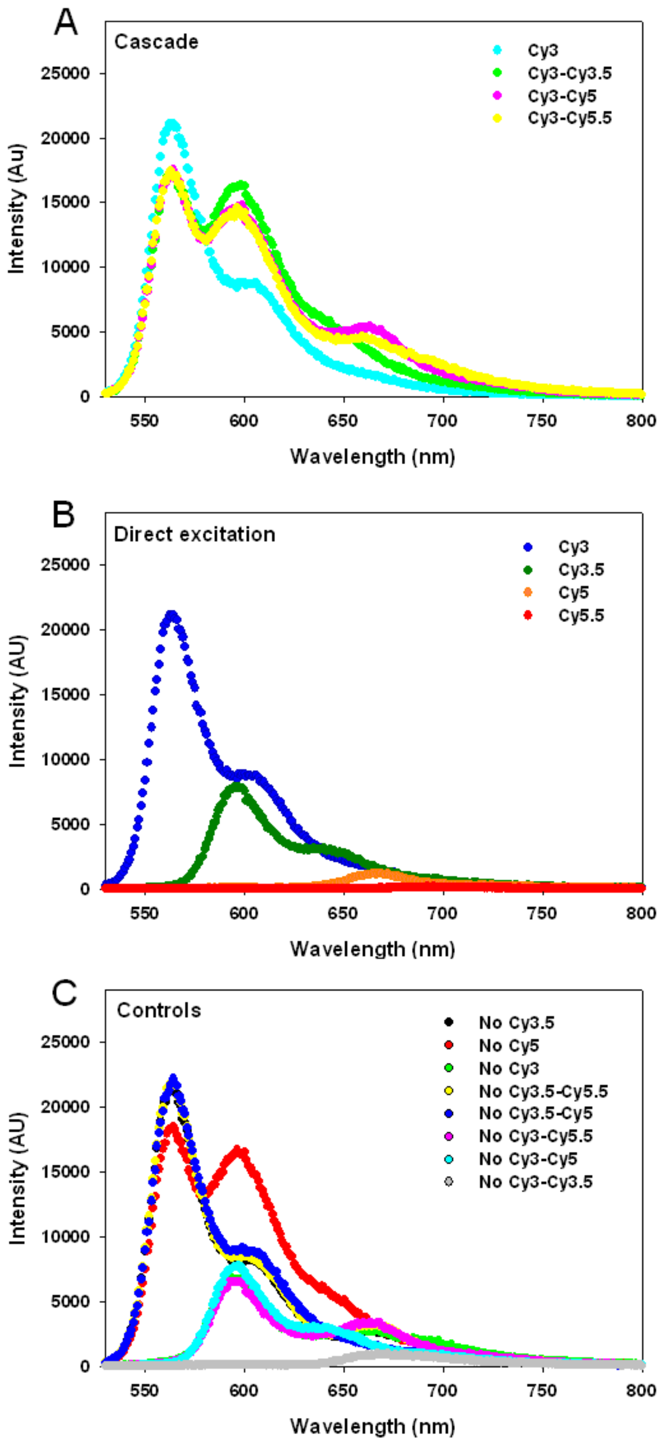

Supplementary Figure 12. Raw data for the 4-dye 4-way 1.5×R_0. (**A**) The cascade data showing the FRET progression through sequential addition of each fluorophore. (**B**) Direct excitation of each of the fluorophore assembled alone, onto the DNA construct. (**C**) Control data, where one or two dyes are missing in the 4-dye sequence.

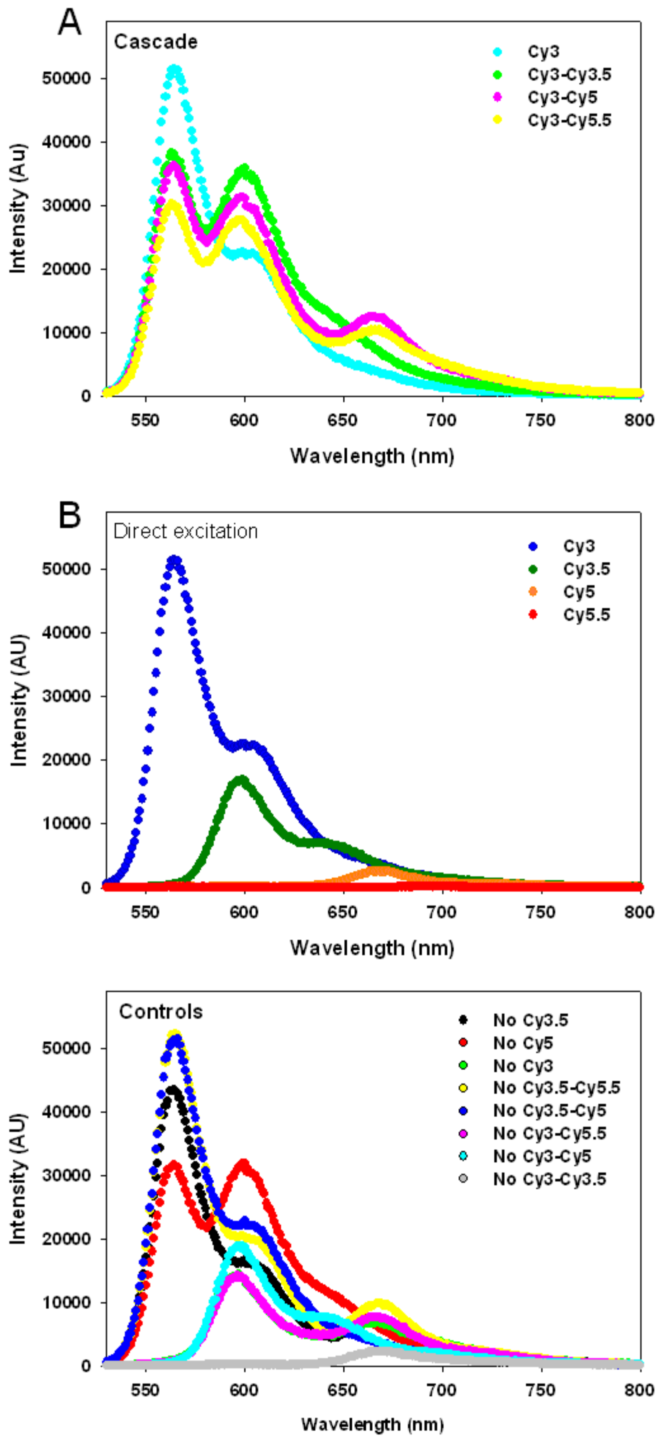

Supplementary Figure 13. Raw data for the 4-dye 8-way 1.5×R_0. (**A**) The cascade data showing the FRET progression through sequential addition of each fluorophore. (**B**) Direct excitation of each of the fluorophore assembled alone, onto the DNA construct. (**C**) Control data, where one or two dyes are missing in the 4-dye sequence.

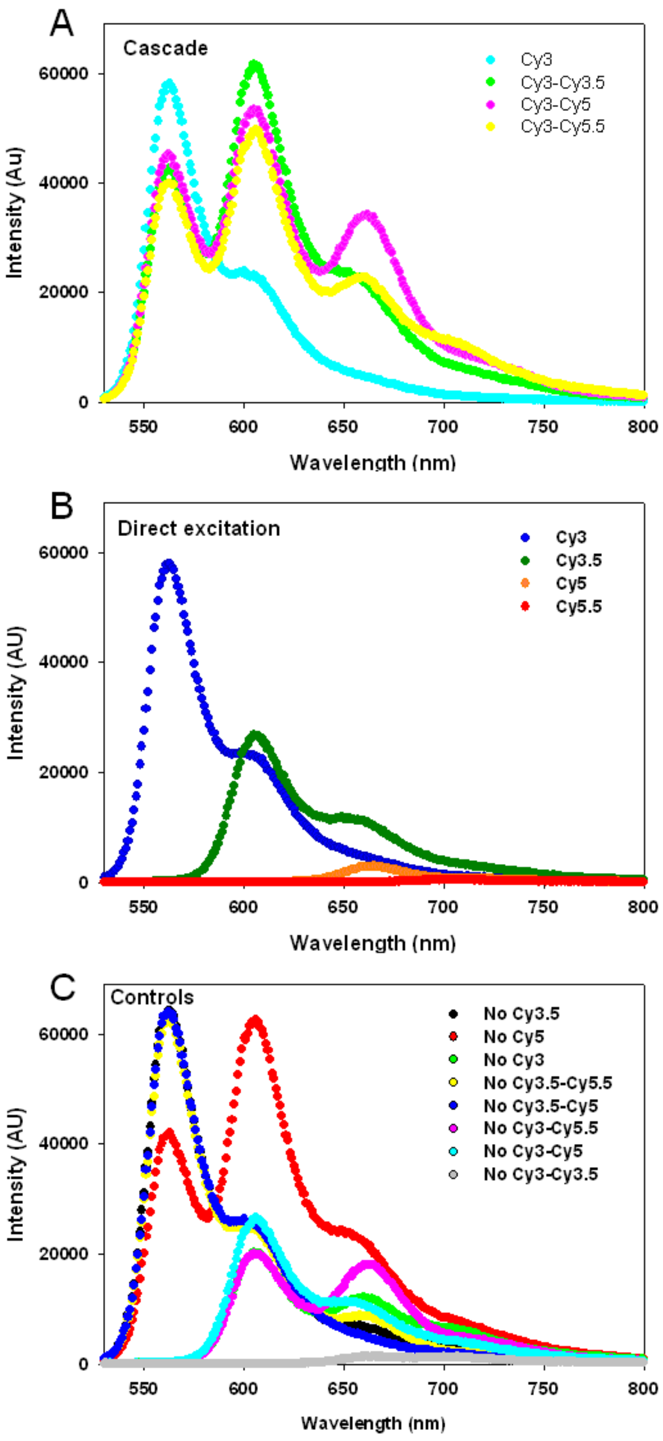

Supplementary Figure 14. Raw data for the 4-dye 1-way 1.0×*R*$_0$. (**A**) The cascade data showing the FRET progression through sequential addition of each fluorophore. (**B**) Direct excitation of each of the fluorophore assembled alone, onto the DNA construct. (**C**) Control data, where one or two dyes are missing in the 4-dye sequence.

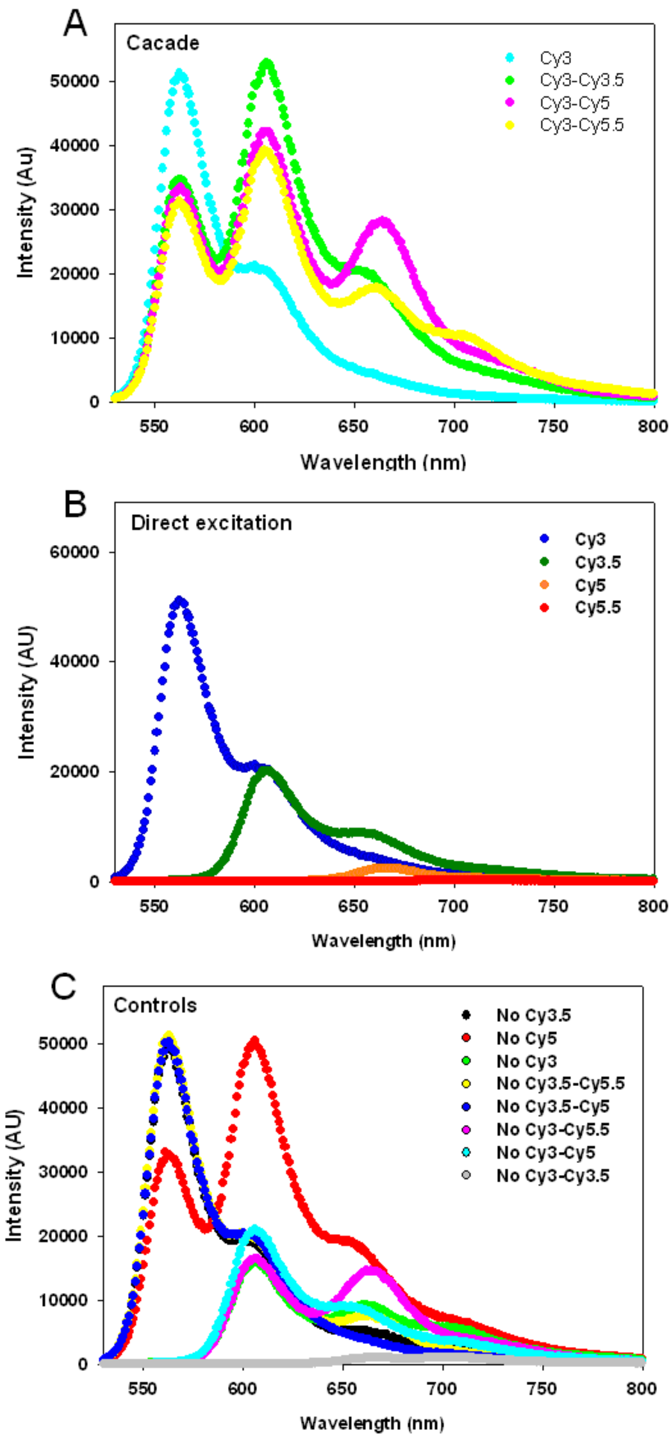

Supplementary Figure 15. Raw data for the 4-dye 2-way 1.0×R_0. (**A**) The cascade data showing the FRET progression through sequential addition of each fluorophore. (**B**) Direct excitation of each of the fluorophore assembled alone, onto the DNA construct. (**C**) Control data, where one or two dyes are missing in the 4-dye sequence.

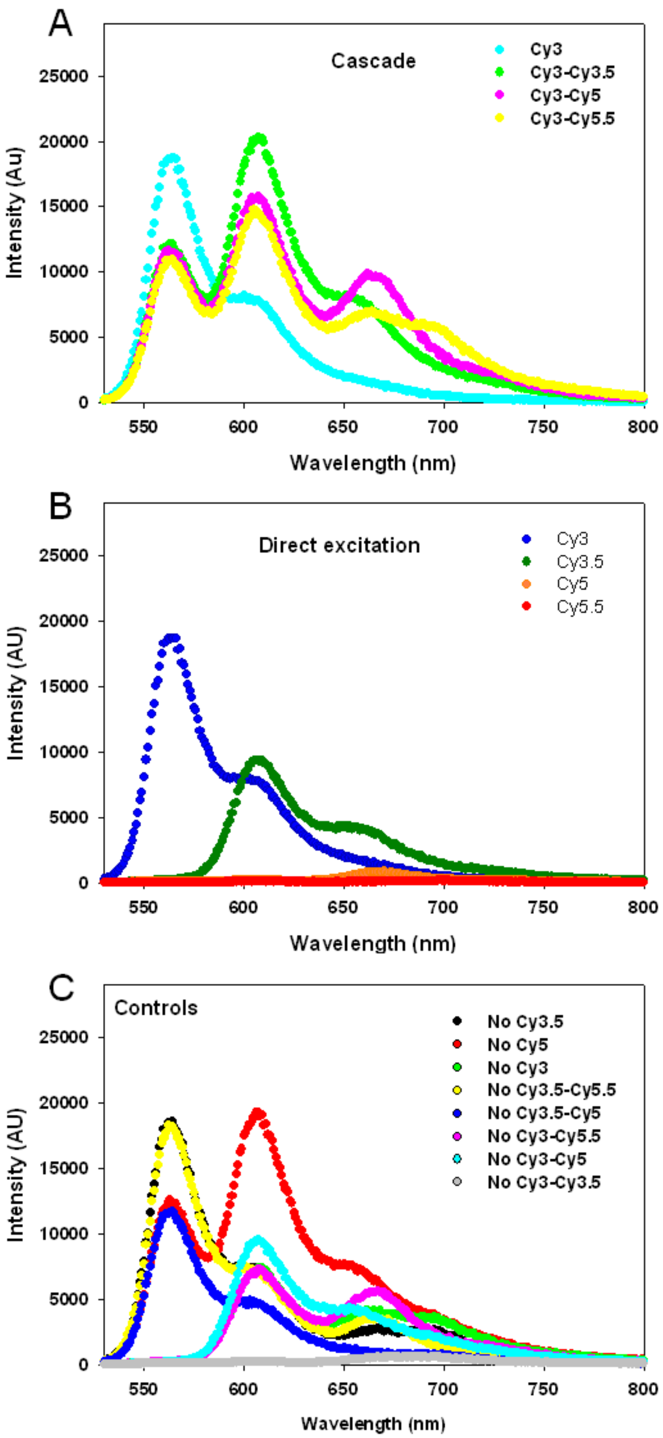

Supplementary Figure 16. Raw data for the 4-dye 4-way 1.0×R_0. (**A**) The cascade data showing the FRET progression through sequential addition of each fluorophore. (**B**) Direct excitation of each of the fluorophore assembled alone, onto the DNA construct. (**C**) Control data, where one or two dyes are missing in the 4-dye sequence.

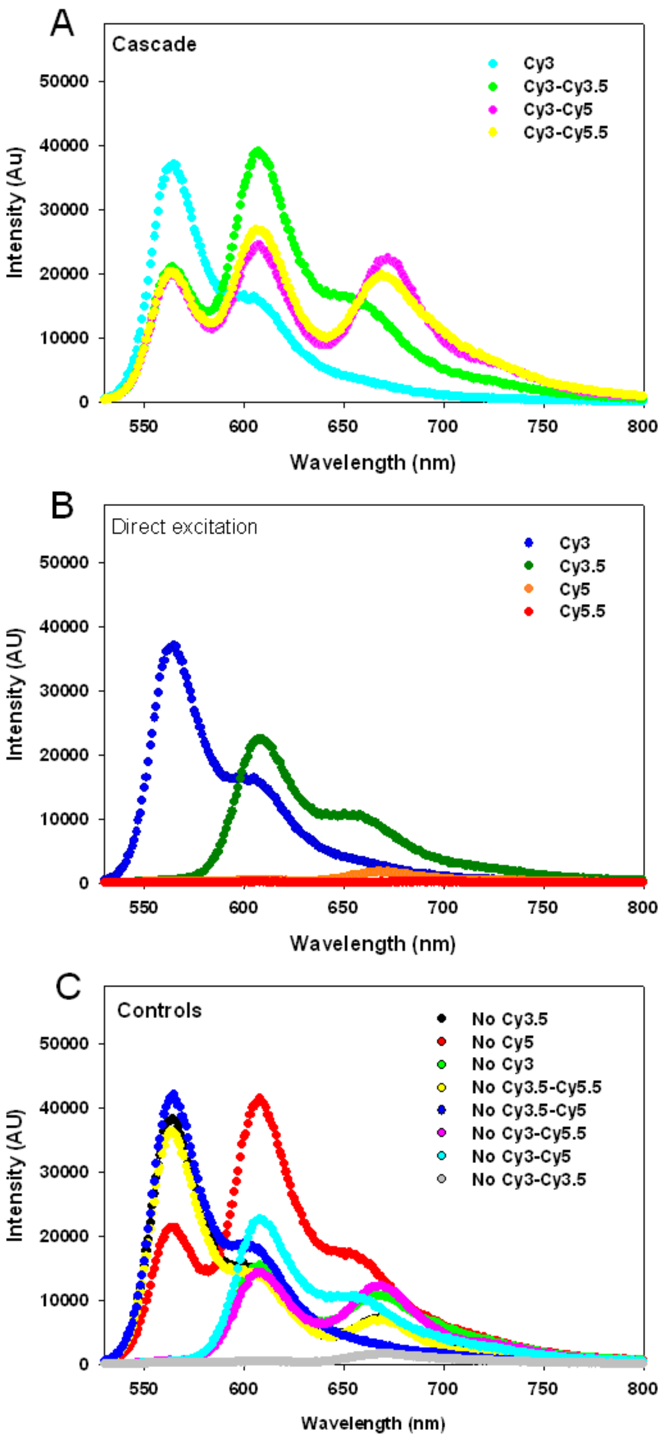

Supplementary Figure 17. Raw data for the 4-dye 8-way 1.0×R_0. (**A**) The cascade data showing the FRET progression through sequential addition of each fluorophore. (**B**) Direct excitation of each of the fluorophore assembled alone, onto the DNA construct. (**C**) Control data, where one or two dyes are missing in the 4-dye sequence.

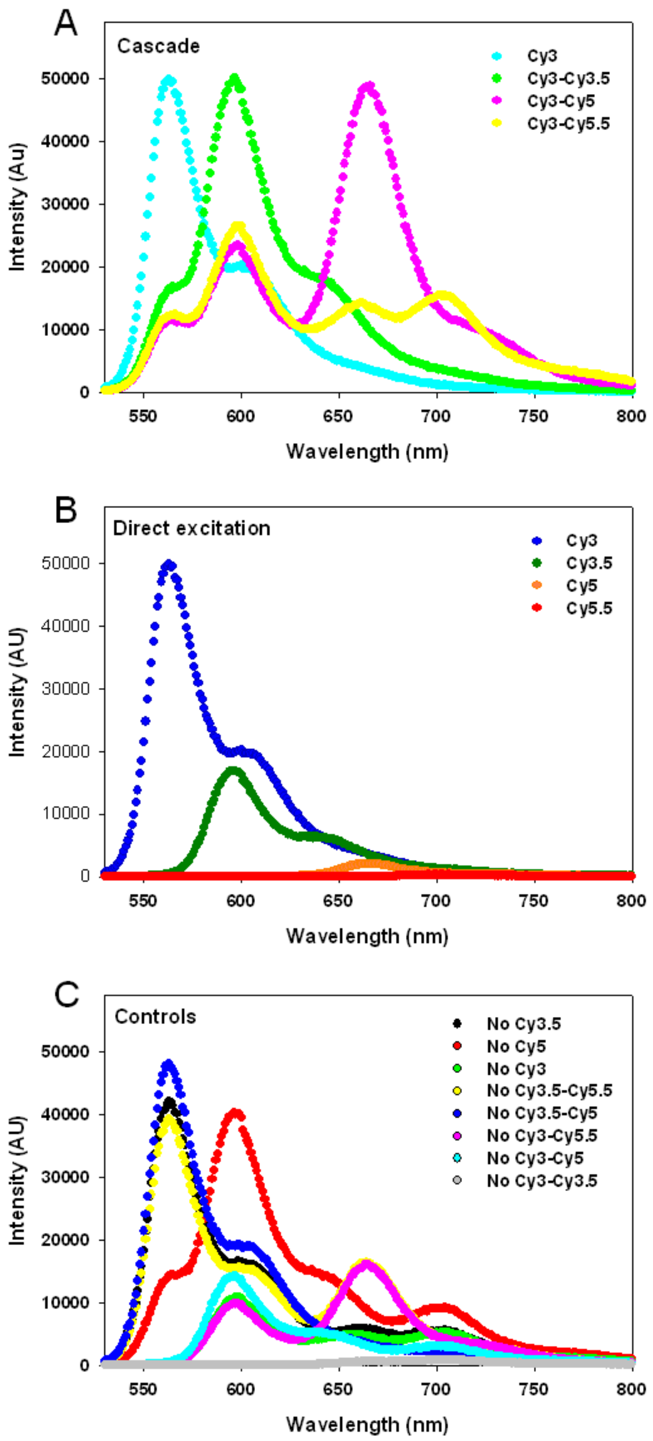

Supplementary Figure 18. Raw data for the 4-dye 1-way 0.5×R_0. (**A**) The cascade data showing the FRET progression through sequential addition of each fluorophore. (**B**) Direct excitation of each of the fluorophore assembled alone, onto the DNA construct. (**C**) Control data, where one or two dyes are missing in the 4-dye sequence.

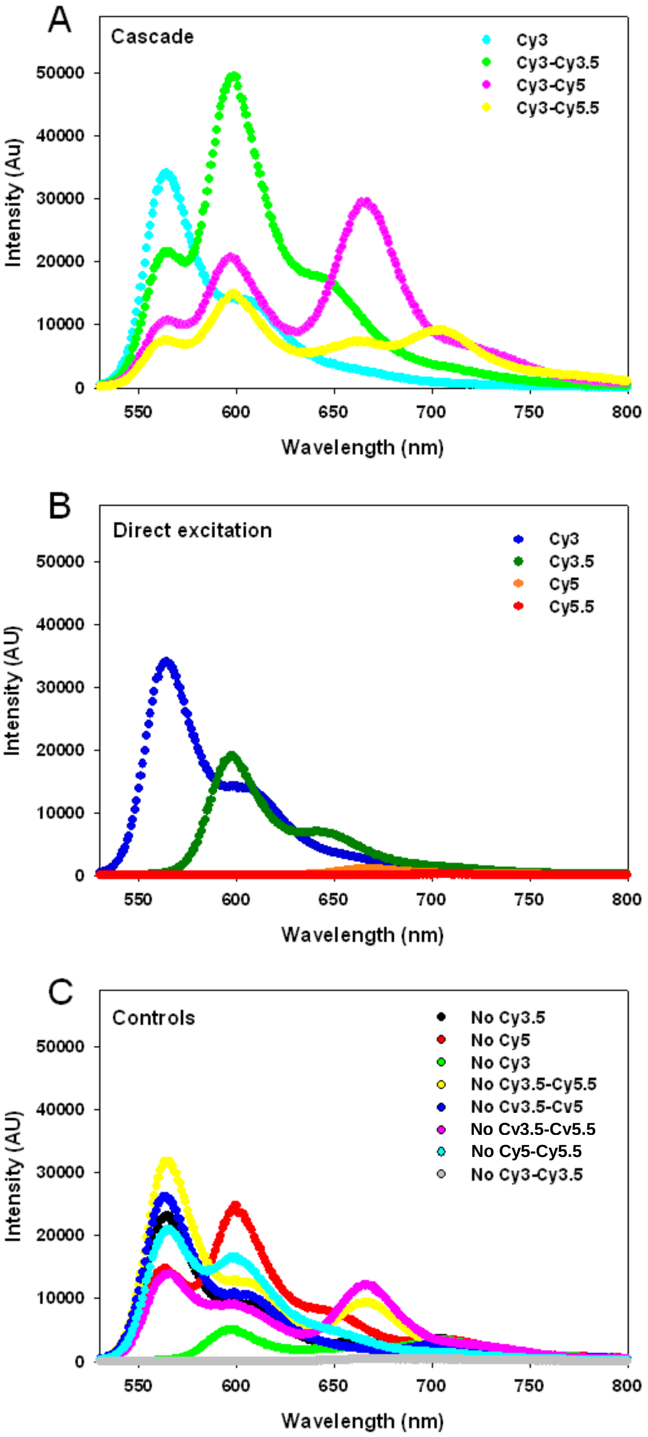

Supplementary Figure 19. Raw data for the 4-dye 2-way 0.5×R_0. (**A**) The cascade data showing the FRET progression through sequential addition of each fluorophore. (**B**) Direct excitation of each of the fluorophore assembled alone, onto the DNA construct. (**C**) Control data, where one or two dyes are missing in the 4-dye sequence.

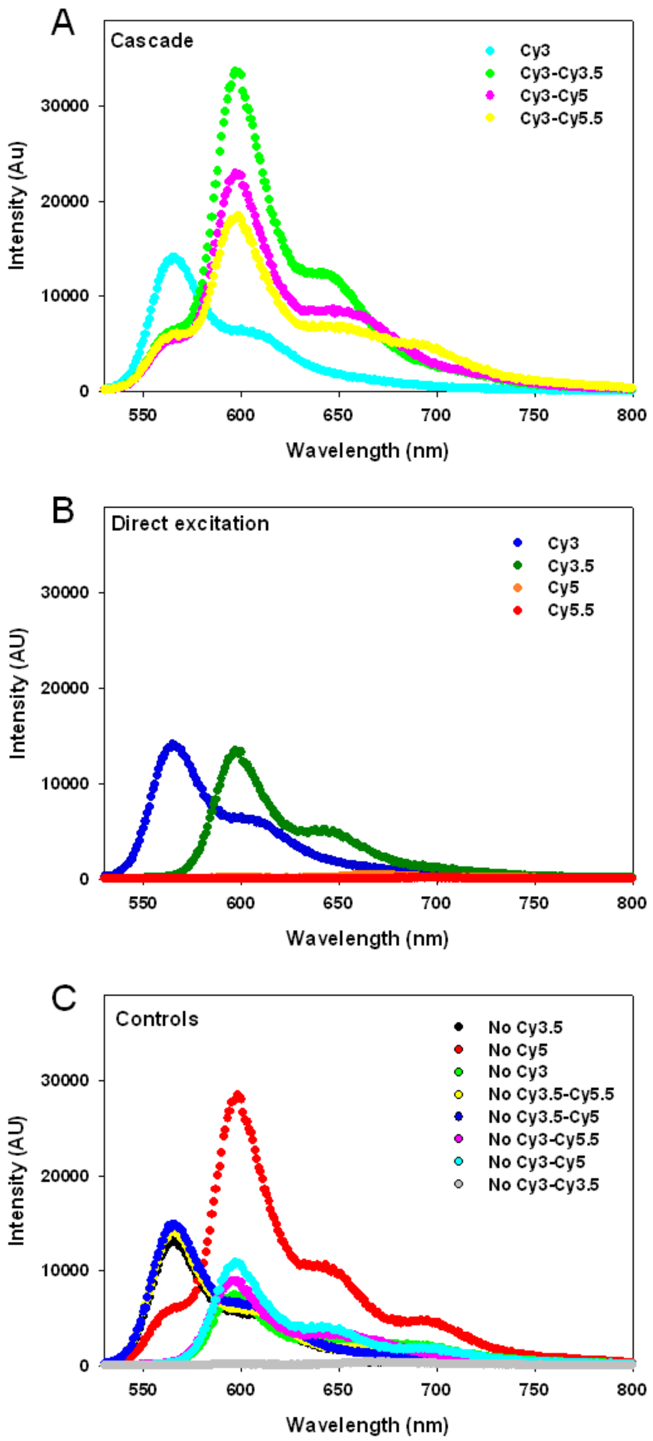

Supplementary Figure 20. Raw data for the 4-dye 4-way 0.5×R_0. (**A**) The cascade data showing the FRET progression through sequential addition of each fluorophore. (**B**) Direct excitation of each of the fluorophore assembled alone, onto the DNA construct. (**C**) Control data, where one or two dyes are missing in the 4-dye sequence.

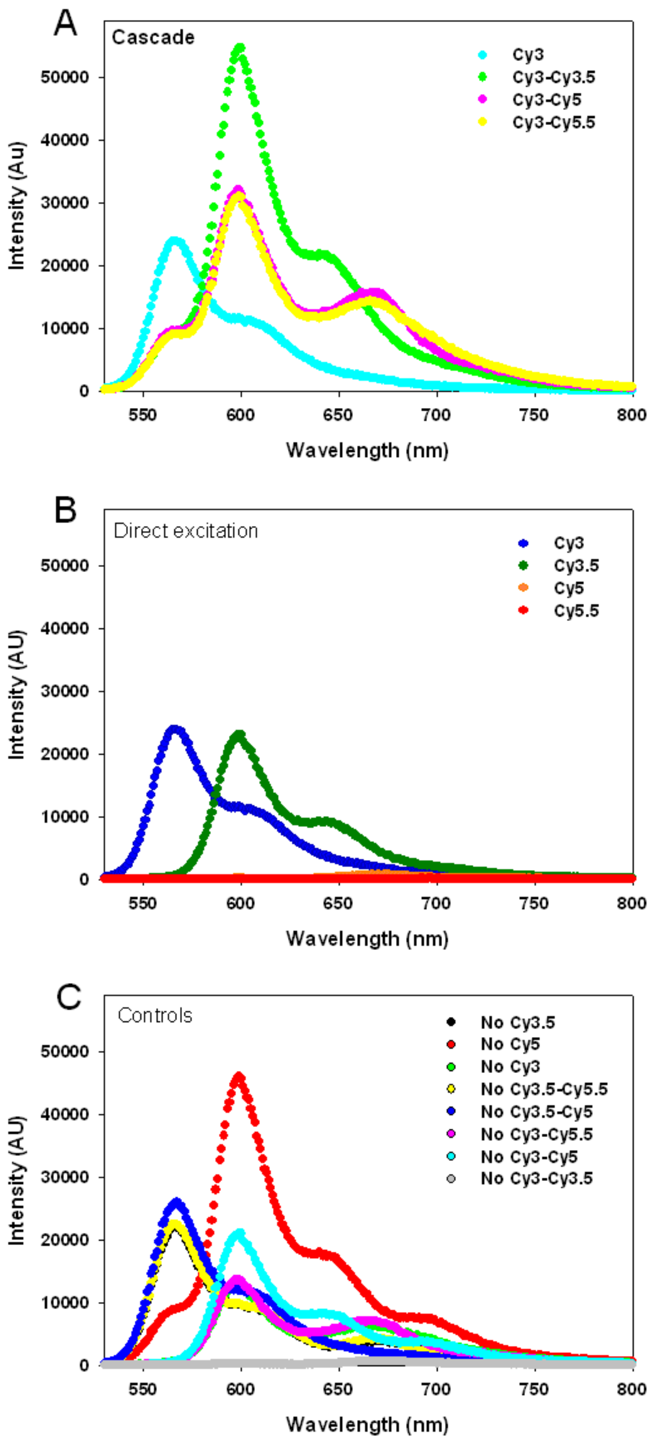

Supplementary Figure 21. Raw data for the 4-dye 8-way 0.5×R_0. (A) The cascade data showing the FRET progression through sequential addition of each fluorophore. (B) Direct excitation of each of the fluorophore assembled alone, onto the DNA construct. (C) Control data, where one or two dyes are missing in the 4-dye sequence.

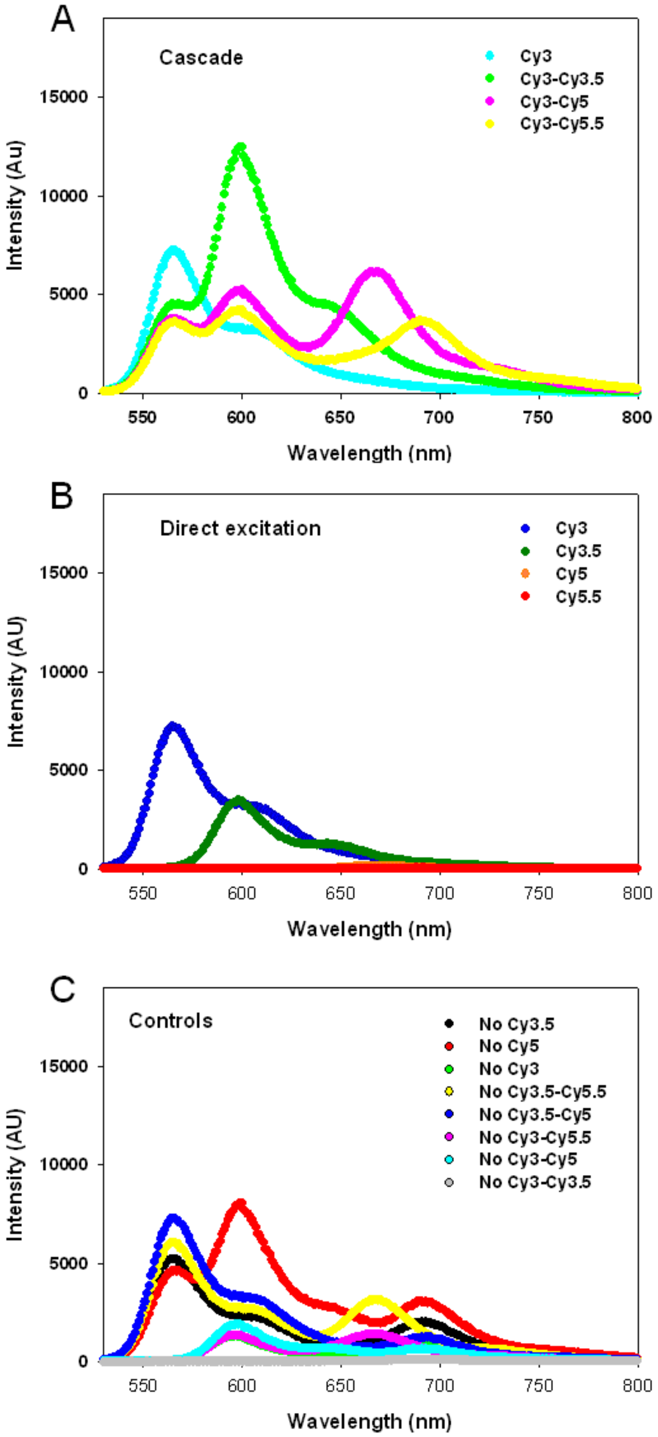

Supplementary Figure 22. Raw data for the 4-dye, 0.5×R_0 2:1 dendrimer. (**A**) The cascade data showing the FRET progression through sequential addition of each fluorophore. (**B**) Direct excitation of each of the fluorophore assembled alone, onto the DNA construct. (**C**) Control data, where one or two dyes are missing in the 4-dye sequence.

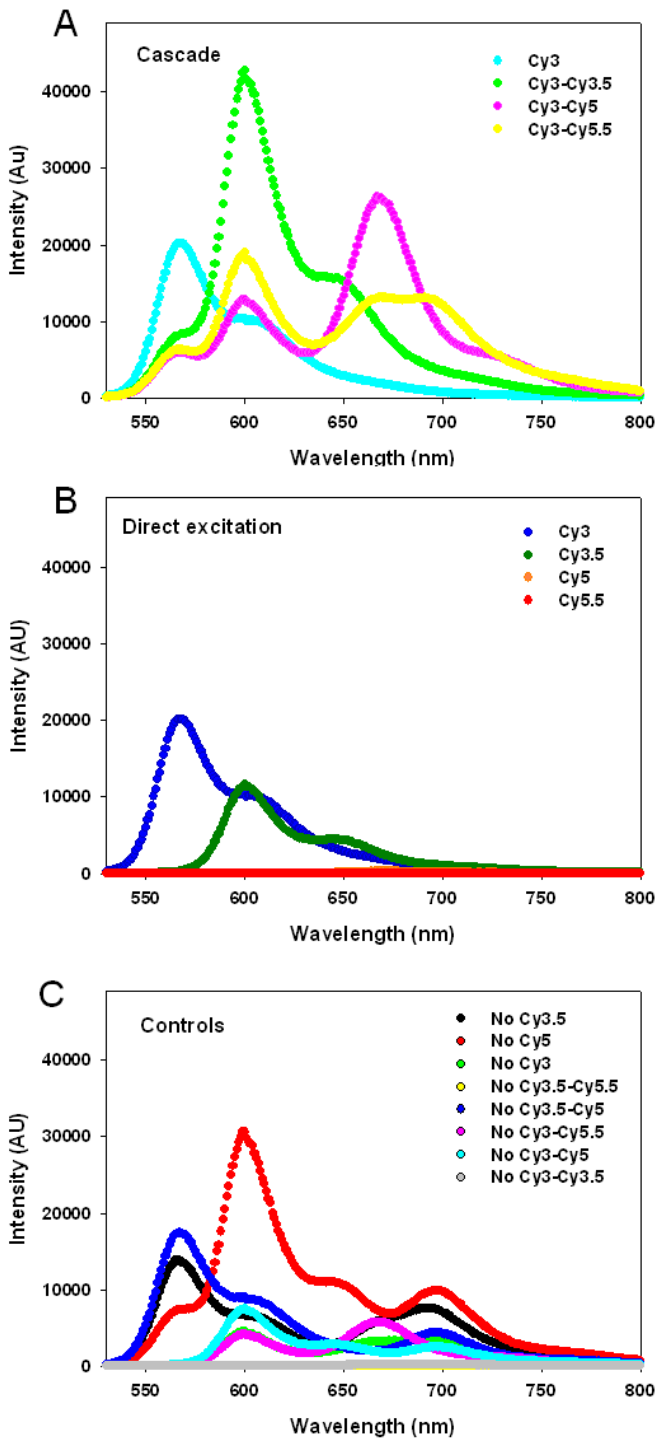

Supplementary Figure 23. Raw data for the 4-dye, 0.5×R_0 3:1 dendrimer. (**A**) The cascade data showing the FRET progression through sequential addition of each fluorophore. (**B**) Direct excitation of each of the fluorophore assembled alone, onto the DNA construct. (**C**) Control data, where one or two dyes are missing in the 4-dye sequence.

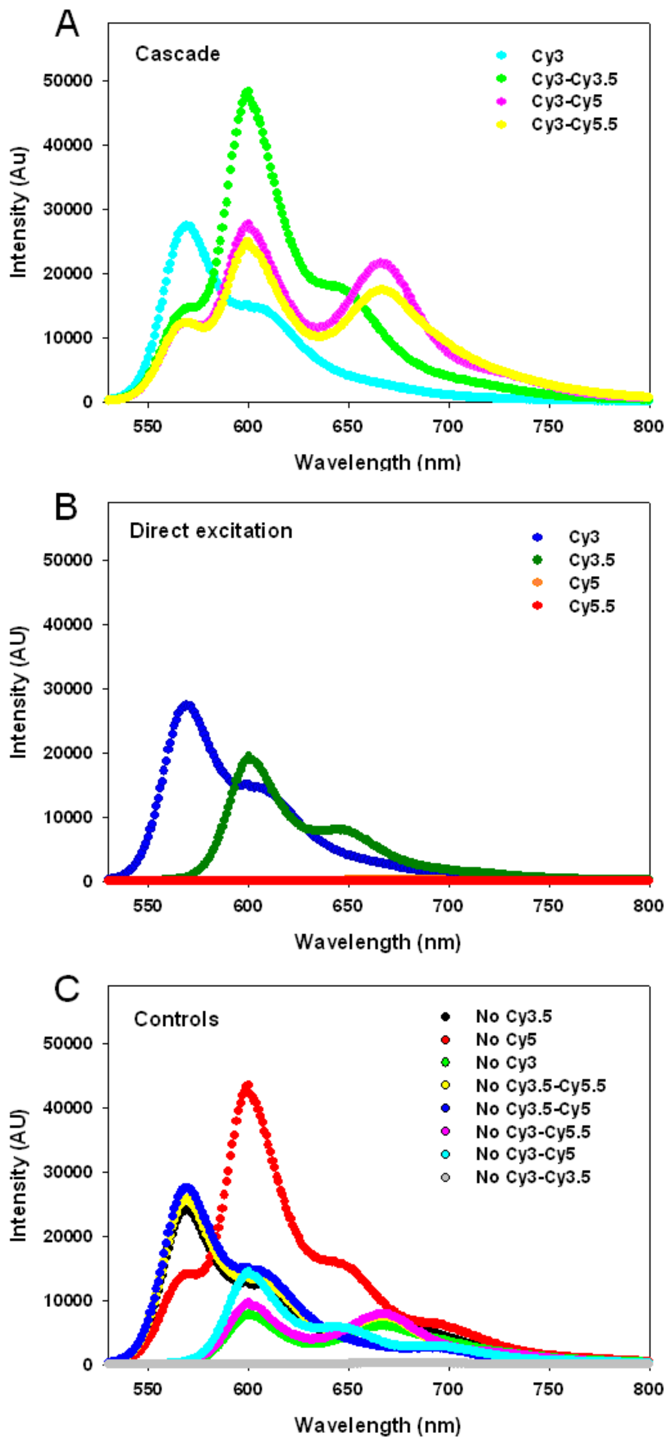

Supplementary Figure 24. Raw data for the 4-dye, 0.5×R_0 4:1 dendrimer. (**A**) The cascade data showing the FRET progression through sequential addition of each fluorophore. (**B**) Direct excitation of each of the fluorophore assembled alone, onto the DNA construct. (**C**) Control data, where one or two dyes are missing in the 4-dye sequence.

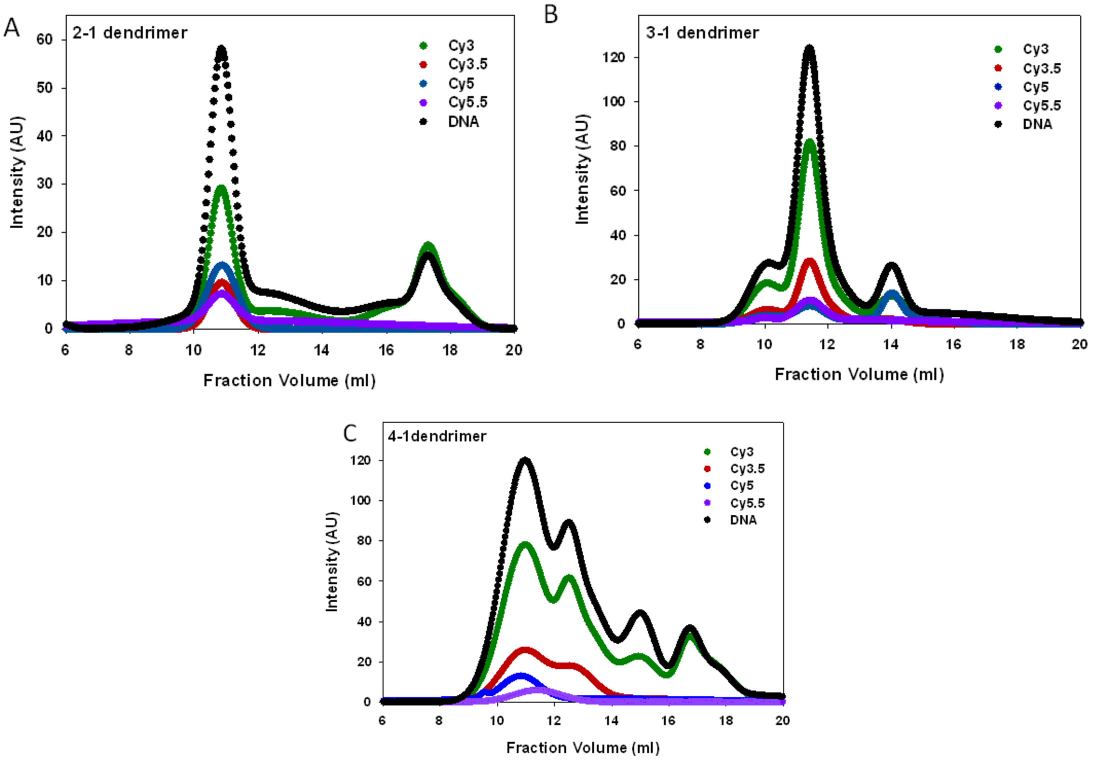

Supplementary Figure 25. Representative FPLC analysis of the dendrimer structures. (**A**) 2:1 dendrimer, (**B**) 3:1 dendrimer, (**C**) 4:1 dendrimer. For the 4-dye dendrimers the intensity of the dye absorption is shown, which provides information about the contents of each sub peak.

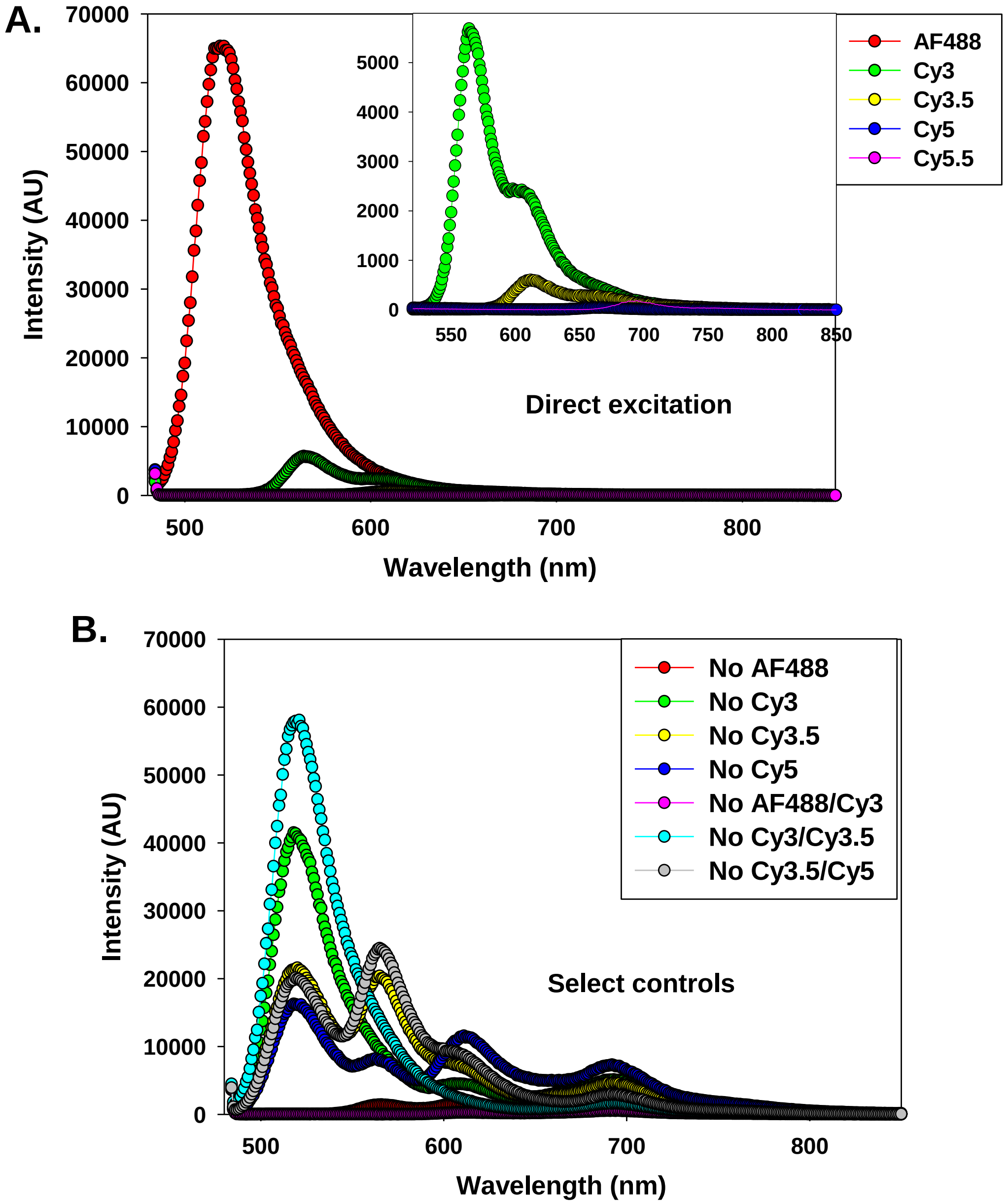

Supplementary Figure 26. Raw data for the 5-dye, 0.5×R_0 2:1 dendrimer incorporating Cy5 at the 4th step. (**A**) Direct excitation of each of the fluorophores assembled alone, onto the DNA construct. (**B**) Select control data, where one or two dyes are missing in the 5-dye sequence.

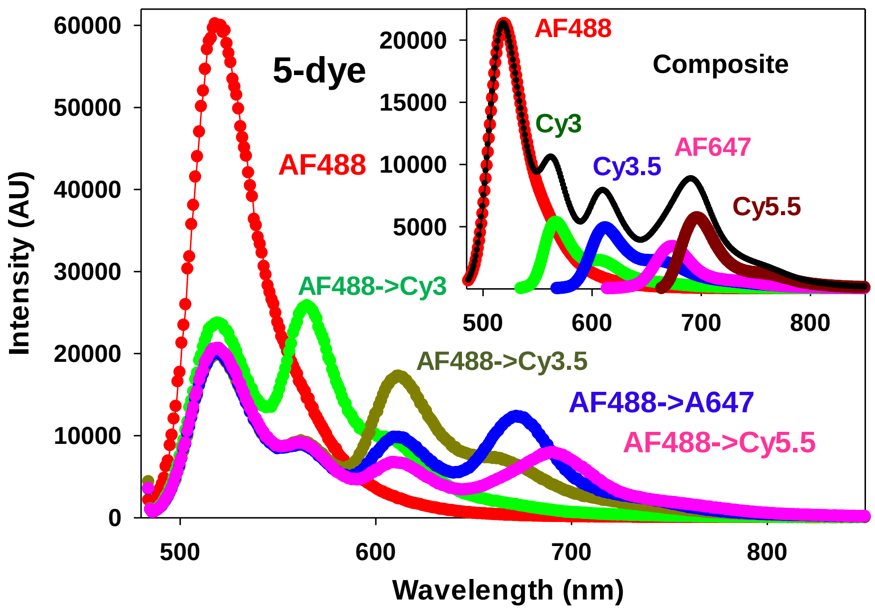

Supplementary Figure 27. Data for the 5-dye, 0.5×R_0 AF488$_{16}$→Cy3$_8$→Cy3.5$_4$→AF647$_2$ →Cy5.5$_1$ 2:1 dendrimer incorporating AF647 at the 4th step. Constructs were excited at 465 nm. Inset is the deconvolved components for each dye in the final AF488 →Cy5.5 construct superimposed by that of the composite spectra.

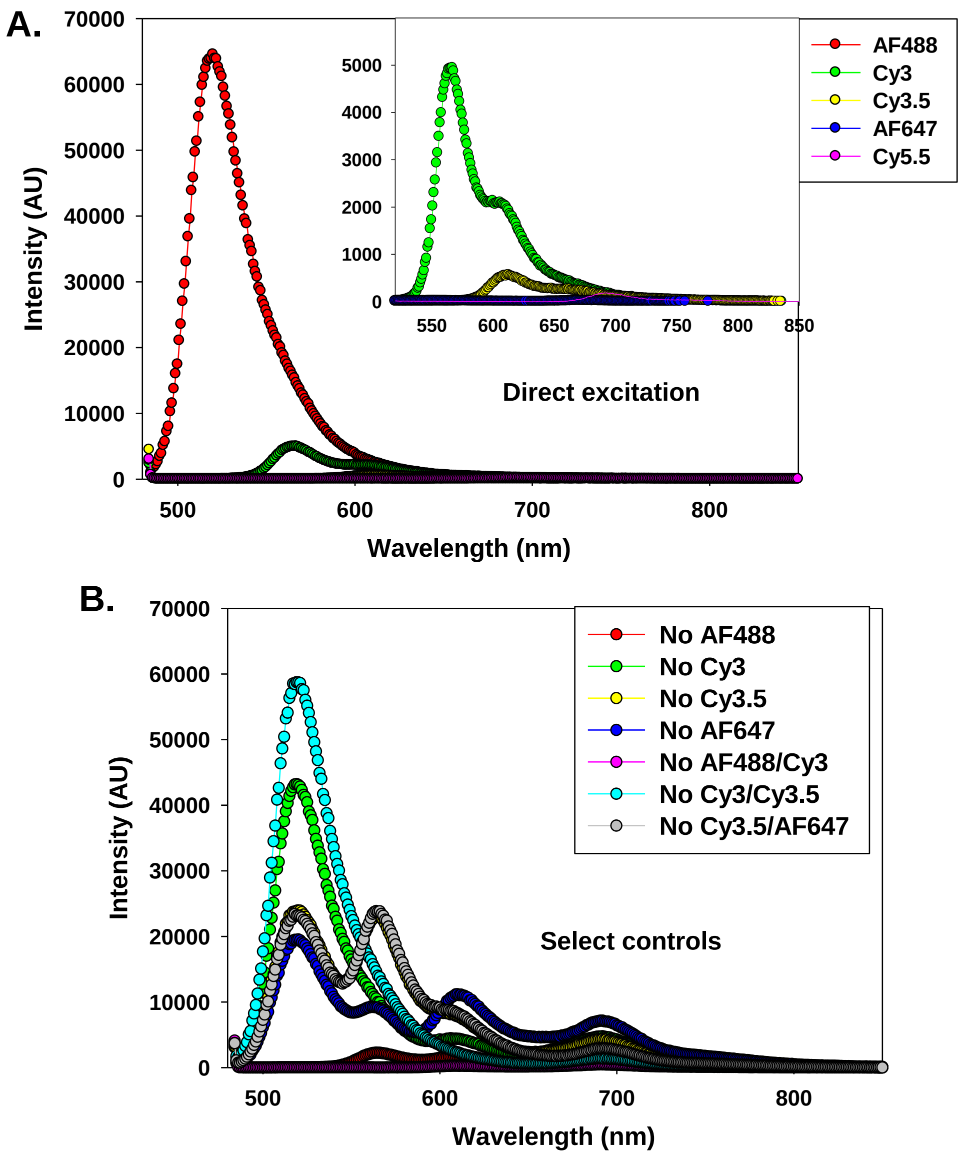

Supplementary Figure 28. Raw data for the 5-dye, 0.5×R_0 2:1 dendrimer incorporating AF647 at the 4th step. (**A**) Direct excitation of each of the fluorophores assembled alone, onto the DNA construct. (**B**) Select control data, where one or two dyes are missing in the 5-dye sequence.

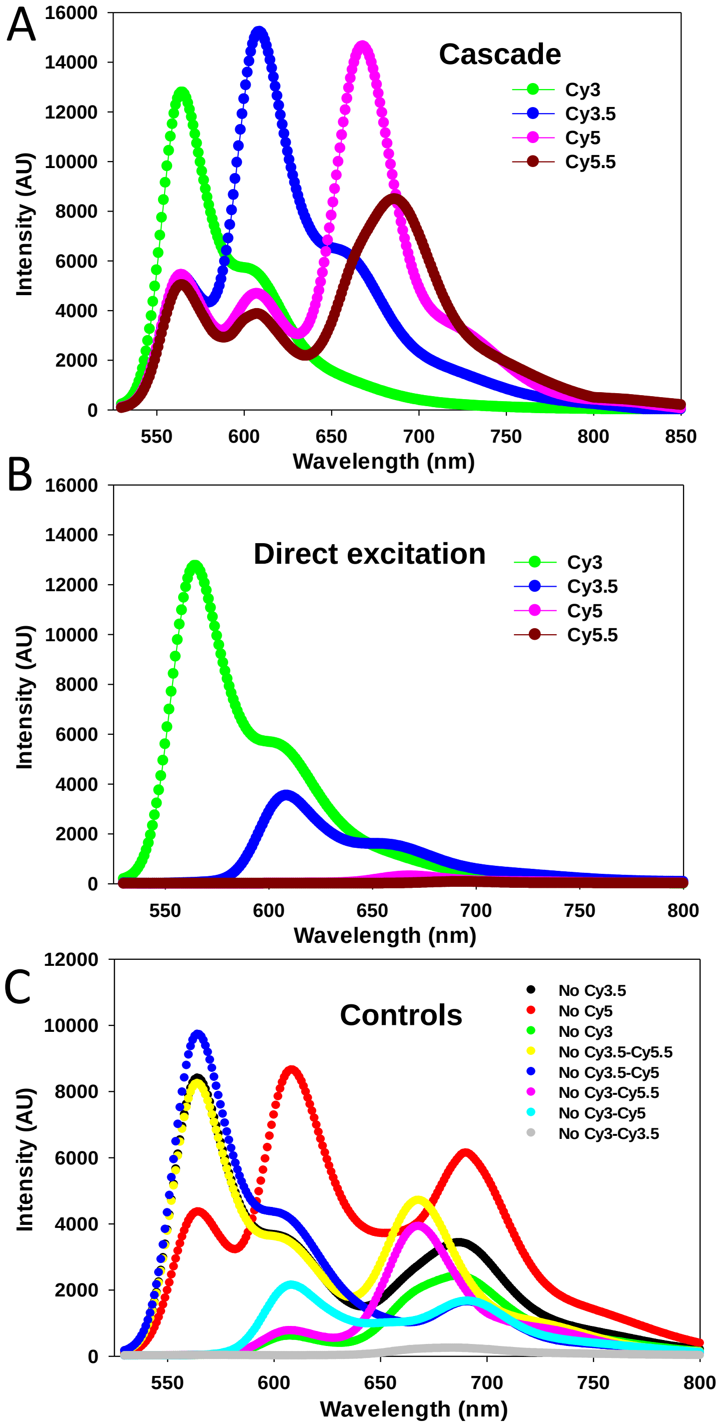

Supplementary Figure 29. Raw data for the 4-dye, 0.5×R_0 2:1 dendrimer incorporating a Cy3.5 phosphoramidite dye at the 2nd step. (**A**) The cascade data showing the FRET progression through sequential addition of each fluorophore. (**B**) Direct excitation of each of the fluorophore assembled alone, onto the DNA construct. (**C**) Control data, where one or two dyes are missing in the 4-dye sequence.

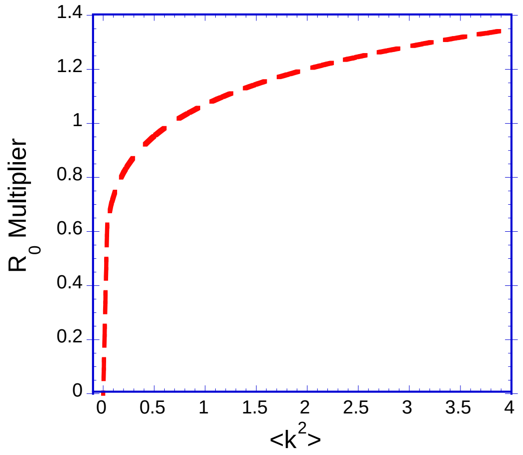

Supplementary Figure 30. The effect of dipole orientation on R_0.

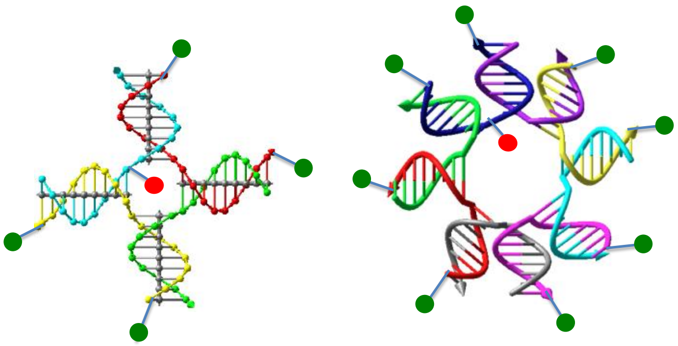

Supplementary Figure 31. Representation of 2-dye system. Donor (green) and acceptor (red) positions are shown in the 4-way (left) and 8-way (right) dsDNA junction.

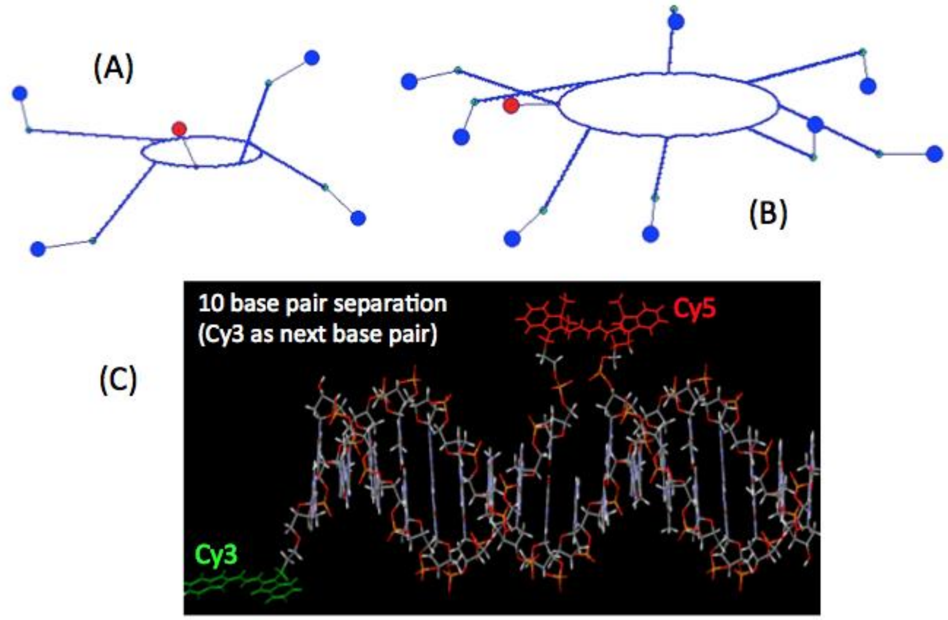

Supplementary Figure 32. Model representations of 2-dye constructs and dsDNA. Depictions of the (**A**) 4-arm and (**B**) 8-arm constructs with the circular representations of the central opening in these structures as discussed in the text. (**C**) Model of dsDNA showing the molecular attachment of Cy5 and Cy3.

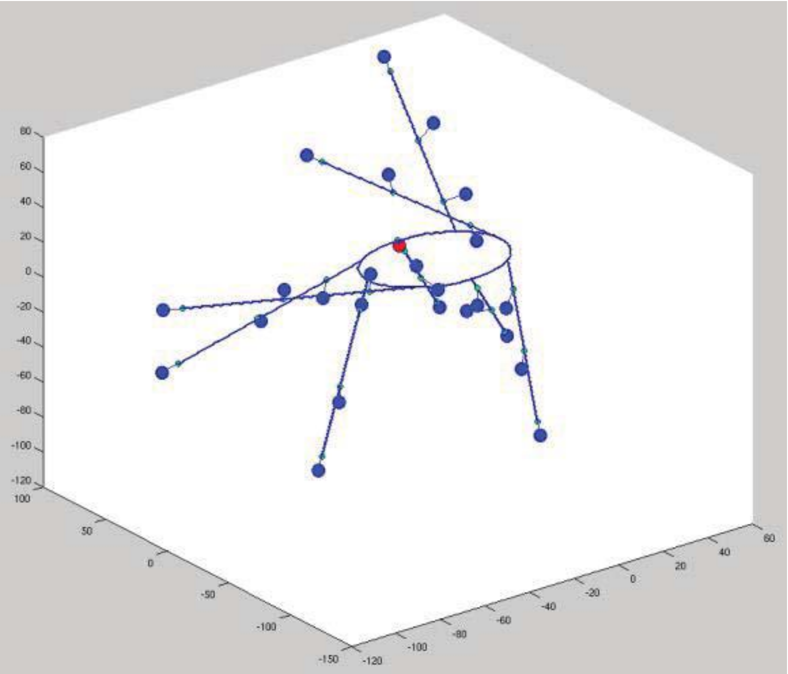

Supplementary Figure 33. **One member of the ensemble for the 4-dye 8-arm star with random arm and linker angles.** The dyes are the blue dots, with the red dot being the Cy5.5 dye.

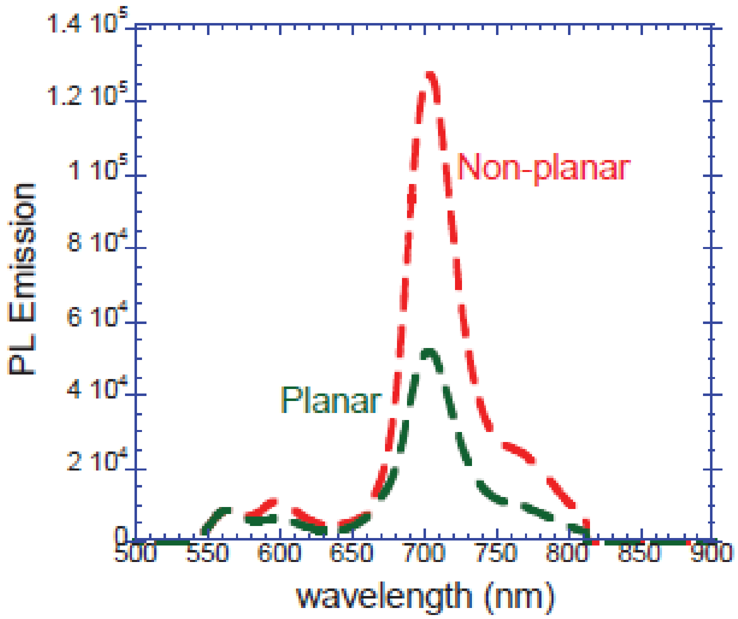

Supplementary Figure 34. Comparison of simulated PL spectra for planar and non-planar geometries. Plots are for the 4-dye, 8-arm stars with 100% formation efficiency and comparing results when the simulated ensemble incorporates random angles (as in Supplementary Figure 32) as opposed to all structures assumed planar.

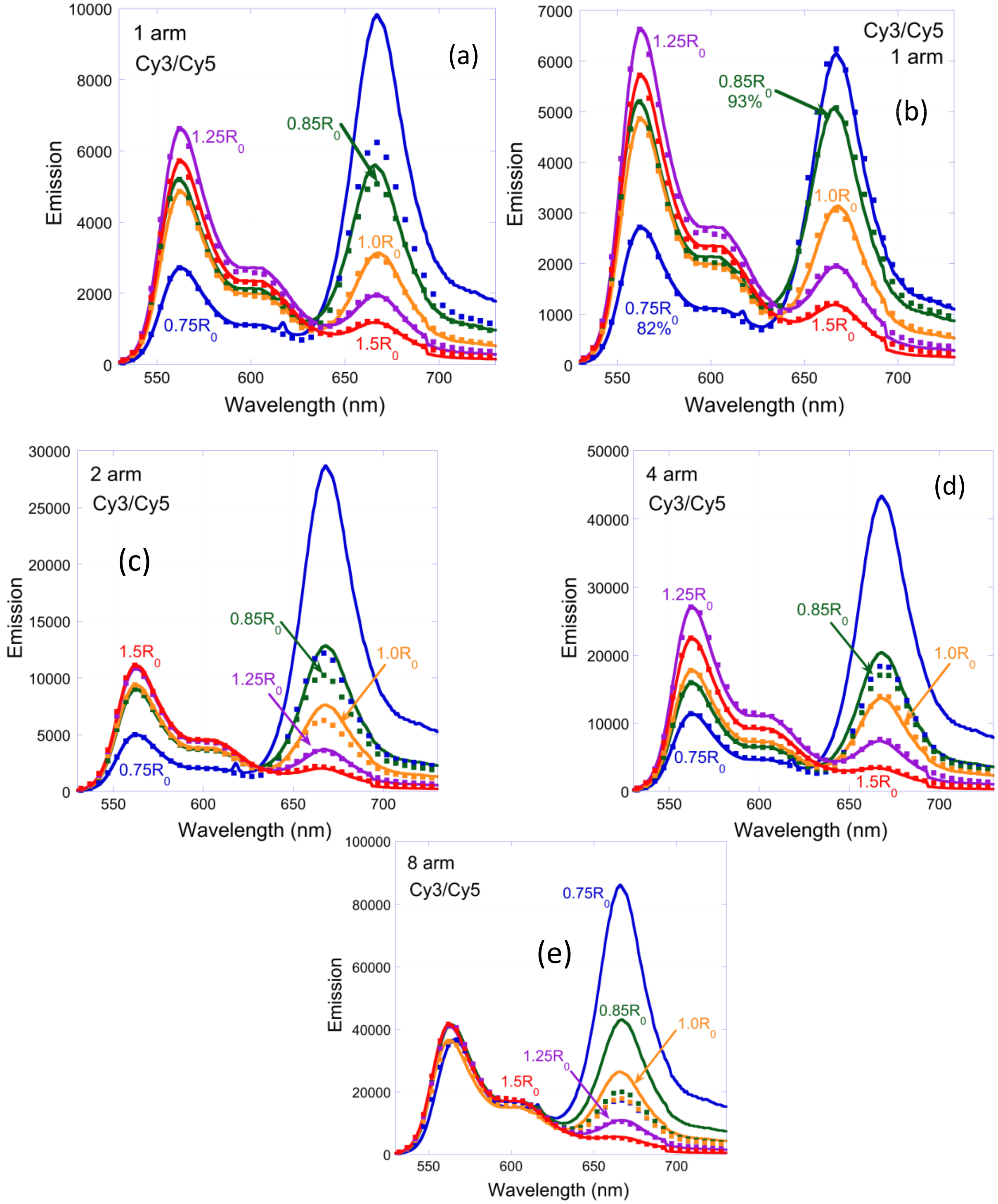

Supplementary Figure 35. Comparisons of experimental and ideal simulated spectra. For (**a**) 1-arm, (**c**) 2-arm, (**d**) 4-arm and (**e**) 8-arm two-dye stars labeled with Cy3 and Cy5 dyes and showing overall good agreement with only small discrepancies especially as the dye spacing gets smaller. In (**b**) is a plot corresponding to (**a**) that shows that adjustments in the yield, in this case small, can lead to good agreement between simulation and experiment for all dye spacing's.

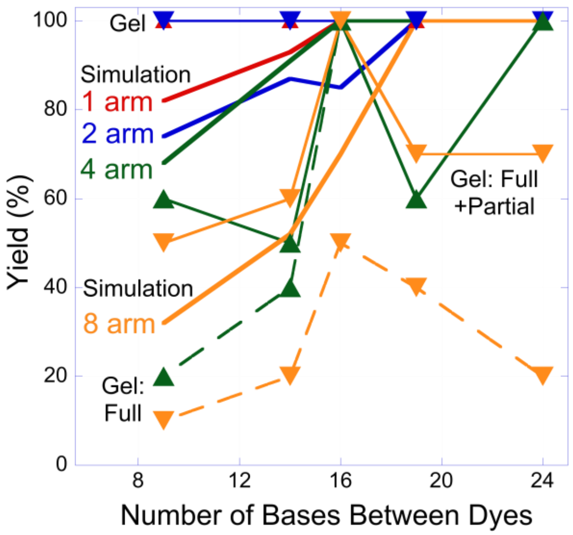

Supplementary Figure 36. Plot (solid lines without symbols) of the simulation-derived yield reductions in percent required to fit the experimental spectra in Supplementary Figure 38. Also shown are the yields obtained from electrophoresis of the "full" structure (dashed curves with symbols) and of all FRET-active constructs (solid curves with symbols).

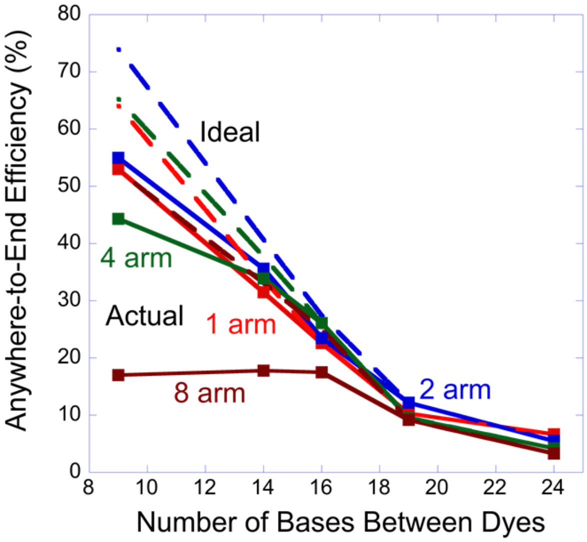

Supplementary Figure 37. Plot of the actual and ideal anywhere-to-end efficiencies (E_2) for the two-dye stars. That these curves are relatively independent of the number of arms shows that the FRET in each arm operates more or less independently.

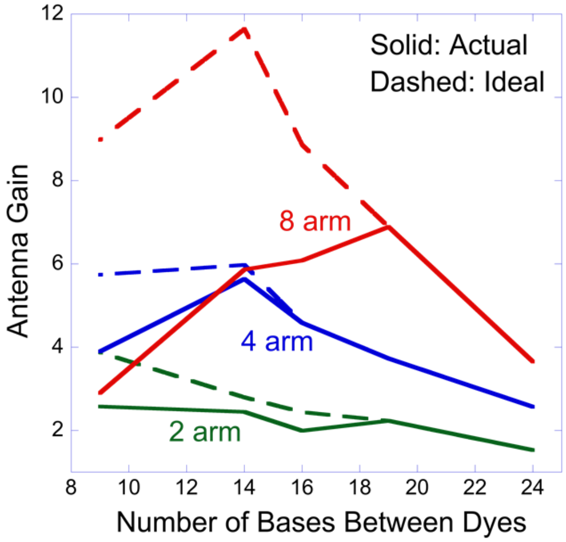

Supplementary Figure 38. **Plot of the antenna gain (AG) in the two-dye stars as a function of the distance in base pairs between the Cy3 and Cy5 attachment points.** The curves are roughly proportional to the number of arms as would be expected for independent arms, and note that AG in the 1-arm case is by definition equal to 1.

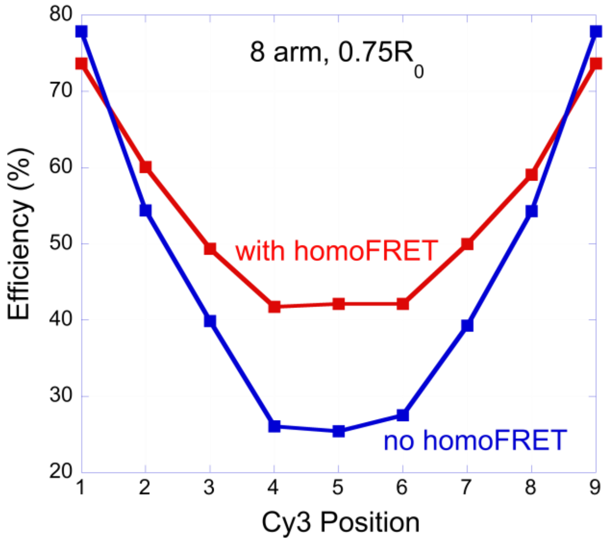

Supplementary Figure 39. Efficiency versus Cy3 position. Plot of each arm in an 8-arm two-dye star with (red curve) or without (blue curve) homoFRET processes included.

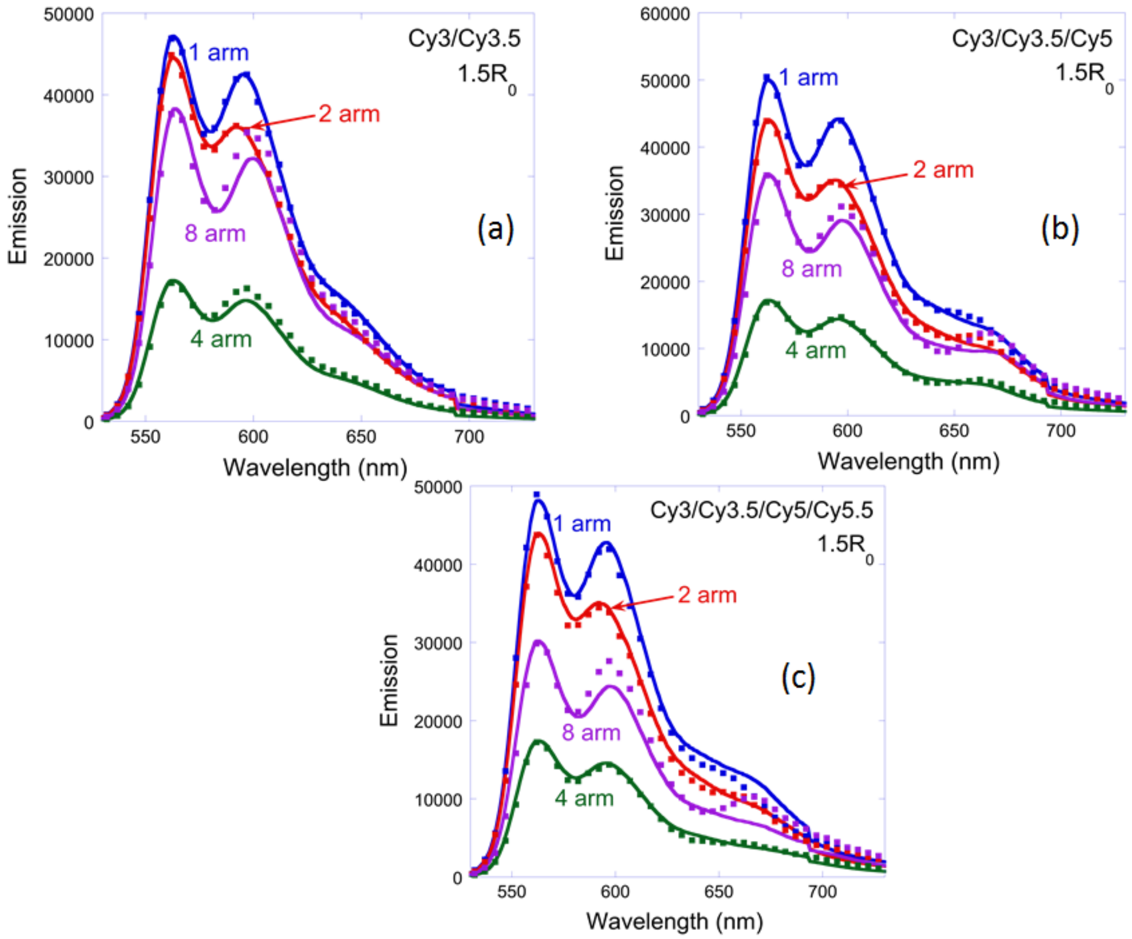

Supplementary Figure 40. Comparison of ideal simulations of the multi-dye stars at a spacing of 1.5R_0. Systems containing (**a**) two, (**b**) three, or (**c**) four dyes. The good agreement seen is to the fact that this situation is dominated by direct excitation and is relatively insensitive to all the parameters related to dye assembly and coupling.

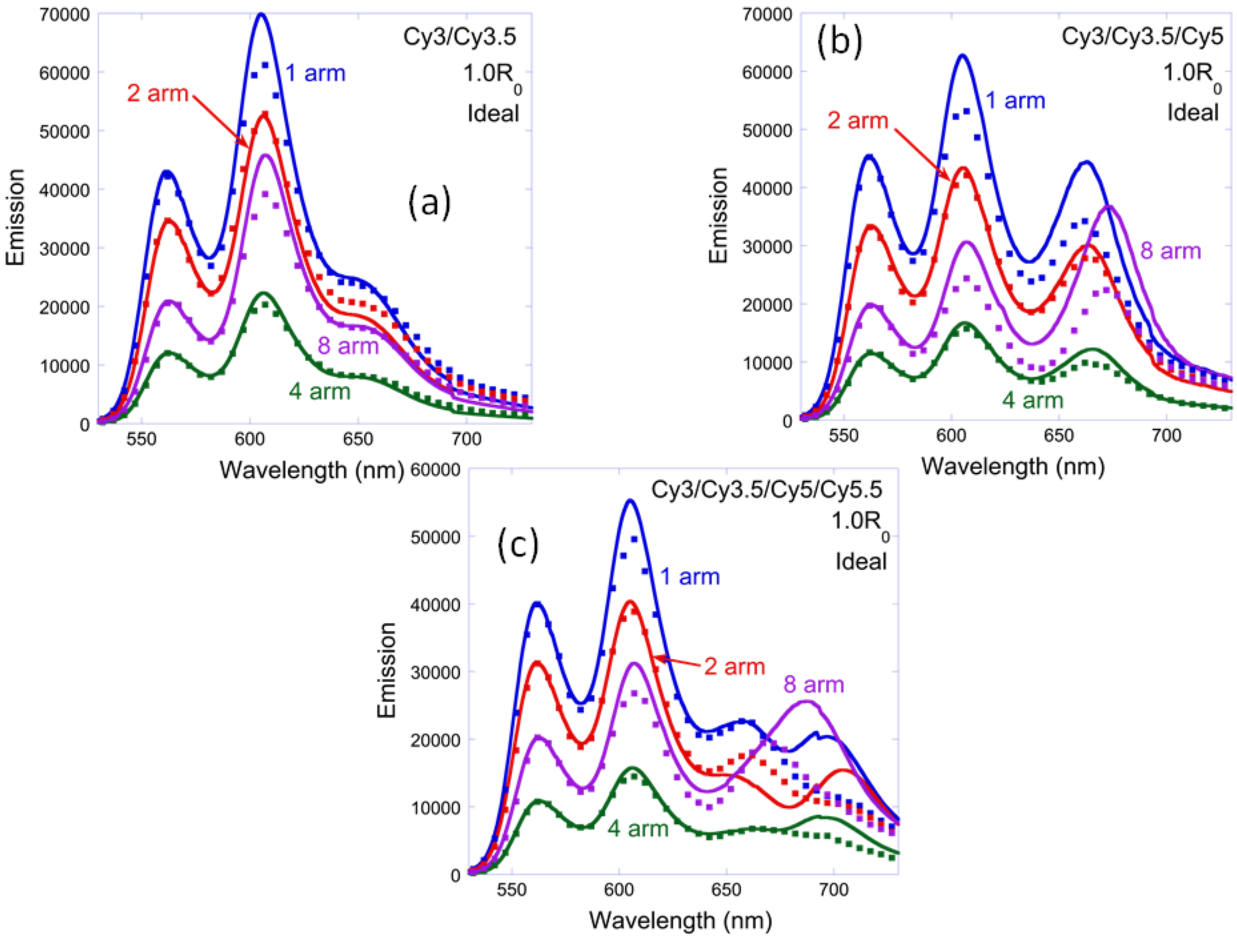

Supplementary Figure 41. Comparison of ideal simulations of the multi-dye stars at a spacing of 1.0R_0. The relatively good agreement between experiment and the ideal simulations is an indication that many aspects of the system are being properly modeled by the “ideal” treatment and especially with respect to the Cy3 and Cy3.5 dyes.

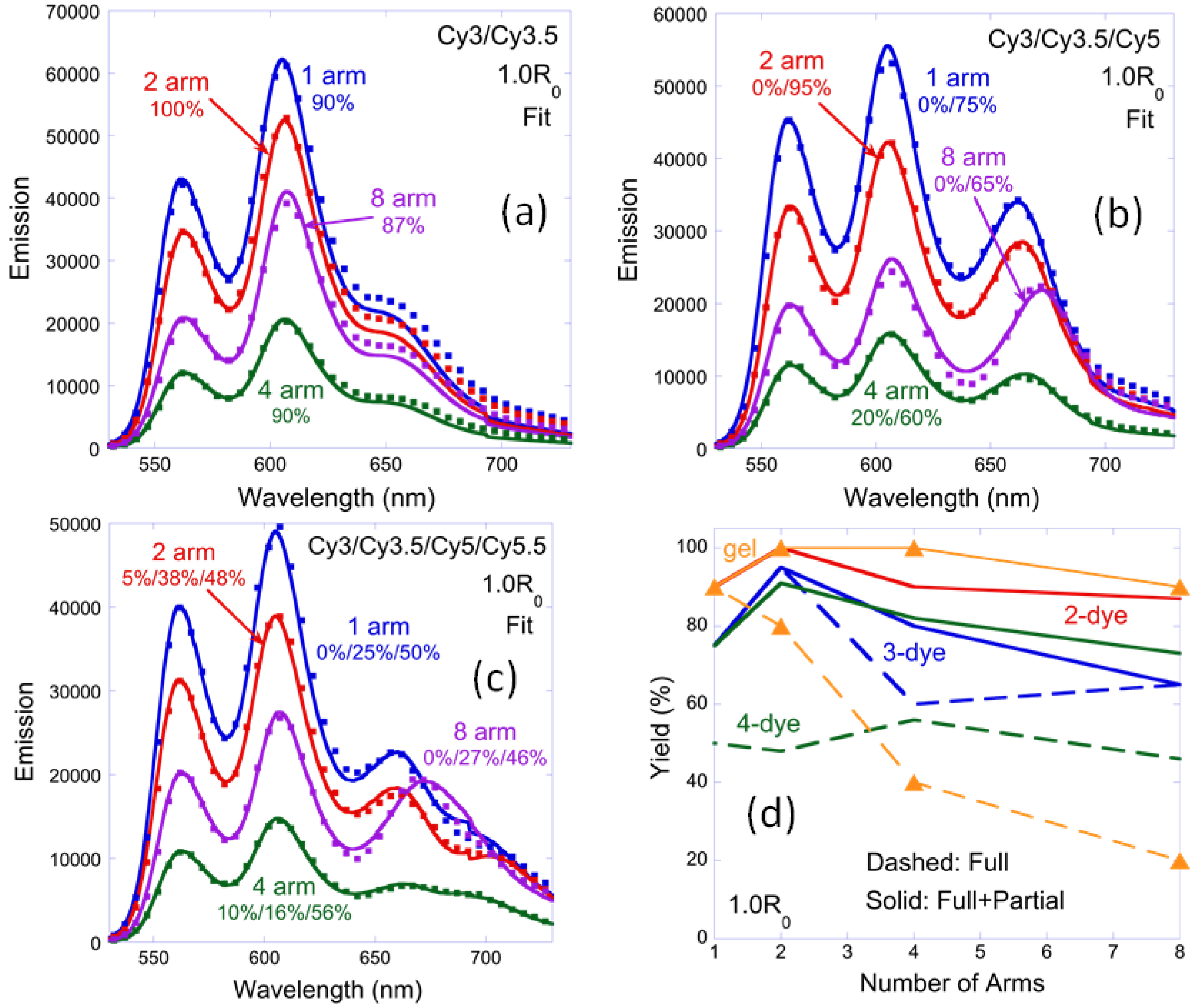

Supplementary Figure 42. (a, b, c) Fits of the model to the experimental data of Supplementary Figure 41 for the 1.0R0 multi-dye stars where yield has been used as the fitting parameter(s). The level of "yield" required for these excellent fits are indicated by the percentages accompanying each line, with the rightmost percentage being that of the full structure and the others referring to partial structures that carry one less dye type and that are meant to capture the aggregate effect of all FRET-participating partial structures in the ensemble. The dyes not included in these specific assemblies are presumed to act as "free" dyes with no FRET contribution. (**d**) Summary all of these yields with the dash lines (no symbols) referring to the simulated yield of the target structure and the solid line (no symbols) to the simulated yield of all FRET-active structures. The lines with symbols are instead from the electrophoresis experiments and correspond to the yields of full (dashed) and full+partial (solid) structures.

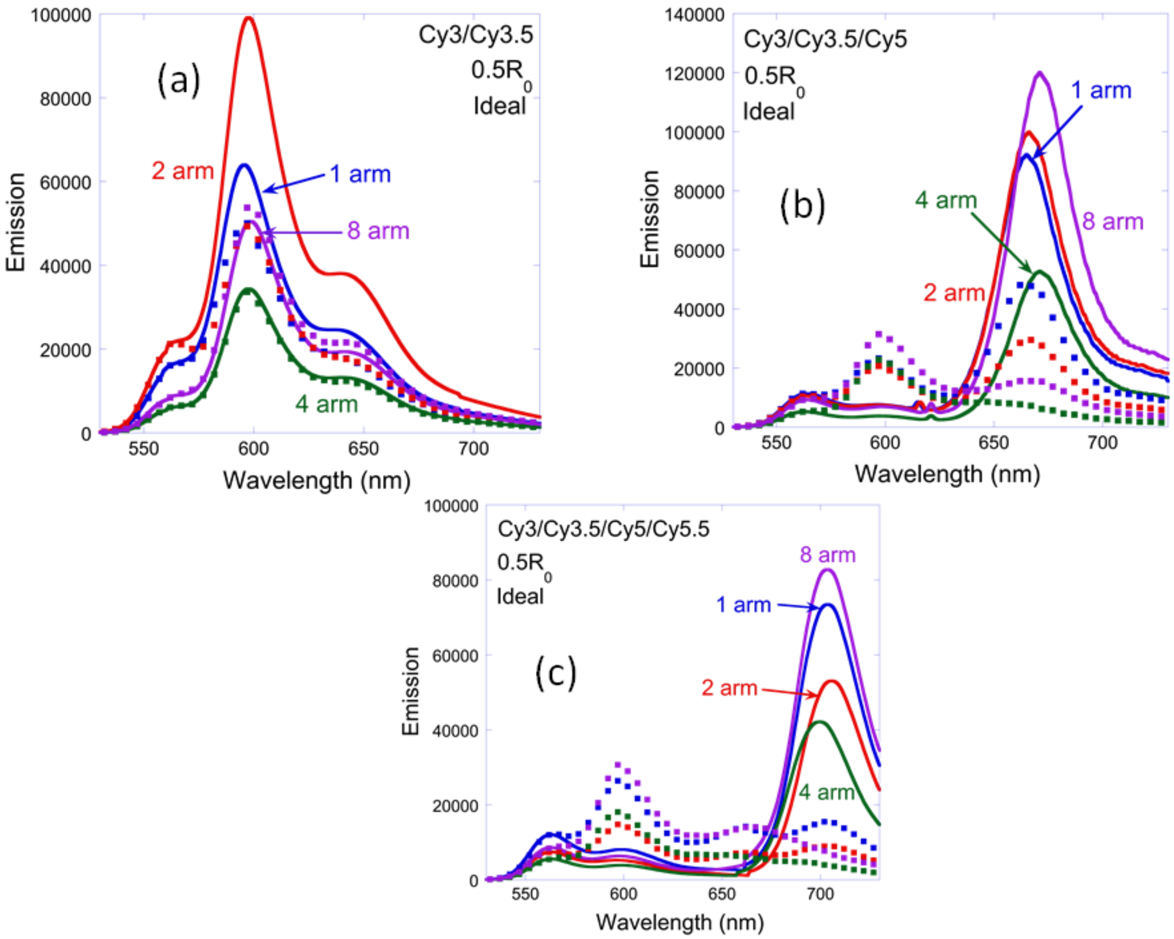

Supplementary Figure 43. Comparison of ideal simulations of the multi-dye stars at a spacing of 0.5R_0. Except when just two dyes are present (**a**), there is a complete lack of agreement between the "ideal" simulations and experiment (**b**, **c**).

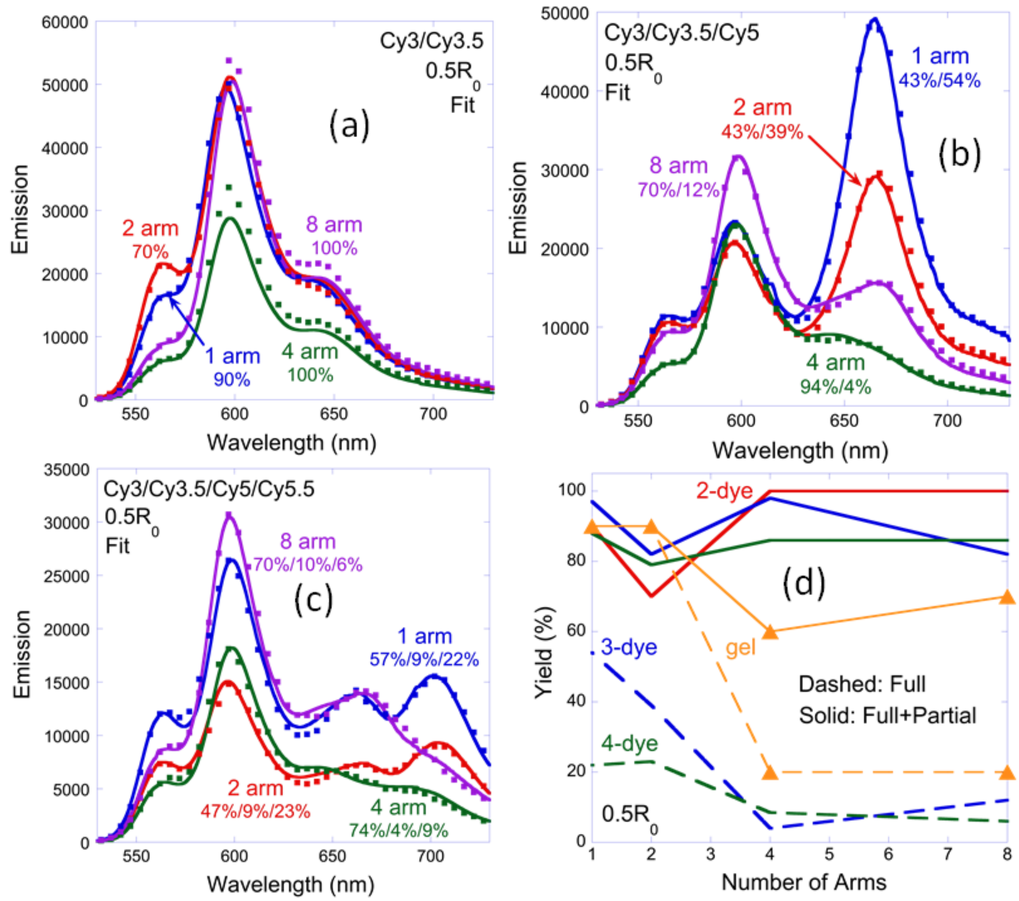

Supplementary Figure 44. The analogous plots to those of Supplementary Figure 42 for the multi-dye stars with dye spacing's of 0.5R_0.

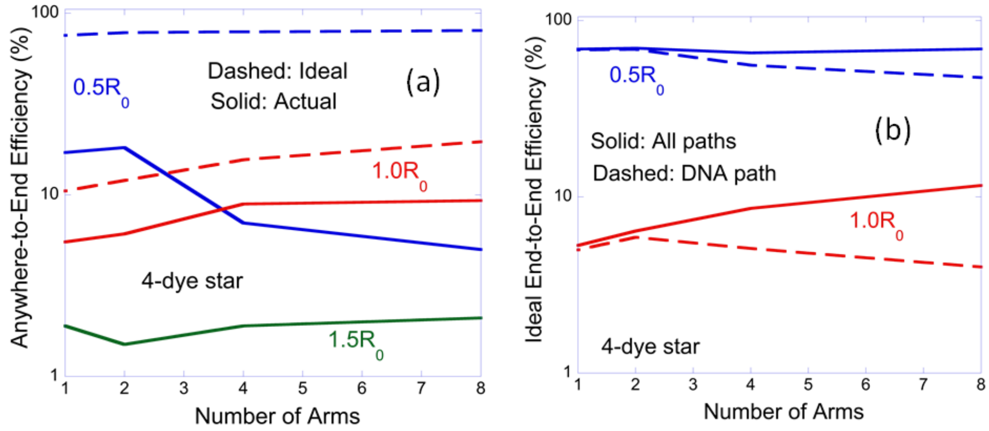

Supplementary Figure 45. (**a**) Ideal and actual anywhere-to-end efficiencies (E_1) for the multi-dye stars with spacing's of 0.5 R_0, 1.0 R_0 and 1.5 R_0. (**b**) The ideal end-to-end efficiencies (E_2) for the multi-stars with and without parallel paths included and showing that these paths do have a growing influence on the efficiency as the number of arms increases.

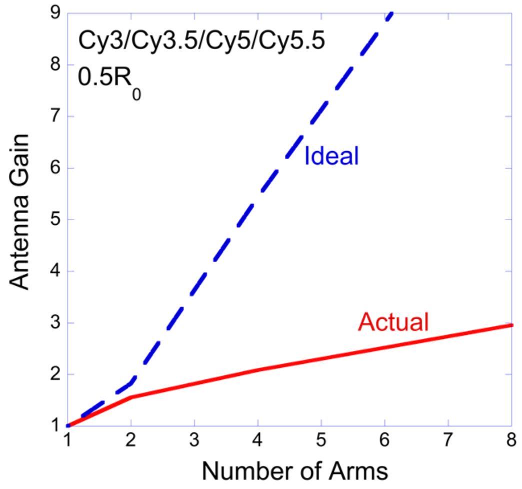

Supplementary Figure 46. Plot of the actual and ideal antenna gains (AG). Data are for the multi-dyes stars with the full complement of four dyes as a function of the number of arms.

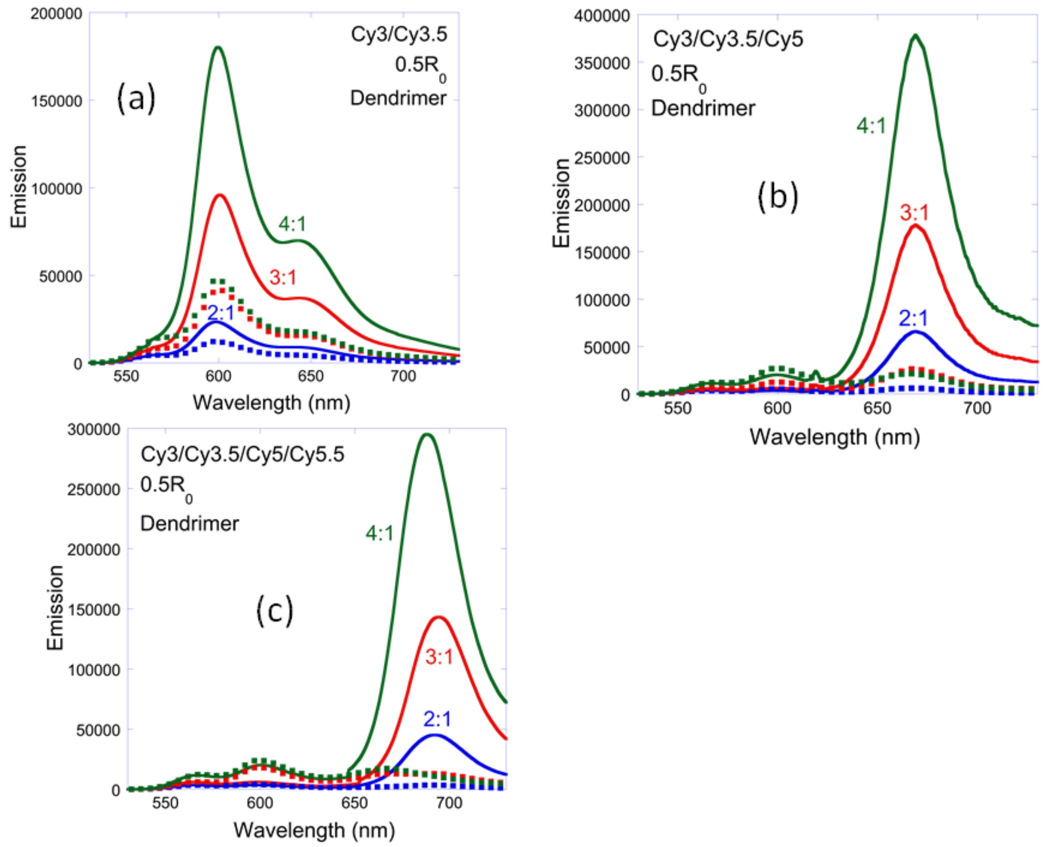

Supplementary Figure 47. Plots analogous to Supplementary Figures 40, 41 and 43. Comparison of ideal simulations with experimental spectra but here for the four-dye dendrimer designs.

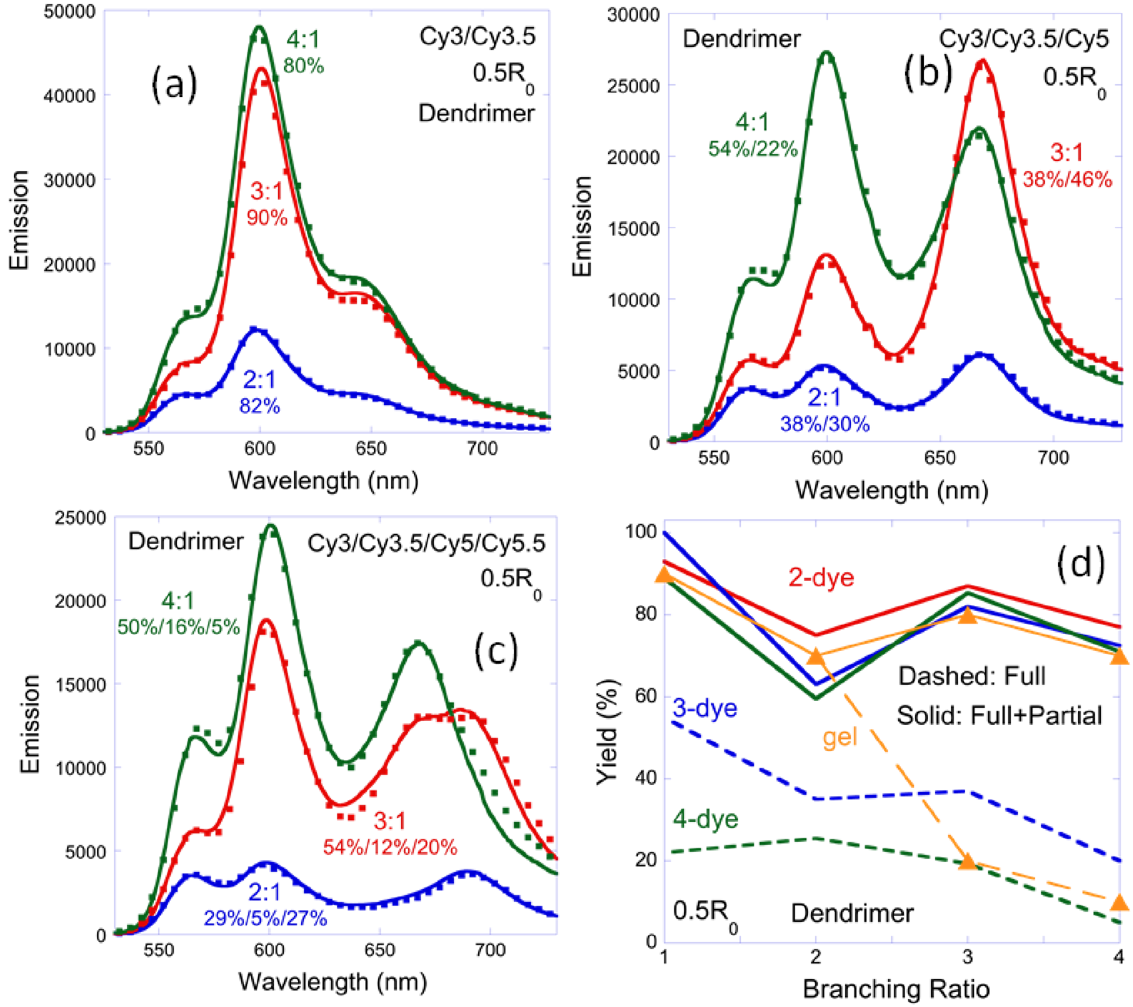

Supplementary Figure 48. Analogous plots to those of Supplementary Figures 42 and 44. Data are for the four-dye dendrimers.

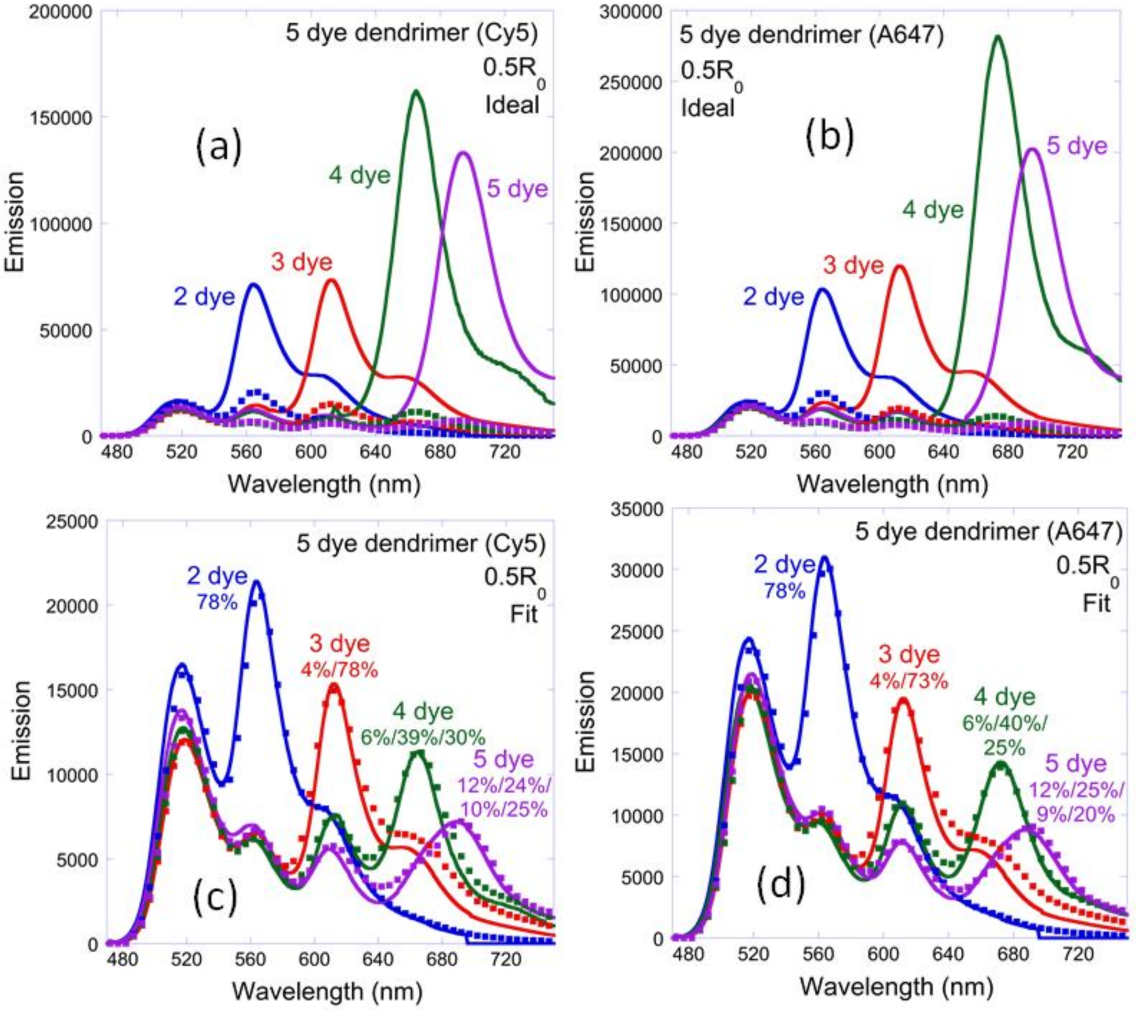

Supplementary Figure 49. Comparison of ideal simulations and experimental spectra for 5-dye dendrimers. (**a**, **b**) Plots analogous to Supplementary Figures 40, 41, 43 and 47. (**c**, **d**) Analogous plots to those of Supplementary Figures 42, 44, and 48.

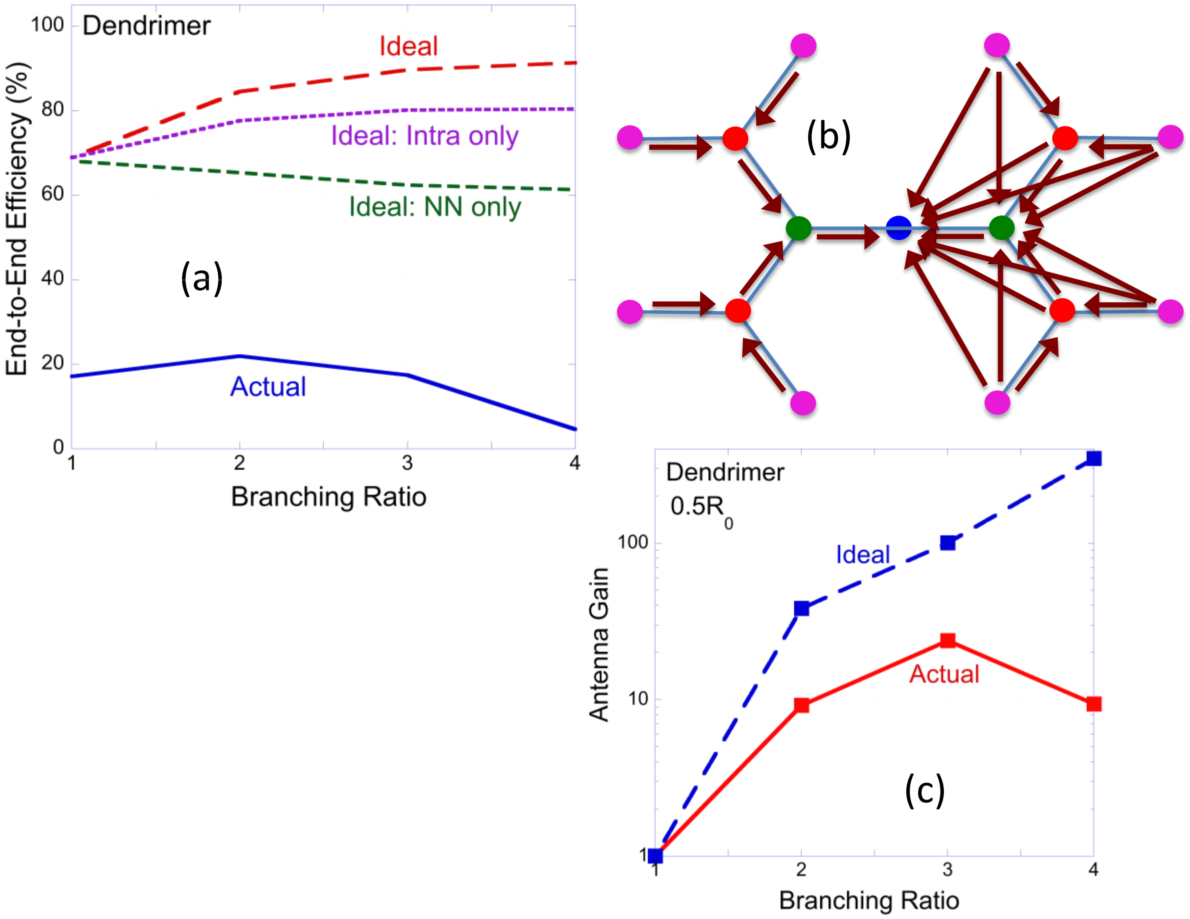

Supplementary Figure 50. Energy throughput in dendrimers. (**a**) Ideal and actual end-to-end efficiencies (*E*2) in the four-dye dendrimers. The "ideal" E2 is broken into a total and the amount when only nearest neighbors dye paths (green dashed) and when all intra-arm paths (purple small-dashed) are included as depicted in the left and right sides of (**b**), respectively. (**c**) The "ideal" and "actual" antenna gains (AG) of the four-dye dendrimers versus branching ratio.

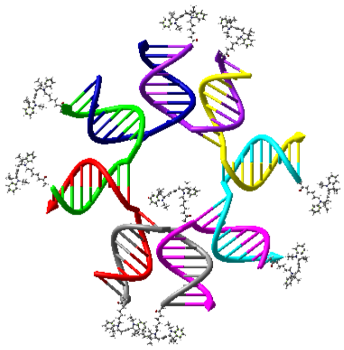

Supplementary Figure 51. Model of the 8-arm star. This highlights the asymmetric placement of the internal Cy5 dye relative to the surrounding Cy3 dyes.

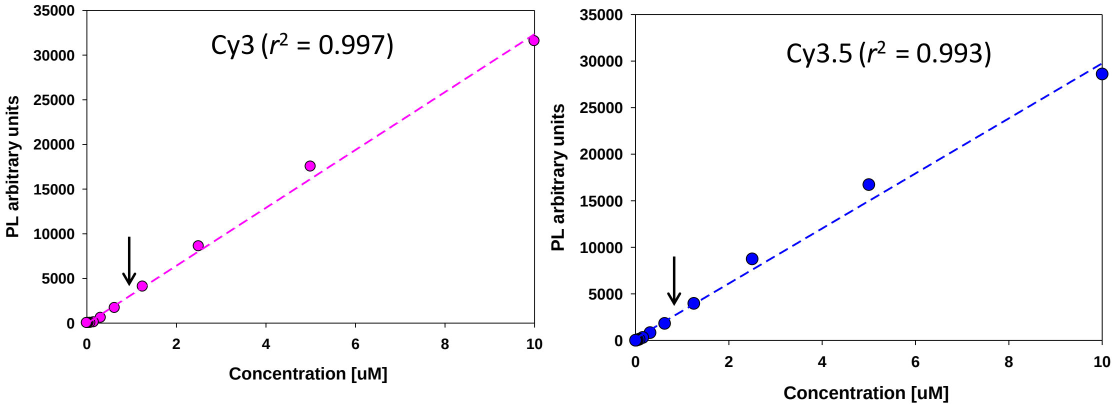

Supplementary Figure 52. Representative lots of Cy3 and Cy3.5 fluorescence range. Data are from control structures collected on the Tecan Fluorometer at typical instrumental settings and sample volumes. Note the linear response of fluorescence versus concentration. Typical sample concentrations were ~ 1 μM as indicated by the arrows in each plot.

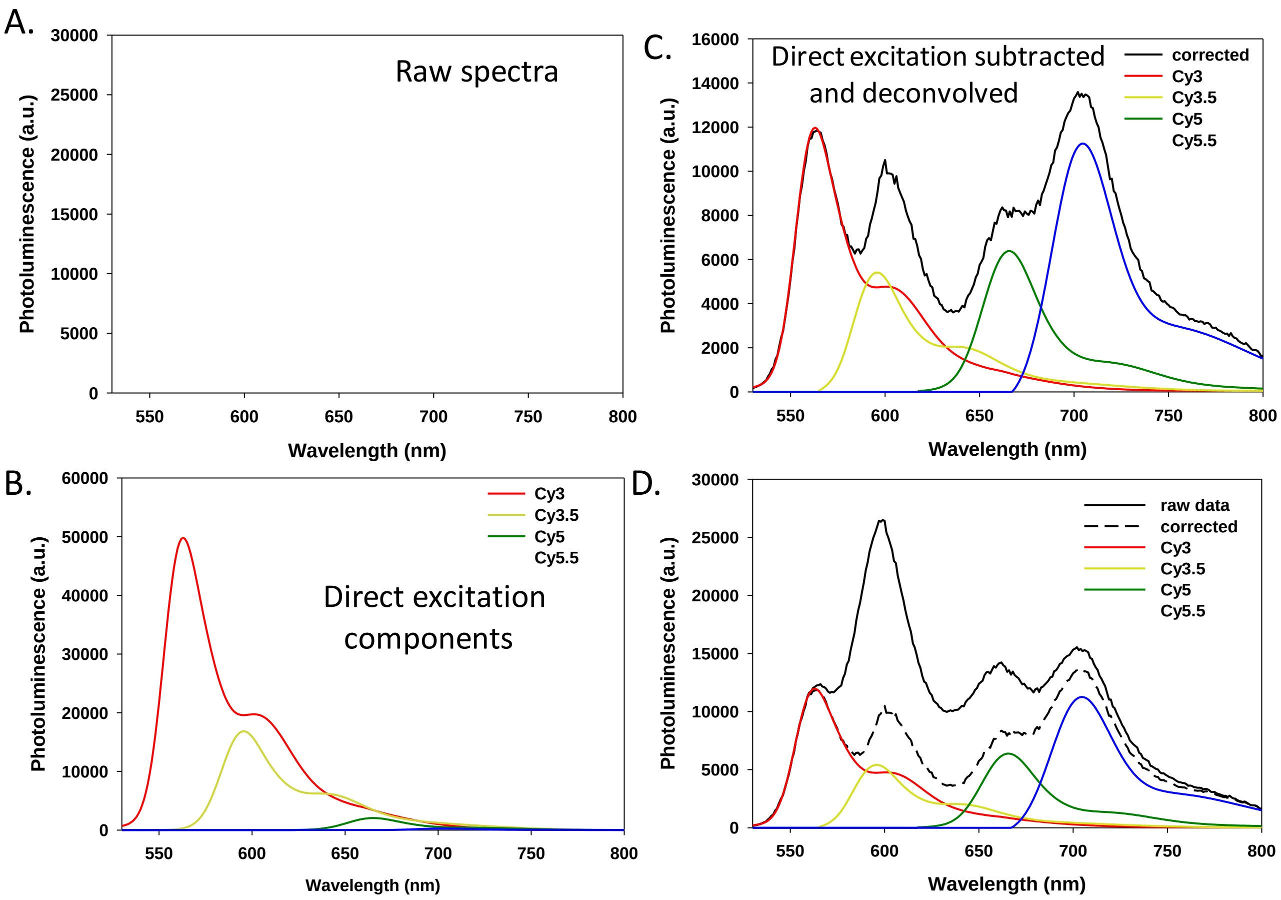

Supplementary Figure 53. Representative example of how data was decomposed and processed. (**A**) Raw spectra collected from the 0.5×R_0 [Cy3→Cy3.5→Cy5]$_1$→Cy5.5 linear construct excited at 515 nm with spectral data collected from 530 nm to 850 nm as described. (**B**) Direct excitation components for each of the individual dyes collected from fully assembled control structures containing only that dye label. (**C**) Direct excitation subtracted spectrum along with the decomposed spectra corresponding to the quenched initial Cy3 donor and the sensitized component for each of the remaining dyes. (**D**) Superimposition of (**A**) and (**C**).

Supplementary Table 1. DNA Sequences for 2-dye, 8-way, 1.5×R_0.

Name	Sequence	Modification	Tm (1X/ 2.5X PBS)	Source
**T1**	*GGAGAGATGGTTCAGCCGCAATCCTCGCC TGCACTCTACCTGACTTCC*	5' Cy3, 3' Cy3	78.8/ 83.8	IDT
**T2**	GGAAGTCAGGTAGAGTGCAGGCGA*GAGC ACGAGTCTTGCTGCTTAGC	Internal Cy5	78.2/ 83.2	IDT
**T3**	*GCTAAGCAGCAAGACTCGTGCTCACCGAA TGCCACCACGCTCCGTCGC*	5' Cy3, 3' Cy3	80.9/ 85.7	IDT
**T4**	GCGACGGAGCGTGGTGGCATTCGGCGTCCA GCTCTGATCCAATACTCC		80.6/ 85.4	IDT
**T5**	*GGAGTATTGGATCAGAGCTGGACGACAATG ACGTAGGTCCTAACCTCC*	5' Cy3, 3' Cy3	75.8/ 81.0	IDT
**T6**	GGAGGTTAGGACCTACGTCATTGTACTATG GCACACATCCCTAGTTCC		75.1/ 80.4	IDT
**T7**	*GGAACTAGGGATGTGTGCCATAGTGGTCA ACGCATACACCTTCTATCC*	5' Cy3, 3' Cy3	75.2/ 80.9	IDT
**T8**	GGATAGAAGGTGTATGCGTTGACCGGATTG CGGCTGAACCATCTCTCC		77.4/ 82.6	IDT

Notes: Tm's are predicted values. All Cy3 and Cy5 dyes used where phosphoramidites. Unless otherwise indicated (ex. ester = es), the dyes are inserted as phosphoramidites.*in sequence indicates modifier placement.

Supplementary Table 2. DNA Sequences - 2-dye, 4-way, 1.5×R_0.

Name	Sequence	Modification	Tm (1X/ 2.5X PBS)	Source
**T1**	*GGAGAGATGGTTCAGCCGCAATCCTCGCCT GCACTCTACCTGACTTCC*	5' Cy3, 3' Cy3	78.8/ 83.8	IDT
**T2**	GGAAGTCAGGTAGAGTGCAGGCGA*GAGC ACGAGTCTTGCTGCTTAGC	Internal Cy5	78.2/ 83.2	IDT
**T3**	*GCTAAGCAGCAAGACTCGTGCTCACCGAAT GCCACCACGCTCCGTCGC*	5' Cy3, 3' Cy3	80.9/ 85.7	IDT
**T9**	GCGACGGAGCGTGGTGGCATTCGGGGATTG CGGCTGAACCATCTCTCC		81.8/ 86.5	IDT

Notes: Tm's are predicted values. All Cy3 and Cy5 dyes used where phosphoramidites. Unless otherwise indicated (ex. ester = es), the dyes are inserted as phosphoramidites.*in sequence indicates modifier placement.

Supplementary Table 3. DNA Sequences - 2-dye, 2-way, 1.5×R_0.

Name	Sequence	Modification	Tm (1X/ 2.5X PBS)	Source
**T5**	*GGAGTATTGGATCAGAGCTGGACGACAATG ACGTAGGTCCTAACCTCC*	5' Cy3, 3' Cy3	75.8/ 81.0	IDT
**T10**	GGAGGTTAGGACCTACGTCATTG*CGTCCA GCTCTGATCCAATACTCC	Internal Cy5	76.2/ 81.4	IDT

Notes: Tm's are predicted values. All Cy3 and Cy5 dyes used where phosphoramidites. Unless otherwise indicated (ex. ester = es), the dyes are inserted as phosphoramidites.*in sequence indicates modifier placement.

Supplementary Table 4. DNA Sequences - 2-dye, 1-way, 1.5×R_0.

Name	Sequence	Modification	Tm (1X/ 2.5X PBS)	Source
**T5**	*GGAGTATTGGATCAGAGCTGGACGACAATG ACGTAGGTCCTAACCTCC	5’ Cy3	75.8/ 81.0	IDT
**T10**	GGAGGTTAGGACCTACGTCATTG*CGTCCA GCTCTGATCCAATACTCC	Internal Cy5	76.2/ 81.4	IDT

Notes: Tm’s are predicted values. All Cy3 and Cy5 dyes used where phosphoramidites. Unless otherwise indicated (ex. ester = es), the dyes are inserted as phosphoramidites.*in sequence indicates modifier placement.

Supplementary Table 5. DNA Sequences - 2-dye, 8-way, 1.25×R_0.

Name	Sequence	Modification	Tm (1X/ 2.5X PBS)	Source
**T1**	*GATGGTTCAGCCGCAATCCTCGCCTGCAC TCTACCTGA*	5’ Cy3, 3’ Cy3	77.4/ 82.3	IDT
**T2**	TCAGGTAGAGTGCAGGCGA*GAGCACGAG TCTTGCTGC	Internal Cy5	77.4/ 82.2	IDT
**T3**	*GCAGCAAGACTCGTGCTCACCGAATGCCA CCACGCTCC*	5’ Cy3, 3’ Cy3	79.1/ 83.9	IDT
**T4**	GGAGCGTGGTGGCATTCGGCGTCCAGCTCT GATCCAAT		78.9/ 83.8	IDT
**T5**	*ATTGGATCAGAGCTGGACGACAATGACGT AGGTCCTAA*	5’ Cy3, 3’ Cy3	73.4/ 78.7	IDT
**T6**	TTAGGACCTACGTCATTGTACTATGGCACA CATCCCTA		71.9/ 77.3	IDT
**T7**	*TAGGGATGTGTGCCATAGTGGTCAACGCA TACACCTTC*	5’ Cy3, 3’ Cy3	74.1/ 79.3	IDT
**T8**	GAAGGTGTATGCGTTGACCGGATTGCGGCT GAACCATC		76.0/ 81.1	IDT

Notes: Tm’s are predicted values. All Cy3 and Cy5 dyes used where phosphoramidites. Unless otherwise indicated (ex. ester = es), the dyes are inserted as phosphoramidites.*in sequence indicates modifier placement.

Supplementary Table 6. DNA Sequences - 2-dye, 4-way, 1.25×R_0.

Name	Sequence	Modification	Tm (1X/ 2.5X PBS)	Source
**T1**	*GATGGTTCAGCCGCAATCCTCGCCTGCAC TCTACCTGA*	5’ Cy3, 3’ Cy3	77.4/ 82.3	IDT
**T2**	TCAGGTAGAGTGCAGGCGA*GAGCACGAG TCTTGCTGC	Internal Cy5	77.4/ 82.2	IDT
**T3**	*GCAGCAAGACTCGTGCTCACCGAATGCCA CCACGCTCC*	5’ Cy3, 3’ Cy3	79.1/ 83.9	IDT
**T9**	GGAGCGTGGTGGCATTCGGGGATTGCGGCT GAACCATC		79.5/ 84.2	IDT

Notes: Tm’s are predicted values. All Cy3 and Cy5 dyes used where phosphoramidites. Unless otherwise indicated (ex. ester = es), the dyes are inserted as phosphoramidites.*in sequence indicates modifier placement.

Supplementary Table 7. DNA Sequences - 2-dye, 2-way, 1.25×R_0.

Name	Sequence	Modification	Tm (1X/ 2.5X PBS)	Source
**T5**	* ATTGGATCAGAGCTGGACGACAATGACGT AGGTCCTAA*	5' Cy3, 3' Cy3	73.4/ 78.7	IDT
**T10**	TTAGGACCTACGTCATTG*CGTCCAGCTCTG ATCCAAT	Internal Cy5	73.8/ 79.1	IDT

Notes: Tm's are predicted values. All Cy3 and Cy5 dyes used where phosphoramidites. Unless otherwise indicated (ex. ester = es), the dyes are inserted as phosphoramidites.*in sequence indicates modifier placement.

Supplementary Table 8. DNA Sequences - 2-dye, 1-way, 1.25×R_0.

Name	Sequence	Modification	Tm (1X/ 2.5X PBS)	Source
**T5**	* ATTGGATCAGAGCTGGACGACAATGACGT AGGTCCTAA	5' Cy3	73.4/ 78.7	IDT
**T10**	TTAGGACCTACGTCATTG*CGTCCAGCTCTG ATCCAAT	Internal Cy5	73.8/ 79.1	IDT

Notes: Tm's are predicted values. All Cy3 and Cy5 dyes used where phosphoramidites. Unless otherwise indicated (ex. ester = es), the dyes are inserted as phosphoramidites.*in sequence indicates modifier placement.

Supplementary Table 9. DNA Sequences - 2-dye, 8-way, 1.0×R_0.

Name	Sequence	Modification	Tm (1X/ 2.5X PBS)	Source
**T1**	*GGTTCAGCCGCAATCCTCGCCTGCACTCT ACC*	5' Cy3, 3' Cy3	76.2/ 80.9	IDT
**T2**	GGTAGAGTGCAGGCGA*GAGCACGAGTCT TGC	Internal Cy5	74.6/ 79.3	IDT
**T3**	*GCAAGACTCGTGCTCACCGAATGCCACCA CGC*	5' Cy3, 3' Cy3	76.8/ 81.5	IDT
**T4**	GCGTGGTGGCATTCGGCGTCCAGCTCTGAT CC		77.8/ 82.4	IDT
**T5**	*GGATCAGAGCTGGACGACAATGACGTAGG TCC*	5' Cy3, 3' Cy3	72.5/ 77.4	IDT
**T6**	GGACCTACGTCATTGTACTATGGCACACAT CC		70.2/ 75.3	IDT
**T7**	*GGATGTGTGCCATAGTGGTCAACGCATAC ACC*	5' Cy3, 3' Cy3	72.4/ 77.4	IDT
**T8**	GGTGTATGCGTTGACCGGATTGCGGCTGAA CC		75.5/ 80.3	IDT

Notes: Tm's are predicted values. All Cy3 and Cy5 dyes used where phosphoramidites. Unless otherwise indicated (ex. ester = es), the dyes are inserted as phosphoramidites.*in sequence indicates modifier placement.

Supplementary Table 10. DNA Sequences - 2-dye, 4-way, 1.0×R_0.

Name	Sequence	Modification	Tm (1X/ 2.5X PBS)	Source
**T1**	*GGTTCAGCCGCAATCCTCGCCTGCACTCT ACC*	5' Cy3, 3' Cy3	76.2/ 80.9	IDT
**T2**	GGTAGAGTGCAGGCGA*GAGCACGAGTCT TGC	Internal Cy5	74.6/ 79.3	IDT
**T3**	*GCAAGACTCGTGCTCACCGAATGCCACCA CGC*	5' Cy3, 3' Cy3	76.8/ 81.5	IDT
**T9**	GCGTGGTGGCATTCGGGGATTGCGGCTGAA CC		78.6/ 83.2	IDT

Notes: Tm's are predicted values. All Cy3 and Cy5 dyes used where phosphoramidites. Unless otherwise indicated (ex. ester = es), the dyes are inserted as phosphoramidites.*in sequence indicates modifier placement.

Supplementary Table 11. DNA Sequences - 2-dye, 2-way, 1.0×R_0.

Name	Sequence	Modification	Tm (1X/ 2.5X PBS)	Source
**T5**	*GGATCAGAGCTGGACGACAATGACGTAGG TCC*	5' Cy3, 3' Cy3	72.5/ 77.4	IDT
**T10**	GGACCTACGTCATTG*CGTCCAGCTCTGA TCC	Internal Cy5	73.1/77.9	IDT

Notes: Tm's are predicted values. All Cy3 and Cy5 dyes used where phosphoramidites. Unless otherwise indicated (ex. ester = es), the dyes are inserted as phosphoramidites.*in sequence indicates modifier placement.

Supplementary Table 12. DNA Sequences - 2-dye, 1-way, 1.0×R_0.

Name	Sequence	Modification	Tm (1X/ 2.5X PBS)	Source
**T5**	*GGATCAGAGCTGGACGACAATGACGTAGG TCC	5' Cy3	72.5/ 77.4	IDT
**T10**	GGACCTACGTCATTG*CGTCCAGCTCTGA TCC	Internal Cy5	73.1/77.9	IDT

Notes: Tm's are predicted values. All Cy3 and Cy5 dyes used where phosphoramidites. Unless otherwise indicated (ex. ester = es), the dyes are inserted as phosphoramidites.*in sequence indicates modifier placement.

Supplementary Table 13. DNA Sequences - 2-dye, 8-way, 0.85×R_0.

Name	Sequence	Modification	Tm (1X/ 2.5X PBS)	Source
**T1**	*TTCAGCCGCAATCCTCGCCTGCACTCTA*	5’ Cy3, 3’ Cy3	74.0/ 78.9	IDT
**T2**	TAGAGTGCAGGCGA*****GAGCACGAGTCTT	Internal Cy5	71.8/76.7	IDT
**T3**	*AAGACTCGTGCTCACCGAATGCCACCAC*	5’ Cy3, 3’ Cy3	73.0/ 77.9	IDT
**T4**	GTGGTGGCATTCGGCGTCCAGCTCTGAT		74.7/ 79.4	IDT
**T5**	*ATCAGAGCTGGACGACAATGACGTAGGT*	5’ Cy3, 3’ Cy3	70.0/ 75.2	IDT
**T6**	ACCTACGTCATTGTACTATGGCACACAT		67.2/ 72.6	IDT
**T7**	*ATGTGTGCCATAGTGGTCAACGCATACA*	5’ Cy3, 3’ Cy3	69.6/ 74.9	IDT
**T8**	TGTATGCGTTGACCGGATTGCGGCTGAA		73.1/ 78.2	IDT

Notes: Tm’s are predicted values. All Cy3 and Cy5 dyes used where phosphoramidites. Unless otherwise indicated (ex. ester = es), the dyes are inserted as phosphoramidites.*in sequence indicates modifier placement.

Supplementary Table 14. DNA Sequences - 2-dye, 4-way, 0.85×R_0.

Name	Sequence	Modification	Tm (1X/ 2.5X PBS)	Source
**T1**	*TTCAGCCGCAATCCTCGCCTGCACTCTA*	5’ Cy3, 3’ Cy3	74.0/ 78.9	IDT
**T2**	TAGAGTGCAGGCGA*****GAGCACGAGTCTT	Internal Cy5	71.8/76.7	IDT
**T3**	*AAGACTCGTGCTCACCGAATGCCACCAC*	5’ Cy3, 3’ Cy3	73.0/ 77.9	IDT
**T9**	GTGGTGGCATTCGGGGATTGCGGCTGAA		75.4/ 80.1	IDT

Notes: Tm’s are predicted values. All Cy3 and Cy5 dyes used where phosphoramidites. Unless otherwise indicated (ex. ester = es), the dyes are inserted as phosphoramidites.*in sequence indicates modifier placement.

Supplementary Table 15. DNA Sequences - 2-dye, 2-way, 0.85×R_0.

Name	Sequence	Modification	Tm (1X/ 2.5X PBS)	Source
**T5**	*ATCAGAGCTGGACGACAATGACGTAGGT*	5’ Cy3, 3’ Cy3	70.0/ 75.2	IDT
**T10**	ACCTACGTCATTG*CGTCCAGCTCTGAT	Internal Cy5	70.6/ 75.7	IDT

Notes: Tm’s are predicted values. All Cy3 and Cy5 dyes used where phosphoramidites. Unless otherwise indicated (ex. ester = es), the dyes are inserted as phosphoramidites.*in sequence indicates modifier placement.

Supplementary Table 16. DNA Sequences - 2-dye, 1-way, 0.85×R_0.

Name	Sequence	Modification	Tm (1X/ 2.5X PBS)	Source
**T5**	*ATCAGAGCTGGACGACAATGACGTAGGT	5’ Cy3	70.0/ 75.2	IDT
**T10**	ACCTACGTCATTG*CGTCCAGCTCTGAT	Internal Cy5	70.6/ 75.7	IDT

Notes: Tm’s are predicted values. All Cy3 and Cy5 dyes used where phosphoramidites. Unless otherwise indicated (ex. ester = es), the dyes are inserted as phosphoramidites.*in sequence indicates modifier placement.

Supplementary Table 17. DNA Sequences - 2-dye, 8-way, 0.75×R_0.

Name	Sequence	Modification	Tm (1X/ 2.5X PBS)	Source
**T1**	*CCGCAATCCTCGCCTGCA*	5' Cy3, 3' Cy3	68.0/ 72.2	IDT
**T2**	TGCAGGCGA*GAGCACGA	Internal Cy5	66.6/ 70.9	IDT
**T3**	*TCGTGCTCACCGAATGCC*	5' Cy3, 3' Cy3	65.1/ 69.5	IDT
**T4**	GGCATTCGGCGTCCAGCT		68.0/ 72.2	IDT
**T5**	*AGCTGGACGACAATGACG*	5' Cy3, 3' Cy3	62.0/ 66.6	IDT
**T6**	CGTCATTGTACTATGGCA		56.4/ 61.3	IDT
**T7**	*TGCCATAGTGGTCAACGC*	5' Cy3, 3' Cy3	62.4/ 67.1	IDT
**T8**	GCGTTGACCGGATTGCGG		66.6/ 70.8	IDT

Notes: Tm's are predicted values. All Cy3 and Cy5 dyes used where phosphoramidites. Unless otherwise indicated (ex. ester = es), the dyes are inserted as phosphoramidites.*in sequence indicates modifier placement.

Supplementary Table 18. DNA Sequences - 2-dye, 4-way, 0.75×R_0.

Name	Sequence	Modification	Tm (1X/ 2.5X PBS)	Source
**T1**	*CCGCAATCCTCGCCTGCA*	5' Cy3, 3' Cy3	68.0/ 72.2	IDT
**T2**	TGCAGGCGA*GAGCACGA	Internal Cy5	66.6/ 70.9	IDT
**T3**	*TCGTGCTCACCGAATGCC*	5' Cy3, 3' Cy3	65.1/ 69.5	IDT
**T9**	GGCATTCGGGGATTGCGG		66.4/ 70.6	IDT

Notes: Tm's are predicted values. All Cy3 and Cy5 dyes used where phosphoramidites. Unless otherwise indicated (ex. ester = es), the dyes are inserted as phosphoramidites.*in sequence indicates modifier placement.

Supplementary Table 19. DNA Sequences - 2-dye, 2-way, 0.75×R_0.

Name	Sequence	Modification	Tm (1X/ 2.5X PBS)	Source
**T5**	*AGCTGGACGACAATGACG*	5' Cy3, 3' Cy3	62.0/ 66.6	IDT
**T10**	CGTCATTG*CGTCCAGCT	Internal Cy5	62.5/ 67.0	IDT

Notes: Tm's are predicted values. All Cy3 and Cy5 dyes used where phosphoramidites. Unless otherwise indicated (ex. ester = es), the dyes are inserted as phosphoramidites.*in sequence indicates modifier placement.

Supplementary Table 20. DNA Sequences - 2-dye, 1-way, 0.75×R_0.

Name	Sequence	Modification	Tm (1X/ 2.5X PBS)	Source
**T5**	*AGCTGGACGACAATGACG	5' Cy3	62.0/ 66.6	IDT
**T10**	CGTCATTG*CGTCCAGCT	Internal Cy5	62.5/ 67.0	IDT

Notes: Tm's are predicted values. All Cy3 and Cy5 dyes used where phosphoramidites. Unless otherwise indicated (ex. ester = es), the dyes are inserted as phosphoramidites.*in sequence indicates modifier placement.

Supplementary Table 21. DNA Sequences - 4-dye, 8-way, 1.5×R_0.

Name	Sequence	Modification	Tm (1X/ 2.5X PBS)	Source
**A**	*AAAAGACTATTAATCG	5' Cy3	43.5/ 48.8	IDT
**B**	ATAATT*AAAATTAGAT<br>+Cy3.5es	Internal Am-Uni	35.5/ 41.7	Operon
**C**	ATAAGATAATAGATCGAATA*	3' Cy5	47.4/ 53.1	IDT
**T1**	GAGATGGTTCAGCCGCAATCCT*CGCCTGC<br>ACTCTACCTGACTTATTCGATCTATTATCTTA<br>TATCTAATTTTAATTATCGATTAATAGTCTTTT	Internal Am-Uni<br>+Cy5.5es	76.7/ 82.7	Operon
**T2**	AGTCAGGTAGAGTGCAGGCGATGAGCACG<br>AGTCTTGCTGCTTTATTCGATCTATTATCTTA<br>TATCTAATTTTAATTATCGATTAATAGTCTTTT		76.7/ 83.0	IDT
**T3**	AAGCAGCAAGACTCGTGCTCACCGAATGCC<br>ACCACGCTCCGTTATTCGATCTATTATCTTA<br>TATCTAATTTTAATTATCGATTAATAGTCTTTT		77.6/ 83.7	IDT
**T4**	ACGGAGCGTGGTGGCATTCGGCGTCCAGC<br>TCTGATCCAATACTATTCGATCTATTATCTTA<br>TATCTAATTTTAATTATCGATTAATAGTCTTTT		77.3/ 83.4	IDT
**T5**	GTATTGGATCAGAGCTGGACGACAATGACG<br>TAGGTCCTAACCTATTCGATCTATTATCTTATA<br>TCTAATTTTAATTATCGATTAATAGTCTTTT		75.7/ 81.8	IDT
**T6**	GGTTAGGACCTACGTCATTGTACTATGGCA<br>CACATCCCTAGTTATTCGATCTATTATCTTA<br>TATCTAATTTTAATTATCGATTAATAGTCTTTT		75.5/ 81.7	IDT
**T7**	ACTAGGGATGTGTGCCATAGTGGTCAACGC<br>ATACACCTTCTATATTCGATCTATTATCTTATA<br>TCTAATTTTAATTATCGATTAATAGTCTTTT		76.0/ 82.3	IDT
**T8**	TAGAAGGTGTATGCGTTGACCGGATTGCGG<br>CTGAACCATCTCTATTCGATCTATTATCTTAT<br>ATCTAATTTTAATTATCGATTAATAGTCTTTT		76.6/ 82.8	IDT

Notes: Tm's are predicted values. All Cy3 and Cy5 dyes used where phosphoramidites. Unless otherwise indicated (ex. ester = es), the dyes are inserted as phosphoramidites.*in sequence indicates modifier placement.

Supplementary Table 22. DNA Sequences - 4-dye, 4-way, 1.5×R_0.

Name	Sequence	Modification	Tm (1X/ 2.5X PBS)	Source
**A**	*AAAAGACTATTAATCG	5' Cy3	43.5/ 48.8	IDT
**B**	ATAATT*AAAATTAGAT	Internal Am-Uni +Cy3.5es	35.5/ 41.7	Operon
**C**	ATAAGATAATAGATCGAATA*	3' Cy5	47.4/ 53.1	IDT
**T9**	TACGAGGGAATTACAGGGTGT*TGTTAGATT AAATAGTAAATGTATTCGATCTATTATCTTAT ATCTAATTTTAATTATCGATTAATAGTCTTTT	Internal Am-Uni +Cy5.5es	74.7/ 81.2	Operon
**T10**	ATACCCACACTATCTCGTCTAACACCCTGT AATTCCCTCGTATATTCGATCTATTATCTTAT ATCTAATTTTAATTATCGATTAATAGTCTTTT		75.4/ 81.7	IDT
**T11**	AACTAGCCGCACTAACTCGTGATACCCAC ACTATCTCGTCTATATTCGATCTATTATCTTA TATCTAATTTTAATTATCGATTAATAGTCTTTT		75.7/ 81.9	IDT
**T12**	CATTTACTATTTAATCTAACACACGAGTTAG TGCGGCTAGTTTATTCGATCTATTATCTTATA TCTAATTTTAATTATCGATTAATAGTCTTTT		74.7/ 81.1	IDT

Notes: Tm's are predicted values. All Cy3 and Cy5 dyes used where phosphoramidites. Unless otherwise indicated (ex. ester = es), the dyes are inserted as phosphoramidites.*in sequence indicates modifier placement.

Supplementary Table 23. DNA Sequences - 4-dye, 2-way, 1.5×R_0.

Name	Sequence	Modification	Tm (1X/ 2.5X PBS)	Source
**A**	*AAAAGACTATTAATCG	5' Cy3	43.5/ 48.8	IDT
**B**	ATAATT*AAAATTAGAT	Internal Am-Uni +Cy3.5es	35.5/ 41.7	Operon
**C**	ATAAGATAATAGATCGAATA*	3' Cy5	47.4/ 53.1	IDT
**D**	CATTTACTATTTAATCTAACA*	3' Cy5.5	48.5/ 54.3	IDT
**T13**	TACAGAAGTGTACAGTCAATGTTAGATTAA ATAGTAAATGTATTCGATCTATTATCTTATAT CTAATTTTAAATTATCGATTAATAGTCTTTT		74.0/ 80.6	IDT
**T14**	TTGACTGTACACTTCTGTATATTCGATCTA TTATCTTATATCTAATTTTAAATTATCGATT AATAGTCTTTT		69.7/ 76.2	IDT

Notes: Tm's are predicted values. All Cy3 and Cy5 dyes used where phosphoramidites. Unless otherwise indicated (ex. ester = es), the dyes are inserted as phosphoramidites.*in sequence indicates modifier placement.

Supplementary Table 24. DNA Sequences - 4-dye, 1-way, 1.5×R_0.

Name	Sequence	Modification	Tm (1X/ 2.5X PBS)	Source
**A**	*AAAAGACTATTAATCG	5' Cy3	43.5/ 48.8	IDT
**B**	ATAATT*AAAATTAGAT	Internal Am-Uni +Cy3.5es	35.5/ 41.7	Operon
**C**	ATAAGATAATAGATCGAATA*	3' Cy5	47.4/ 53.1	IDT
**D**	CATTTACTATTTAATCTAACA*	3' Cy5.5	48.5/ 54.3	IDT
**T15**	TGTTAGATTAAATAGTAAATGTATTCGATCT ATTATCTTATATCTAATTTTAATTATCGATTA ATAGTCTTTT		69.9/ 76.6	IDT

Notes: Tm's are predicted values. All Cy3 and Cy5 dyes used where phosphoramidites. Unless otherwise indicated (ex. ester = es), the dyes are inserted as phosphoramidites.*in sequence indicates modifier placement.

Supplementary Table 25. DNA Sequences - 4-dye, 8-way, 1.0×R_0.

Name	Sequence	Modification	Tm (1X/ 2.5X PBS)	Source
**A**	*ATCTTAATCGATA	5' Cy3	35.1/ 40.1	IDT
**B1**	*ATTAACATTAGA	5' Cy3.5	29.2/ 34.2	Operon
**C1**	TATAAGATCA*AG	Internal Cy5	31.4/ 36.4	IDT
**T1**	GCCGCAATCCT*CGCCTGCACCTATGATCTT ATATCTAATGTTAATTATCGATTAAGAT	Internal Am-Uni +Cy5.5es	74.1/ 79.8	Operon
**T2**	GTGCAGGCGATGAGCACGAGCTATGATCTT ATATCTAATGTTAATTATCGATTAAGAT		73.4/ 79.1	IDT
**T3**	CTCGTGCTCACCGAATGCCACTATGATCTTA TATCTAATGTTAATTATCGATTAAGAT		72.7/ 78.5	IDT
**T4**	TGGCATTCGGCGTCCAGCTCCTATGATCTTA TATCTAATGTTAATTATCGATTAAGAT		73.9/ 79.6	IDT
**T5**	GAGCTGGACGACAATGACGTCTATGATCTTA TATCTAATGTTAATTATCGATTAAGAT		71.9/ 77.7	IDT
**T6**	ACGTCATTGTACTATGGCACCTATGATCTTAT ATCTAATGTTAATTATCGATTAAGAT		71.3/ 77.3	IDT
**T7**	GTGCCATAGTGGTCAACGCACTATGATCTTAT ATCTAATGTTAATTATCGATTAAGAT		72.2/ 78.0	IDT
**T8**	TGCGTTGACCGGATTGCGGCCTATGATCTTAT ATCTAATGTTAATTATCGATTAAGAT		74.2/ 79.9	IDT

Notes: Tm's are predicted values. All Cy3 and Cy5 dyes used where phosphoramidites. Unless otherwise indicated (ex. ester = es), the dyes are inserted as phosphoramidites.*in sequence indicates modifier placement.

Supplementary Table 26. DNA Sequences - 4-dye, 4-way, 1.0×R_0.

Name	Sequence	Modification	Tm (1X/ 2.5X PBS)	Source
**A**	*ATCTTAATCGATA	5’ Cy3	35.0/ 40.1	IDT
**B1**	*ATTAACATTAGA	5’ Cy3.5	29.0/ 34.2	Operon
**C1**	TATAAGATCA*AG	Internal Cy5	29.9/ 34.8	IDT
**T9**	GTGTGATAGAT*GTATTCGATCTATGATCTT ATATCTAATGTTAATTATCGATTAAGAT	Internal Am-Uni +Cy5.5es	69.1/ 75.3	Operon
**T10**	ATCGAATACAATGTCCCACACTATGATCTT ATATCTAATGTTAATTATCGATTAAGAT		70.7/ 76.9	IDT
**T11**	TGTGGGACATAGCACAAGCTCTATGATCTT ATATCTAATGTTAATTATCGATTAAGAT		71.9/ 77.9	IDT
**T12**	AGCTTGTGCTTCTATCACACCTATGATCTT ATATCTAATGTTAATTATCGATTAAGAT		71.3/ 77.5	IDT

Notes: Tm’s are predicted values. All Cy3 and Cy5 dyes used where phosphoramidites. Unless otherwise indicated (ex. ester = es), the dyes are inserted as phosphoramidites.*in sequence indicates modifier placement.

Supplementary Table 27. DNA Sequences - 4-dye, 2-way, 1.0×R_0.

Name	Sequence	Modification	Tm (1X/ 2.5X PBS)	Source
**A**	*ATCTTAATCGATA	5’ Cy3	35.0/ 40.1	IDT
**B2**	*ATTAAAATTAGATATAAGATAA	5’ Cy3.5	45.8/ 51.9	Operon
**C2**	*TAGATCGAATACA	5’ Cy5	38.5/ 43.4	IDT
**T13**	*TTTACTATTGTAACATTTATCTTATATCTAA TTTTAATTATCGATTAAGAT	5’ Cy5.5	65.2/ 71.7	IDT
**T14**	*ATGTTACAATAGTAAATGTATTCGATCTATTA TCTTATATCTAATTTTAATTATCGATTAAGAT	5’ Cy5	68.2/ 74.8	IDT

Notes: Tm’s are predicted values. All Cy3 and Cy5 dyes used where phosphoramidites. Unless otherwise indicated (ex. ester = es), the dyes are inserted as phosphoramidites.*in sequence indicates modifier placement.

Supplementary Table 28. DNA Sequences - 4-dye, 1-way, 1.0×R_0.

Name	Sequence	Modification	Tm (1X/ 2.5X PBS)	Source
**A**	*ATCTTAATCGATA	5’ Cy3	35.0/ 40.1	IDT
**B2**	*ATTAAAATTAGATATAAGATAA	5’ Cy3.5	45.8/ 51.9	Operon
**C2**	*TAGATCGAATACA	5’ Cy5	38.5/ 43.4	IDT
**D**	*TTTACTATTTAATCT	5’ Cy5.5	36.2/ 41.8	IDT
**T15**	AGATTAAATAGTAAATGTATTCGATCTATTA TCTTATATCTAATTTTAATTATCGATTAAGAT		68.2/ 74.8	IDT

Notes: Tm’s are predicted values. All Cy3 and Cy5 dyes used where phosphoramidites. Unless otherwise indicated (ex. ester = es), the dyes are inserted as phosphoramidites.*in sequence indicates modifier placement.

Supplementary Table 29. DNA Sequences - 4-dye, 1-way, 1.0×R_0. Modification of Supplementary Table 28 with DNA from different source.

Name	Sequence	Modification	Tm (1X/ 2.5X PBS)	Source
**A**	*ATCTTAATCGATA	5' Cy3	35.0/ 40.1	Operon
**B2**	*ATTAAAATTAGATATAAGATAA	5' Cy3.5	45.8/ 51.9	Operon
**C2**	*TAGATCGAATACA	5' Cy5	38.5/ 43.4	IDT
**D**	*TTTACTATTTAATCT	5' Cy5.5	36.2/ 41.8	Operon
**T15**	AGATTAAATAGTAAATGTATTCGATCTATTA TCTTATATCTAATTTTAATTATCGATTAAGAT		68.2/ 74.8	IDT

Notes: Tm's are predicted values. All Cy3 and Cy5 dyes used where phosphoramidites. Unless otherwise indicated (ex. ester = es), the dyes are inserted as phosphoramidites.*in sequence indicates modifier placement. This is a modification of Table 34 with DNA from different source.

Supplementary Table 30. DNA Sequences - 4-dye, 8-way, 0.5×R_0.

Name	Sequence	Modification	Tm (1X/ 2.5X PBS)	Source
**A1**	ATAGTCTGCT*	3' Cy3	30.9/ 35.3	IDT
**B1**	AGCAGACTAT*TCGTCGC	Internal Am-Uni +Cy3.5es	58.7/ 63.3	Operon
**T1**	CGCAATCCT*GGCGAGCGCGACGA	Internal Am-Uni +Cy5.5es	75.0/ 79.3	Operon
**T2**	GC*CGCCATGAG*GCGGCGACGA	2 Internal Cy5	74.9/ 79.2	IDT
**T3**	CGCACTCAGCGAAAGCGCGACGA		73.1/ 77.6	IDT
**T4**	GC*TTCGCCCAC* AGCGCGACGA	2 Internal Cy5	74.1/ 78.5	IDT
**T5**	GCTAGTGGACACGACCGCGACGA		71.8/ 76.3	IDT
**T6**	GG*CGTGTACTG* GGCGCGACGA	2 Internal Cy5	73.4/ 77.6	IDT
**T7**	GCCACAGTCCTCAACGGCGACGA		72.5/ 77.0	IDT
**T8**	CG*TGAGGGGAT*GCGGCGACGA	2 Internal Cy5	73.2/ 77.3	IDT

Notes: Tm's are predicted values. All Cy3 and Cy5 dyes used where phosphoramidites. Unless otherwise indicated (ex. ester = es), the dyes are inserted as phosphoramidites.*in sequence indicates modifier placement.

Supplementary Table 31. DNA Sequences - 4-dye, 4-way, 0.5×R_0.

Name	Sequence	Modification	Tm (1X/ 2.5X PBS)	Source
**A1**	ATAGTCTGCT*	3' Cy3	30.9/ 35.3	IDT
**B1**	AGCAGACTAT*TCGTCGC	Internal Am-Uni +Cy3.5es	58.7/ 63.3	Operon
**T9**	GG*CACGAGGTG*CCAGCGACGA	Internal Cy5	73.5/ 77.7	IDT
**T10**	CCGATTGCT*CGTGACCGCGACGA	Internal Am-Uni +Cy5.5es	72.5/ 76.9	Operon
**T11**	GC*CGACCGCAA*CGGGCGACGA	Internal Cy5	76.1/ 80.1	IDT
**T12**	TGGACACCGGTCGAGCGCGACGA		74.2/ 78.5	IDT

Notes: Tm's are predicted values. All Cy3 and Cy5 dyes used where phosphoramidites. Unless otherwise indicated (ex. ester = es), the dyes are inserted as phosphoramidites.*in sequence indicates modifier placement.

Supplementary Table 32. DNA Sequences - 4-dye, 2-way, 0.5×R_0.

Name	Sequence	Modification	Tm (1X/ 2.5X PBS)	Source
**A2**	* AAAAGACTATTAATCGATAATTAAA	5' Cy3	53.2/ 59.3	IDT
**B2**	TAAT*AGTCTTTT	Internal Am-Uni +Cy3.5es	28.9/ 34.1	Operon
**B3**	TAGCTGCAT*AG	Internal Am-Uni +Cy3.5es	36.9/ 41.3	Operon
**C1**	TATCTTAT*TCTAATTTTAA*TATCGAT	2 Internal Cy5	52.9/59.0	IDT
**D1**	*ATTAGATATAAGATACTATGCAGCTA*	5' Cy5.5, 3' Cy3	57.0/ 62.8	IDT

Notes: Tm's are predicted values. All Cy3 and Cy5 dyes used where phosphoramidites. Unless otherwise indicated (ex. ester = es), the dyes are inserted as phosphoramidites.*in sequence indicates modifier placement.

Supplementary Table 33. DNA Sequences - 4-dye, 1-way, 0.5×R_0.

Name	Sequence	Modification	Tm (1X/ 2.5X PBS)	Source
**A2**	* AAAAGACTATTAATCGATAATTAAA	5' Cy3	53.2/ 59.3	IDT
**B2**	TAAT*AGTCTTTT	Internal Am-Uni +Cy3.5es	28.9/ 34.1	Operon
**C2**	TATCTTATATCTAATTTTAA*TATCGAT	Internal Cy5	52.9/ 59.0	IDT
**D2**	*ATTAGATATAAGATA	5' Cy5.5	343.9/ 39.4	IDT

Notes: Tm's are predicted values. All Cy3 and Cy5 dyes used where phosphoramidites. Unless otherwise indicated (ex. ester = es), the dyes are inserted as phosphoramidites.*in sequence indicates modifier placement.

Supplementary Table 34. DNA Sequences - 4-dye, 2:1 dendrimer, 0.5×R_0.

Name	Sequence	Modification	Tm (1X/ 2.5X PBS)	Source
**S1**	*TCGTTCCCTACAGGGTGT*	5' Cy3, 3' Cy3	62.3/66.9	IDT
**S2**	*TCAGACTCAGAAGTCGTT*	5' Cy3, 3' Cy3	57.9/62.1	IDT
**S3**	*TAGACGAGAAAGACGCAT*	5' Cy3, 3' Cy3	57.6/62.6	IDT
**S4**	*TGTACGACAACGTCCAGT*	5' Cy3, 3' Cy3	60.5/ 65.3	IDT
**M1**	ACTGGACGTT*ACGACCCAGAACGAGGGA ATT*AGGGAACGA	2 Internal Am-Uni +Cy3.5es	76.0/ 81.2	Operon
**M2**	ATGCGTCTTT*AGTGTGGGTAAGTCACGGG AT*TGAGTCTGA	2 Internal Am-Uni +Cy3.5es	74.3/ 79.7	Operon
**M1****	ACTGGACGT*ACGACCCAGAACGAGGGA AT*AGGGAACGA	2 Internal Cy3.5	76.2/ 81.2	Operon
**M2****	ATGTGTATT*GTGTGGGTAAGTCACGGG A*TGAGTCTGA	2 Internal Cy3.5	72.7/ 78.1	Operon
**L1**	AACGACTTCATCCCGTGACT*GCCGCACT AACTCGTG*TCTGGGTCGTATGTCGTACA	2 Internal Cy5	78.8/84.1	IDT
**L2**	ACACCCTGTAATTCCCTCGTACACGAGTT* AGTGCGGCATACCCACACTATCTCGTCTA	Internal Am-Uni +Cy5.5 es	78.0/83.3	Operon

Notes: Tm's are predicted values. All Cy3 and Cy5 dyes used where phosphoramidites. Unless otherwise indicated (ex. ester = es), the dyes are inserted as phosphoramidites.*in sequence indicates modifier placement.

Supplementary Table 35. DNA Sequences - 4-dye, 3:1 dendrimer, 0.5×R_0.

Name	Sequence	Modification	Tm (1X/ 2.5X PBS)	Source
**S5**	*TAGAAGAGGATGCACAT*	5' Cy3, 3' Cy3	53.9/ 59.0	IDT
**S6**	ATGTGCATCACAGGGAGT*	3' Cy3	60.9/65.7	IDT
**M3**	ACTCCCTGT*ACGACCCAGATACCCACAC TACTCTTCTA	Internal Am-Uni +Cy3.5es	73.1/78.4	Operon
**M4**	ACTCCCTGTT*AGTGTGGGTAAGTCACGGG AT*TCTCTTCTA	2 Internal Am-Uni +Cy3.5es	73.6/ 78.9	Operon
**L3**	ACTCCCTGTATCCCGTGAC*ACACGAGTT AGTGCGGCA*CTGGGTCGTATCTCTTCTA	2 Internal Cy 5	78.9/84.1	IDT
**L4**	ACTCCCTGTATCCCGTGAC*TAACTCGTG AACTCGTGTTCTGGGTCGTATCTCTTCTA	Internal Cy 5	77.1/82.5	IDT
**L5**	ACTCCCTGTATCCCGTGACTTGCCGCACT* CACGAGTTATCTGGGTCGTATCTCTTCTA	Internal Am-Uni +Cy 5.5es	78.4/ 83.7	Operon

Notes: Tm's are predicted values. All Cy3 and Cy5 dyes used where phosphoramidites. Unless otherwise indicated (ex. ester = es), the dyes are inserted as phosphoramidites.*in sequence indicates modifier placement.

Supplementary Table 36. DNA Sequences - 4-dye, 4:1 dendrimer, 0.5×R_0.

Name	Sequence	Modification	Tm (1X/ 2.5X PBS)	Source
**S5**	*TAGAAGAGGATGCACAT*	5' Cy3, 3' Cy3	53.9/ 59.0	IDT
**S7**	ATGTGCATCAGGGAACGA		61.2/ 66.0	IDT
**S8**	*TCGTTCCCTACAGGGAGT*	5' Cy3, 3' Cy3	61.7/ 66.3	IDT
**M4**	ACTCCCTGTT*AGTGTGGGTAAGTCACGGG AT*TCTCTTCTA	2 Internal Am-Uni +Cy3.5es	73.6/ 78.9	Operon
**M5**	ACTCCCTGTT*ACGACCCAGAACGAGGGAA TT*TCTCTTCTA	2 Internal Am-Uni +Cy3.5es	73.8/ 79.1	Operon
**M6**	ACTCCCTGTAATTCCCTCGTTACCCACACTAT CTCTTCTA		72.3/77.7	IDT
**L3**	ACTCCCTGTATCCCGTGAC*ACACGAGTT AGTGCGGCA*CTGGGTCGTATCTCTTCTA	2 Internal Cy 5	78.9/84.1	IDT
**L5**	ACTCCCTGTATCCCGTGACTTGCCGCACT* CACGAGTTATCTGGGTCGTATCTCTTCTA	Internal Am-Uni +Cy5.5es	78.4/ 83.7	Operon
**L6**	ACTCCCTGTATCCCGTGAC*TAACTCGTG CGGCTAGTT*CTGGGTCGTATCTCTTCTA	2 Internal Cy 5	78.0/83.2	IDT
**L7**	ACTCCCTGTATCCCGTGACTAACTAGCCGA ACTCGTGTTCTGGGTCGTATCTCTTCTA		77.5/82.9	IDT

Notes: Tm's are predicted values. All Cy3 and Cy5 dyes used where phosphoramidites. Unless otherwise indicated (ex. ester = es), the dyes are inserted as phosphoramidites.*in sequence indicates modifier placement.

Supplementary Table 37. DNA Sequences - 5-dye, 2:1 dendrimer, 0.5×R_0.

Name	Sequence	Modification	Tm (1X/ 2.5X PBS)	Source
**S9**	*GATGCACATTCGTTCCCT*	5’ AmC6-Alexa488,<br>3’ AmC7-Q-Alexa488	59.8/64.6	Operon
**M7**	AGGGAACGA*AGAAGAGACAGGGAG<br>*ATGTGCATC	2 Internal Cy3	7.23/77.5	IDT
**L8**	AGGGAACGAACTCCCTGTT*ACGACCCA<br>GAAGTCACGGGAT*TCTCTTCTAATGTGC<br>ATC	2 Internal Am-Uni<br>+Cy3.5es	77.8/83.2	Operon
**XL1**	AGGGAACGAACTCCCTGTATCCCGTGAC*<br>TAACTCGTGAGTGCGGCA*CTGGGTCGTA<br>TCTCTTCTAATGTGCATC	2 Internal Cy 5	79.7/85.0	IDT
**XL1**	AGGGAACGAACTCCCTGTATCCCGTGACT*<br>TAACTCGTGAGTGCGGCAT*CTGGGTCGTA<br>TCTCTTCTAATGTGCATC	2 Internal<br>AmC6-dT-Alexa 647	79.5/84.8	Operon
**XL2**	AGGGAACGAACTCCCTGTATCCCGTGACT<br>TGCCGCACT*CACGAGTTATCTGGGTCGTA<br>TCTCTTCTAATGTGCATC	Internal Am-Uni<br>+Cy5.5es	79.5/84.8	Operon

Notes: Tm’s are predicted values. All Cy3 and Cy5 dyes used where phosphoramidites. Unless otherwise indicated (ex. ester = es), the dyes are inserted as phosphoramidites.*in sequence indicates modifier placement.

Supplementary Table 38. Cy3-Cy5 FRET summary. Average donor energy losses, acceptor sensitized emission efficiencies, scaled photoluminescent intensity and end-to-end efficiency for the 2-dye constructs.

Configuration	Donor/ Acceptor	Donor Loss (%)	Acceptor Sensitization (%)	Scaled Cy5 PL Intensity[a]	End-to-end Efficiency (%)
**0.75×R_0**					
Unidirectional	1	64	74	0.325	40
Bidirectional	2	57	96	0.650	51
Holliday junction	4	51	68	1.000	37
8-way junction	8	25	28	0.731	15
**0.85×R_0**					
Unidirectional	1	50	41	0.155	22
Bidirectional	2	46	51	0.303	28
Holliday junction	4	38	56	0.543	30
8-way junction	8	18	30	0.573	16
**1.0×R_0**					
Unidirectional	1	24	33	0.082	18
Bidirectional	2	28	37	0.195	20
Holliday junction	4	31	43	0.499	23
8-way junction	8	22	28	0.516	15
**1.25×R_0**					
Unidirectional	1	12	16	0.066	9
Bidirectional	2	17	19	0.129	10
Holliday junction	4	7	17	0.277	9
8-way junction	8	7	14	0.300	8
**1.5×R_0**					
Unidirectional	1	8	6	0.016	3
Bidirectional	2	16	7	0.039	4
Holliday junction	4	6	6	0.065	4
8-way junction	8	4	4	0.064	2

[a]Within each data set, the decomposed Cy5 contribution to the PL area was normalized to the direct excitation PL of Cy5. The results were then all scaled by the maximum value of the decomposed Cy5 contribution. **Table corresponds to Figure 2C.**

Supplementary Table 39. Estimated formation efficiency for the Cy3-Cy5 2 dye structures.

	Structure	Formation percentage			Method
		full	partial	unformed	
**$1.5\times R_0$**	**linear/ bifurcated**	>90%	0%	0%	Gel electrophoresis
	**Holliday**	>90%	0%	0%	Gel electrophoresis
	**8 arm star**	~20%	~50%	~30%	Gel electrophoresis
**$1.25\times R_0$**	**linear/ bifurcated**	>90%	0%	0%	Gel electrophoresis
	**Holliday**	~60%	0%	~40%	Gel electrophoresis
	**8 arm star**	~40%	~30%	~30%	Gel electrophoresis
**$1.0 \times R_0$**	**linear/ bifurcated**	>90%	0%	0%	Gel electrophoresis
	**Holliday**	>90%	0%	0%	Gel electrophoresis
	**8 arm star**	~50%	~50%	0%	Gel electrophoresis
**$0.85 \times R_0$**	**linear/ bifurcated**	>90%	0%	0%	Gel electrophoresis
	**Holliday**	~40%	~10%	~50%	Gel electrophoresis
	**8 arm star**	~20%	~40%	~40%	Gel electrophoresis
**$0.75\times R_0$**	**Linear/ bifurcated**	>90%	0%	0%	Gel electrophoresis
	**Holiday**	~20%	~40%	~40%	Gel electrophoresis
	**8 arm star**	~10%	~40%	~50%	Gel electrophoresis

Notes: Estimates are ± 10%; fully formed structure: band present at the expected MW on the gel; partially formed structure: bands below fully formed, but not qualified as unformed; unformed structure: fluorescence present at <50 bp and consistent with ssDNA or unhybridized oligos.

Supplementary Table 40. 8-way, 1.5×R_0. Average donor energy loss, acceptor sensitized emission efficiency, and end-to-end efficiency for fluorophores in selected configurations.

Configuration	Cy3	Cy3.5	Cy5	Cy5.5	End-to-end Efficiency (%)
**Donor / Acceptor positions:**	**Donor loss**		**Acceptor emission**		
**1 2 3 4**					
Cy3 – Cy3.5	28%	4%			2%
Cy3 – Cy3.5 – Cy5	28%	100% (0%)	100%		3%
Cy3 – Cy3.5 – Cy5 – Cy5.5	41%	100% (0%)	55% (87%)	46%	2%
**Controls (1 missing)**					
Cy3 – sp – Cy5 – Cy5.5	14%	---	50% (3%)	28%	2%
Cy3 – Cy3.5 – sp – Cy5.5	37%	28% (3%)	---	10%	0%
sp – Cy3.5 – Cy5 – Cy5.5	---	23%	72% (4%)	32%	4%
**Controls (2 missing)**					
Cy3 – sp – Cy5 – sp	0%	---	6%		3%
Cy3 – sp – sp – Cy5.5	0%	---	---	0%	0%
sp – Cy3.5 – Cy5 – sp	---	19%	10%		4%
sp – Cy3.5 – sp – Cy5.5	---	0%	---	1%	0%
sp – sp – Cy5 – Cy5.5	---	---	15%	9%	8%

Supplementary Table 41. 4-way, 1.5×R_0. Average donor energy loss, acceptor sensitized emission efficiency, and end-to-end efficiency for fluorophores in selected configurations.

Configuration	Cy3	Cy3.5	Cy5	Cy5.5	End-to-end Efficiency (%)
**Donor / Acceptor positions:**	**Donor loss**		**Acceptor emission**		
**1 2 3 4**					
Cy3 – Cy3.5	19%	6%			5%
Cy3 – Cy3.5 – Cy5	19%	100% (0%)	98%		3%
Cy3 – Cy3.5 – Cy5 – Cy5.5	18%	100% (0%)	71% (29%)	70%	3%
**Controls (1 missing)**					
Cy3 – sp – Cy5 – Cy5.5	0%	---	98% (0%)	46%	2%
Cy3 – Cy3.5 – sp – Cy5.5	14%	21% (5%)	---	7%	1%
sp – Cy3.5 – Cy5 – Cy5.5	---	15%	80% (3%)	52%	5%
**Controls (2 missing)**					
Cy3 – sp – Cy5 – sp	0%	---	3%		1%
Cy3 – sp – sp – Cy5.5	0%	---	---	1%	1%
sp – Cy3.5 – Cy5 – sp	---	17%	9%		4%
sp – Cy3.5 – sp – Cy5.5	---	3%	---	1%	1%
sp – sp – Cy5 – Cy5.5	---	---	25%	20%	21%

Supplementary Table 42. 2-way, 1.5×R_0. Average donor energy loss, acceptor sensitized emission efficiency, and end-to-end efficiency for fluorophores in selected configurations.

Configuration	Cy3	Cy3.5	Cy5	Cy5.5	End-to-end Efficiency (%)
**Donor / Acceptor positions:**	**Donor loss**		**Acceptor emission**		
**1 2 3 4**					
Cy3 – Cy3.5	8%	4%			2%
Cy3 – Cy3.5 – Cy5	8%	54% (2%)	100%		2%
Cy3 – Cy3.5 – Cy5 – Cy5.5	8%	59% (2%)	57% (59%)	38%	1%
**Controls (1 missing)**					
Cy3 – sp – Cy5 – Cy5.5	0%	---	100% (0%)	26%	0%
Cy3 – Cy3.5 – sp – Cy5.5	7%	0% (4%)	---	18%	0%
sp – Cy3.5 – Cy5 – Cy5.5	---	6%	87% (1%)	38%	3%
**Controls (2 missing)**					
Cy3 – sp – Cy5 – sp	0%	---	1%		0%
Cy3 – sp – sp – Cy5.5	0%	---	---	0%	0%
sp – Cy3.5 – Cy5 – sp	---	7%	9%		3%
sp – Cy3.5 – sp – Cy5.5	---	1%	---	1%	0%
sp – sp – Cy5 – Cy5.5	---	---	26%	9%	11%

Supplementary Table 43. 1-way, 1.5×R_0. Average donor energy loss, acceptor sensitized emission efficiency, and end-to-end efficiency for fluorophores in selected configurations.

Configuration	Cy3	Cy3.5	Cy5	Cy5.5	End-to-end Efficiency (%)
**Donor / Acceptor positions:**	**Donor loss**	**Acceptor emission**			
**1 2 3 4**					
Cy3 – Cy3.5	7%	6%			6%
Cy3 – Cy3.5 – Cy5	2%	13% (5%)	76%		3%
Cy3 – Cy3.5 – Cy5 – Cy5.5	3%	42% (3%)	82% (14%)	42%	2%
**Controls (1 missing)**					
Cy3 – sp – Cy5 – Cy5.5	0%	---	100% (0%)	18%	1%
Cy3 – Cy3.5 – sp – Cy5.5	2%	0% (8%)	---	4%	1%
sp – Cy3.5 – Cy5 – Cy5.5	---	5%	92% (1%)	35%	2%
**Controls (2 missing)**					
Cy3 – sp – Cy5 – sp	0%	---	1%		1%
Cy3 – sp – sp – Cy5.5	0%	---	---	0%	1%
sp – Cy3.5 – Cy5 – sp	---	7%	6%		3%
sp – Cy3.5 – sp – Cy5.5	---	2%	---	1%	0%
sp – sp – Cy5 – Cy5.5	---	---	31%	11%	11%

Supplementary Table 44. 8-way, 1.0×R_0. Average donor energy loss, acceptor sensitized emission efficiency, and end-to-end efficiency for fluorophores in selected configurations.

Configuration	Cy3	Cy3.5	Cy5	Cy5.5	End-to-end Efficiency (%)
**Donor / Acceptor positions:**	**Donor loss**		**Acceptor emission**		
**1 2 3 4**					
Cy3 – Cy3.5	45%	27%			26%
Cy3 – Cy3.5 – Cy5	48%	100% (0%)	100%		19%
Cy3 – Cy3.5 – Cy5 – Cy5.5	47%	100% (0%)	26% (81%)	20%	6%
**Controls (1 missing)**					
Cy3 – sp – Cy5 – Cy5.5	0%	---	77% (6%)	7%	2%
Cy3 – Cy3.5 – sp – Cy5.5	44%	0% (32%)	---	5%	0%
sp – Cy3.5 – Cy5 – Cy5.5	---	29%	70% (12%)	11%	4%
**Controls (2 missing)**					
Cy3 – sp – Cy5 – sp	9%	---	6%		3%
Cy3 – sp – sp – Cy5.5	0%	---	---	1%	1%
sp – Cy3.5 – Cy5 – sp	---	37%	18%		8%
sp – Cy3.5 – sp – Cy5.5	---	0%	---	1%	0%
sp – sp – Cy5 – Cy5.5	---	---	13%	8%	0%

Supplementary Table 45. 4-way, 1.0×R_0. Average donor energy loss, acceptor sensitized emission efficiency, and end-to-end efficiency for fluorophores in selected configurations.

Configuration	Cy3	Cy3.5	Cy5	Cy5.5	End-to-end Efficiency (%)
**Donor / Acceptor positions:**	**Donor loss**		**Acceptor emission**		
**1 2 3 4**					
Cy3 – Cy3.5	37%	37%			37%
Cy3 – Cy3.5 – Cy5	39%	71% (11%)	53%		10%
Cy3 – Cy3.5 – Cy5 – Cy5.5	42%	81% (7%)	62% (20%)	73%	9%
**Controls (1 missing)**					
Cy3 – sp – Cy5 – Cy5.5	2%	---	86% (3%)	32%	4%
Cy3 – Cy3.5 – sp – Cy5.5	35%	20% (30%)	---	11%	3%
sp – Cy3.5 – Cy5 – Cy5.5	---	23%	84% (5%)	42%	7%
**Controls (2 missing)**					
Cy3 – sp – Cy5 – sp	3%	---	7%		3%
Cy3 – sp – sp – Cy5.5	38%	---	---	1%	1%
sp – Cy3.5 – Cy5 – sp	---	23%	18%		8%
sp – Cy3.5 – sp – Cy5.5	---	0%	---	3%	1%
sp – sp – Cy5 – Cy5.5	---	---	39%	23%	15%

Supplementary Table 46. 2-way, 1.0×R_0. Average donor energy loss, acceptor sensitized emission efficiency, and end-to-end efficiency for fluorophores in selected configurations.

Configuration	Cy3	Cy3.5	Cy5	Cy5.5	End-to-end Efficiency (%)
**Donor / Acceptor positions:**	**Donor loss**		**Acceptor emission**		
**1 2 3 4**					
Cy3 – Cy3.5	30%	44%			45%
Cy3 – Cy3.5 – Cy5	33%	55% (20%)	54%		13%
Cy3 – Cy3.5 – Cy5 – Cy5.5	36%	64% (16%)	77% (12%)	44%	7%
**Controls (1 missing)**					
Cy3 – sp – Cy5 – Cy5.5	0%	---	100% (0%)	11%	2%
Cy3 – Cy3.5 – sp – Cy5.5	32%	4% (42%)	---	6%	2%
sp – Cy3.5 – Cy5 – Cy5.5	---	22%	97% (1%)	27%	8%
**Controls (2 missing)**					
Cy3 – sp – Cy5 – sp	0%	---	3%		2%
Cy3 – sp – sp – Cy5.5	0%	---	---	1%	1%
sp – Cy3.5 – Cy5 – sp	---	19%	24%		12%
sp – Cy3.5 – sp – Cy5.5	---	0%	---	3%	1%
sp – sp – Cy5 – Cy5.5	---	---	57%	28%	24%

Supplementary Table 47. 1-way, 1.0×R_0. Average donor energy loss, acceptor sensitized emission efficiency, and end-to-end efficiency for fluorophores in selected configurations.

Configuration	Cy3	Cy3.5	Cy5	Cy5.5	End-to-end Efficiency (%)
**Donor / Acceptor positions:**	**Donor loss**		**Acceptor emission**		
**1 2 3 4**					
Cy3 – Cy3.5	26%	37%			33%
Cy3 – Cy3.5 – Cy5	22%	49% (19%)	62%		9%
Cy3 – Cy3.5 – Cy5 – Cy5.5	29%	58% (15%)	76% (15%)	36%	4%
**Controls (1 missing)**					
Cy3 – sp – Cy5 – Cy5.5	0%	---	100% (0%)	7%	1%
Cy3 – Cy3.5 – sp – Cy5.5	28%	0% (40%)	---	5%	1%
sp – Cy3.5 – Cy5 – Cy5.5	---	24%	93% (3%)	23%	4%
**Controls (2 missing)**					
Cy3 – sp – Cy5 – sp	0%	---	2%		1%
Cy3 – sp – sp – Cy5.5	0%	---	---	1%	0%
sp – Cy3.5 – Cy5 – sp	---	25%	21%		10%
sp – Cy3.5 – sp – Cy5.5	---	1%	---	2%	1%
sp – sp – Cy5 – Cy5.5	---	---	54%	27%	27%

Supplementary Table 48. 8-way, 0.5×R_0. Average donor energy loss, acceptor sensitized emission efficiency, and end-to-end efficiency for fluorophores in selected configurations.

Configuration	Cy3	Cy3.5	Cy5	Cy5.5	End-to-end Efficiency (%)
**Donor / Acceptor positions:**	**Donor loss**		**Acceptor emission**		
**1 2 3 4**					
Cy3 – Cy3.5	46%	100%			100%
Cy3 – Cy3.5 – Cy5	45%	73% (32%)	39%		17%
Cy3 – Cy3.5 – Cy5 – Cy5.5	49%	83% (22%)	34% (25%)	22%	6%
**Controls (1 missing)**					
Cy3 – sp – Cy5 – Cy5.5	0%	---	85% (6%)	8%	2%
Cy3 – Cy3.5 – sp – Cy5.5	42%	8% (100%)	---	4%	8%
sp – Cy3.5 – Cy5 – Cy5.5	---	46%	78% (11%)	9%	3%
**Controls (2 missing)**					
Cy3 – sp – Cy5 – sp	0%	---	8%		3%
Cy3 – sp – sp – Cy5.5	0%	---	---	2%	1%
sp – Cy3.5 – Cy5 – sp	---	45%	17%		7%
sp – Cy3.5 – sp – Cy5.5	---	15%	---	2%	4%
sp – sp – Cy5 – Cy5.5	---	---	18%	7%	1%

Supplementary Table 49. 4-way, 0.5×R_0. Average donor energy loss, acceptor sensitized emission efficiency, and end-to-end efficiency for fluorophores in selected configurations.

Configuration	Cy3	Cy3.5	Cy5	Cy5.5	End-to-end Efficiency (%)
**Donor / Acceptor positions:**	**Donor loss**	**Acceptor emission**			
**1 2 3 4**					
Cy3 – Cy3.5	56%	100%			100%
Cy3 – Cy3.5 – Cy5	64%	60% (45%)	12%		8%
Cy3 – Cy3.5 – Cy5 – Cy5.5	59%	83% (20%)	6% (12%)	87%	9%
**Controls (1 missing)**					
Cy3 – sp – Cy5 – Cy5.5	6%	---	86% (2%)	14%	2%
Cy3 – Cy3.5 – sp – Cy5.5	58%	22% (89%)	---	11%	10%
sp – Cy3.5 – Cy5 – Cy5.5	---	43%	69% (5%)	36%	4%
**Controls (2 missing)**					
Cy3 – sp – Cy5 – sp	0%	---	2%		1%
Cy3 – sp – sp – Cy5.5	0%	---	---	2%	1%
sp – Cy3.5 – Cy5 – sp	---	31%	4%		2%
sp – Cy3.5 – sp – Cy5.5	---	16%	---	6%	4%
sp – sp – Cy5 – Cy5.5	---	---	8%	3%	0%

Supplementary Table 50. 2-way, 0.5×R_0. Average donor energy loss, acceptor sensitized emission efficiency, and end-to-end efficiency for fluorophores in selected configurations.

Configuration	Cy3	Cy3.5	Cy5	Cy5.5	End-to-end Efficiency (%)
**Donor / Acceptor positions:**	**Donor loss**		**Acceptor emission**		
**1 2 3 4**					
Cy3 – Cy3.5	37%	62%			61%
Cy3 – Cy3.5 – Cy5	69%	100% (0%)	100%		37%
Cy3 – Cy3.5 – Cy5 – Cy5.5	78%	100% (0%)	94% (7%)	34%	14%
**Controls (1 missing)**					
Cy3 – sp – Cy5 – Cy5.5	31%	---	99% (1%)	12%	5%
Cy3 – Cy3.5 – sp – Cy5.5	57%	96% (3%)	---	9%	5%
sp – Cy3.5 – Cy5 – Cy5.5	---	73%	100% (0%)	12%	9%
**Controls (2 missing)**					
Cy3 – sp – Cy5 – sp	6%	---	17%		10%
Cy3 – sp – sp – Cy5.5	23%	---	---	7%	5%
sp – Cy3.5 – Cy5 – sp	---	53%	44%		24%
sp – Cy3.5 – sp – Cy5.5	---	14%	---	1%	1%
sp – sp – Cy5 – Cy5.5	---	---	65%	8%	4%

Supplementary Table 51. 1-way, 0.5×R_0. Average donor energy loss, acceptor sensitized emission efficiency, and end-to-end efficiency for fluorophores in selected configurations.

Configuration	Cy3	Cy3.5	Cy5	Cy5.5	End-to-end Efficiency (%)
**Donor / Acceptor positions:**	**Donor loss**		**Acceptor emission**		
**1 2 3 4**					
Cy3 – Cy3.5	64%	56%			55%
Cy3 – Cy3.5 – Cy5	75%	92% (5%)	100%		45%
Cy3 – Cy3.5 – Cy5 – Cy5.5	73%	75% (14%)	85% (23%)	28%	16%
**Controls (1 missing)**					
Cy3 – sp – Cy5 – Cy5.5	11%	---	99% (1%)	10%	5%
Cy3 – Cy3.5 – sp – Cy5.5	70%	30% (39%)	---	25%	10%
sp – Cy3.5 – Cy5 – Cy5.5	---	34%	99% (1%)	9%	12%
**Controls (2 missing)**					
Cy3 – sp – Cy5 – sp	16%	---	23%		13%
Cy3 – sp – sp – Cy5.5	0%	---	---	2%	1%
sp – Cy3.5 – Cy5 – sp	---	41%	61%		32%
sp – Cy3.5 – sp – Cy5.5	---	18%	---	10%	6%
sp – sp – Cy5 – Cy5.5	---	---	73%	23%	16%

Supplementary Table 52. 4-dye FRET summary. Average donor energy losses, acceptor sensitized emission efficiencies, end-to-end efficiency, and scaled photoluminescent intensity for fluorophores in various constructs.

**Configuration**	**Cy3**		**Cy3.5**		**Cy5**		**Cy5.5**	
**0.5×R_0**								
1-way	73%[a]	0.058[b]	75% (14%)[c]	0.029	85% (23%)	0.027	**16%**[d] (28%)	0.051
2-way	78%	0.092	100% (0%)	0.000	94% (7%)	0.018	**14%** (34%)	0.094
4-way	59%	0.350	83% (20%)	0.171	6% (12%)	0.113	**9%** (87%)	0.105
8-way	49%	0.600	83% (22%)	0.275	34% (25%)	0.470	**6%** (22%)	0.129
**1.0×R_0**								
1-way	29%	0.150	58% (15%)	0.033	76% (15%)	0.012	**4%** (36%)	0.017
2-way	36%	0.272	64% (16%)	0.067	77% (12%)	0.022	**7%** (44%)	0.044
4-way	42%	0.494	81% (7%)	0.059	62% (20%)	0.064	**9%** (73%)	0.123
8-way	47%	0.899	100% (0%)	0.000	26% (81%)	0.366	**6%** (20%)	0.096
**1.5×R_0**								
1-way	3%	0.207	42% (3%)	0.008	82% (14%)	0.002	**2%** (42%)	0.004
2-way	8%	0.390	59% (2%)	0.007	57% (59%)	0.007	**1%** (38%)	0.006
4-way	18%	0.693	100% (0%)	0.000	71% (29%)	0.014	**3%** (70%)	0.033
8-way	41%	1.000	100% (0%)	0.000	55% (87%)	0.046	**2%** (46%)	0.046

[a]Average donor loss

[b]Average scaled and normalized PL area of each fluorophore. PL area for each fluorophore was determined by decomposition of PL spectra collected from full Cy3-Cy5.5 construct. Across all data sets and constructs, the fluorophore contributions were then normalized to the direct excitation from molar equivalent Cy3 controls and scaled based on the construct (uni-, bi-directional, Holliday or 8-way junction). The resulting values for the full constructs were then all scaled by the maximum value (1.5R0 8-way junction)

[c]Parenthetical values are average acceptor sensitized emission efficiencies

[d]Average end-to-end efficiency through the three-step Cy3-Cy3.5-Cy5-Cy5.5 construct.

Supplementary Table 53. Estimated formation efficiency for the 4-/5-dye structures.

	Structure	Formation percentage			Method
		full	partial	unformed	
**$1.5\times R_0$**	**linear**	~60%	~40%	~0%	Gel electrophoresis
	**bifurcated**	~60%	~20%	~20%	Gel electrophoresis
	**Holliday**	~60%	~30%	~10%	Gel electrophoresis
	**8 arm star**	~30%	~60%	~10%	Gel electrophoresis
**$1.0\times R_0$**	**linear**	>90%	0%	0%	Gel electrophoresis
	**bifurcated**	~80%	~20%	~0%	Gel electrophoresis
	**Holliday**	~40%	~60%	~0%	Gel electrophoresis
	**8 arm star**	~20%	~70%	~10%	Gel electrophoresis
**$0.5\times R_0$**	**linear**	>90%	0%	0%	Gel electrophoresis
	**bifurcated**	>90%	0%	0%	Gel electrophoresis
	**Holliday**	~20%	~40%	~40%	Gel electrophoresis
	**8 arm star**	~20%	~50%	~30%	Gel electrophoresis
	**2:1 dendrimer**	~70%	0%	~30%	FPLC
	**3:1 dendrimer**	~20%	~60%	~20%	FPLC
	**4:1 dendrimer**	~10%	~60%	~30%	FPLC
	**5 step 2:1 dendrimer**	~60%	0%	~40%	FPLC

Notes: Estimates are ± 10%; fully formed structure: band present at the expected MW on the gel; partially formed structure: bands below fully formed, but not qualified as unformed; unformed structure: fluorescence present at <50 bp and consistent with ssDNA or unhybridized oligos.

Supplementary Table 54. 0.5×R_0 2:1 dendrimer. Average donor energy loss, acceptor sensitized emission efficiency, and end-to-end efficiency for fluorophores in selected configurations.

Configuration	Cy3	Cy3.5	Cy5	Cy5.5	End-to-end Efficiency (%)
**Donor / Acceptor positions:**	**Donor loss**	**Acceptor emission**			
**1 2 3 4**					
Cy3 – Cy3.5	57%	62%			85%
Cy3 – Cy3.5 – Cy5	63%	99% (1%)	73%		34%
Cy3 – Cy3.5 – Cy5 – Cy5.5	64%	100% (0%)	77% (16%)	53%	17%
**Controls (1 missing)**					
Cy3 – sp – Cy5 – Cy5.5	47%	---	89% (5%)	28%	9%
Cy3 – Cy3.5 – sp – Cy5.5	55%	65% (22%)	---	32%	19%
sp – Cy3.5 – Cy5 – Cy5.5	---	73%	98% (1%)	11%	7%
**Controls (2 missing)**					
Cy3 – sp – Cy5 – sp	39%	---	24%		17%
Cy3 – sp – sp – Cy5.5	26%	---	---	8%	8%
sp – Cy3.5 – Cy5 – sp	---	71%	20%		14%
sp – Cy3.5 – sp – Cy5.5	---	59%	---	8%	7%
sp – sp – Cy5 – Cy5.5	---	---	83%	19%	3%
sp - spacer					

Supplementary Table 55 0.5×R_0 3:1 dendrimer. Average donor energy loss, acceptor sensitized emission efficiency, and end-to-end efficiency for fluorophores in selected configurations.

Configuration	Cy3	Cy3.5	Cy5	Cy5.5	End-to-end Efficiency (%)
**Donor / Acceptor positions:**	**Donor loss**		**Acceptor emission**		
**1 2 3 4**					
Cy3 – Cy3.5	61%	100%			100%
Cy3 – Cy3.5 – Cy5	72%	100% (0%)	82%		54%
Cy3 – Cy3.5 – Cy5 – Cy5.5	70%	84% (20%)	63% (30%)	34%	23%
**Controls (1 missing)**					
Cy3 – sp – Cy5 – Cy5.5	32%	---	80% (19%)	22%	13%
Cy3 – Cy3.5 – sp – Cy5.5	65%	43% (68%)	---	26%	20%
sp – Cy3.5 – Cy5 – Cy5.5	---	61%	91% (16%)	9%	10%
**Controls (2 missing)**					
Cy3 – sp – Cy5 – sp	100%	---	0%		0%
Cy3 – sp – sp – Cy5.5	14%	---	---	16%	10%
sp – Cy3.5 – Cy5 – sp	---	64%	38%		20%
sp – Cy3.5 – sp – Cy5.5	---	35%	---	14%	9%
sp – sp – Cy5 – Cy5.5	---	---	26%	30%	28%
sp - spacer					

Supplementary Table 56. 0.5×R_0 4:1 dendrimer. Average donor energy loss, acceptor sensitized emission efficiency, and end-to-end efficiency for fluorophores in selected configurations.

Configuration	Cy3	Cy3.5	Cy5	Cy5.5	End-to-end Efficiency (%)
**Donor / Acceptor positions:**	**Donor loss**	**Acceptor emission**			
**1 2 3 4**					
Cy3 – Cy3.5	50%	67%			70%
Cy3 – Cy3.5 – Cy5	57%	88% (8%)	71%		25%
Cy3 – Cy3.5 – Cy5 – Cy5.5	57%	100% (0%)	26% (53%)	20%	8%
**Controls (1 missing)**					
Cy3 – sp – Cy5 – Cy5.5	12%	---	72% (13%)	11%	4%
Cy3 – Cy3.5 – sp – Cy5.5	51%	22% (52%)	---	11%	4%
sp – Cy3.5 – Cy5 – Cy5.5	---	60%	74% (19%)	10%	6%
**Controls (2 missing)**					
Cy3 – sp – Cy5 – sp	6%	---	15%		8%
Cy3 – sp – sp – Cy5.5	0%	---	---	4%	3%
sp – Cy3.5 – Cy5 – sp	---	51%	26%		14%
sp – Cy3.5 – sp – Cy5.5	---	26%	---	6%	4%
sp – sp – Cy5 – Cy5.5	---	---	27%	9%	0%

sp - spacer

Supplementary Table 57. 5-dye (AF488-Cy3-Cy3.5-Cy5-Cy5.5), 0.5×R_0 2:1 dendrimer. Average donor energy loss, acceptor sensitized emission efficiency, and end-to-end efficiency for fluorophores in selected configurations.

Configuration	AF488	Cy3	Cy3.5	Cy5	Cy5.5	End-to-end Efficiency (%)
**Donor / Acceptor positions:**	**Donor loss**	**Acceptor emission**				
**1 2 3 4 5**						
AF488 – Cy3	69%	26%				66%
AF488 – Cy3 – Cy3.5	76%	70% (8%)	100%			89%
AF488 – Cy3 – Cy3.5 – Cy5	75%	75% (6%)	54% (62%)	53%		26%
AF488 – Cy3 – Cy3.5 – Cy5– Cy5.5	73%	71% (7%)	69% (42%)	72% (15%)	60%	19%
**Controls (1 missing)**						
sp – Cy3 – Cy3.5 – Cy5– Cy5.5	---	75%	93% (10%)	95% (3%)	12%	15%
AF488 – sp – Cy3.5 – Cy5– Cy5.5	35%	---	82% (6%)	86% (8%)	35%	11%
AF488 – Cy3 – sp – Cy5– Cy5.5	66%	0% (26%)	---	90% (7%)	34%	11%
AF488 – Cy3 – Cy3.5 – sp – Cy5.5	75%	73% (7%)	42% (78%)	---	1%	14%
**Controls (2 missing)**						
sp – sp – Cy3.5 – Cy5– Cy5.5	---	---	98%	99% (1%)	2%	20%
AF488 – sp – sp – Cy5– Cy5.5	10%	---	---	96% (2%)	11%	4%
AF488 – Cy3 – sp – sp – Cy5.5	69%	0% (33%)	---	---	13%	6%
sp - spacer						

Supplementary Table 58. 5-dye (AF488-Cy3-Cy3.5-AF647-Cy5.5), 0.5×R_0 2:1 dendrimer. Average donor energy loss, acceptor sensitized emission efficiency, and end-to-end efficiency for fluorophores in selected configurations.

Configuration	AF488	Cy3	Cy3.5	Cy5	Cy5.5	End-to-end Efficiency (%)
**Donor / Acceptor positions:**	**Donor loss**	**Acceptor emission**				
**1 2 3 4 5**						
AF488 – Cy3	62%	30%				76%
AF488 – Cy3 – Cy3.5	68%	73% (8%)	100%			86%
AF488 – Cy3 – Cy3.5 – AF647	68%	76% (7%)	47% (60%)	48%		23%
AF488 – Cy3 – Cy3.5 – AF647– Cy5.5	66%	74% (8%)	69% (35%)	66% (16%)	57%	16%
**Controls (1 missing)**						
sp – Cy3 – Cy3.5 – AF647– Cy5.5	---	59%	95% (6%)	97% (2%)	7%	9%
AF488 – sp – Cy3.5 – AF647– Cy5.5	32%	---	83% (6%)	83% (8%)	30%	8%
AF488 – Cy3 – sp – AF647– Cy5.5	61%	10% (27%)	---	92% (4%)	33%	9%
AF488 – Cy3 – Cy3.5 – sp – Cy5.5	70%	76% (7%)	45% (62%)	---	1%	14%
**Controls (2 missing)**						
sp – sp – Cy3.5 – AF647– Cy5.5	---	---	98%	99% (1%)	2%	16%
AF488 – sp – sp – AF647– Cy5.5	9%	---	---	96% (2%)	9%	3%
AF488 – Cy3 – sp – sp – Cy5.5	64%	0% (30%)	---	---	12%	6%
sp - spacer						

Supplementary Table 59. 0.5×R_0 2:1 dendrimer with substituted Cy3.5 phosphoramidite dye. Average donor energy loss, acceptor sensitized emission efficiency, and end-to-end efficiency for fluorophores in selected configurations.

Configuration	Cy3	Cy3.5	Cy5	Cy5.5	End-to-end Efficiency (%)
**Donor / Acceptor positions:**	**Donor loss**		**Acceptor emission**		
**1 2 3 4**					
Cy3 – Cy3.5	59%	86%			86%
Cy3 – Cy3.5 – Cy5	58%	100% (0%)	100%		52%
Cy3 – Cy3.5 – Cy5 – Cy5.5	61%	100% (0%)	66% (36%)	43%	28%
**Controls (1 missing)**					
Cy3 – sp – Cy5 – Cy5.5	33%	---	88% (11%)	19%	11%
Cy3 – Cy3.5 – sp – Cy5.5	66%	65% (33%)	---	42%	23%
sp – Cy3.5 – Cy5 – Cy5.5	---	83%	89% (29%)	12%	21%
**Controls (2 missing)**					
Cy3 – sp – Cy5 – sp	34%	---	30%		15%
Cy3 – sp – sp – Cy5.5	22%	---	---	10%	6%
sp – Cy3.5 – Cy5 – sp	---	79%	77%		40%
sp – Cy3.5 – sp – Cy5.5	---	40%	---	27%	17%
sp – sp – Cy5 – Cy5.5	---	---	40%	22%	17%
sp - spacer					

Supplementary Table 60. Distances between dye attachment points in the various structures used in this study.

(a)

Cy3-Cy5	$0.75R_0$	$0.85R_0$	$0.99R_0$	$1.25R_0$	$1.46R_0$
**r** (Å)	35/39	49/51	54/58	67/71	83/85

(b)

r (Å)	$0.5R_0$			$1.0R_0$			$1.5R_0$		
	Cy3-Cy3.5	Cy3.5-Cy5	Cy5-Cy5.5	Cy3-Cy3.5	Cy3.5-Cy5	Cy5-Cy5.5	Cy3-Cy3.5	Cy3.5-Cy5	Cy5-Cy5.5
1	36	38	21	51	75	46	75	102	75
2	36	38	21	51	75	46	75	105	71
4	39	34/42	17	51	75	46	75	105	71
8	39	34/42	17	51	75	46	75	105	75

(c)

branch ratio	r(Å), $0.5R_0$			
	Alexa488-Cy3	Cy3-Cy3.5	Cy3.5-Cy5	Cy5 or A647-Cy5.5
2:1	31	34/39	38/42	31/36
3:1	---	34	38	31
4:1	---	55	55	41

Note: When multiple values are given, this is because a particular structure has arms that differ in length, generally by a single base pair. These differences were included in the simulations.

Supplementary Table 61. Scaled generation rates. Computed for (**a**) two-dye star structures, (**b**) the multi-dye star structures and (**c**) the dendrimers with varying branching ratios.

(a)

$\Psi(10^7)$	$0.75R_0$	$0.85R_0$	$1.0R_0$	$1.25R_0$	$1.5R_0$
1	0.27	0.36	0.23	0.26	0.23
2	0.41	0.60	0.47	0.46	0.48
4	0.85	0.92	0.95	1.05	0.86
8	1.79	1.84	1.71	1.61	1.57

(b)

$\Psi(10^7)$	$0.5R_0$	$1.0R_0$	$1.5R_0$
1	2.47	3.32	2.57
2	1.96	2.78	2.30
4	1.04	1.13	1.08
8	1.81	2.41	2.58

(c)

$\Psi(10^7)$	$0.5R_0$ (4 dye)	$0.5R_0$ (5 dye, Cy5)	$0.5R_0$ (5 dye, A647)
2:1	0.41		
3:1	1.23	---	---
4:1	1.84	---	---

Supplementary Table 62. The direct generation rates as computed for the star-pair structures.

$N_{Cy3}\eta_{Cy3}$ (%)	$0.75R_0$	$0.85R_0$	$0.99R_0$	$1.25R_0$	$1.46R_0$
1	97.9	97.5	96.3	97.8	96.4
2	98.6	98.4	98.2	98.6	98.4
4	99.4	99.0	99.2	99.3	99.1
8	99.6	99.5	99.5	99.5	99.5

Supplementary Table 63. The direct generation rates as computed for the multi-dye stars.

$N_m\eta_m$ (%)	$0.5R_0$			$1.0R_0$			$1.5R_0$		
	Cy3	Cy3.5	Cy5	Cy3	Cy3.5	Cy5	Cy3	Cy3.5	Cy5
1	74.3	25.7	-	65.3	34.7	-	69.5	30.5	-
2	65.0	35.0	-	68.8	31.2	-	74.4	25.6	-
4	53.5	46.5	-	63.2	36.8	-	72.4	27.6	-
8	52.7	47.3	-	58.4	41.6	-	74.6	25.4	-
1	73.1	25.2	1.7	64.2	34.1	1.7	68.2	30.0	1.8
2	64.4	34.6	1.0	67.6	30.8	1.6	72.8	25.0	2.2
4	53.0	46.1	0.9	62.0	36.2	1.8	72.4	26.8	0.8
8	52.2	46.9	0.9	57.4	40.9	1.7	74.6	24.9	0.5
1	72.6	25.1	1.7	63.9	33.9	1.7	67.7	29.8	1.9
2	64.1	34.4	1.0	66.8	30.4	1.7	72.4	24.9	2.1
4	52.3	45.6	0.9	60.7	35.4	1.7	69.6	26.3	2.0
8	51.3	46.1	0.9	55.6	39.6	1.7	71.4	24.3	1.9

Supplementary Table 64. The direct generation rates as computed for the dendrimers with varying branching ratios.

$N_m\eta_m$ (%)	$0.5R_0$, 4 dye		
	Cy3	Cy3.5	Cy5
2:1	69.2	30.8	-
3:1	66.3	33.7	-
4:1	61.2	38.8	-
2:1	68.5	30.6	0.9
3:1	66.1	33.4	0.5
4:1	61.0	38.7	0.3
2:1	68.2	30.4	0.9
3:1	65.9	33.5	0.5
4:1	60.9	38.6	0.3

$N_m\eta_m$ (%)	2:1, $0.5R_0$, 5 dye			
	A488	Cy3	Cy3.5	Cy5 or A647
w/ Cy5	69.2	30.8	-	-
	68.5	30.6	0.9	-
	68.2	30.4	0.9	

Supplementary Table 65. Predicted DNA end-to-end length (bp) / total ds-base pairs for the 2-dye structures.

Structure ($\times R_0$)	0.75	0.85	1.0	1.25	1.5
**8-arm**	18/72	28/112	32/128	38/152	48/192
**Holliday**	18/36	28/56	32/64	38/76	48/96
**Bifurcated**	18	28	32	38	48
**Linear**	18	28	32	38	48

Supplementary Table 66. Predicted DNA end-to-end length (bp)/ total ds-base pairs for the 4/5-dye structures.

Structure	$0.5\times R_0$	$1.0\times R_0$	$1.5\times R_0$
**8-arm**	24/208	58/380	94/600
**Holliday**	24/104	58/190	94/300
**Bifurcated**	52	98	144
**Linear**	40	63	73
**2:1**	58/134		
**3:1**	58/369		
**4:1**	58/788		
**2:1 (5-dye)**	76/278		

Supplementary Table 67. Representative base separation calculations.

Base separation	**1**	**2**	**3**	**4**	**5**	**6**	**7**	**8**	**9**	**10**	**11**
Opposite side (Å)	33	30	25	20	18	21	28	36	42	48	51
Same side (Å)	11	20	28	34	38	39	38	35	33	35	38

Supplementary Note 1: DNA design

The DNA designs used in this study to organize the dyes were built of double-stranded segments, each separating a dye-pair, and with lengths chosen to put the dye spacing as close as possible to a particular fixed fraction of the Förster distance (*R_0*) for that pair, *e.g.*, 0.5, 1.0 or 1.5xR_0 for the [Cy3→Cy3.5→Cy5]$_n$→Cy5.5 photonic wire series. To estimate these lengths, the R_0 value associated with any given donor *i* and acceptor *j* was computing using the standard formula:[1]

$$R_0^{ij} = 9780\left[\frac{\kappa^2 J_{ij} Q_i}{n^4}\right]^{1/6} \tag{1}$$

where J_{ij} is the spectral overlap integral (estimated from the emission spectrum of donor *i*, the absorption spectrum of acceptor *j*, and the molar extinction coefficients), *n* is the refractive index of the medium, Q_i is the fluorescence quantum yield (QY) of the donor *i*, and κ^2 is the dipole orientation factor that is usually taken to be 2/3 as is appropriate for the quasi-random dipole orientations found in these constructs (see below).[1]. The values for R_0 obtained using (1) appear in Table 1. To obtain rough approximations to the nearest-neighbor dye separations we exploit the fact that each such dye pair is separated/supported by a DNA duplex that is much shorter than the persistence length and so can be regarded as straight/rigid. The distance between the dyes, or more precisely between the dye attachment points, can then be arranged by the DNA design as estimated by the cylinder model [2] in Å as:

$$a_{ij} = \sqrt{(3.4N_{ij})^2 + 2d^2\left[1-\cos(\phi_{ij} + 34.3N_{ij})\right]} \tag{2}$$

where N_{ij} is the number of intervening bases, ϕ_{ij} is either 0 or 180° depending on whether the dyes are attached to the same DNA strand or to the complementary strand, *d* ~ 1 nm is the half-width of the DNA helix, and the factor of 34.3 comes from the 360° of rotation divided by the number of bases in a full turn. Dye attachment distances calculated in this way for the various DNA designs of this paper are shown in Supplementary Table 60.

The dyes were attached to the DNA both at terminal positions and internally. The former occurred at ends, nicks, or breaks in the DNA that were introduced no less than 9 bases apart (for reasons of stability), and that put the attachment points at the 3’ (preferred) and/or 5’ ends of a DNA strand. Where possible, the designs used a long scaffold strand (template) to which the

dyes were attached via hybridization of shorter oligos, however, for the closest dye spacing's (*i.e.*, $0.5 \times R_0$) a staggered or concatenated DNA construction approach (no template) had to be used. To check for excessive distortions at junctions, all of the DNA designs (without dyes) were constructed within the program Nanoengineer[3]. Specific considerations regarding the attachment of the individual dyes are as follows:

The Cy5 dye is the most tightly bound to the DNA scaffold because it is incorporated directly into a DNA strand as a phosphoramidite (with an unpaired adenine in the opposite strand) and is always an internal label that thus has two attachment points as shown in Supplementary Figure 32C. The Cy5 dyes can therefore be expected to sit parallel and very close to the DNA scaffold, and without much flexibility.

The Cy3 dye is also attached as phosphoramidite, but unlike the Cy5, it is usually situated at a terminal position as shown in Supplementary Figure 32C with a single-point of attachment. (The one exception is in the 5-dye dendrimer where the Cy3s are internal and are then attached in an identical manner to the Cy5s). This will most likely result in more flexibility in both position and orientation (although there is evidence from molecular dynamics that it could stack onto the end of the duplex[4,5,6]). We therefore assume the terminal Cy3 dyes will tend to be directed away from the end of the DNA, and counting the size of the elongated molecule, will add an extra 5-10Å to the spacing. The flexibility of the attachment means the dye's precise orientation will vary, thus justifying the random dipole approximation.

The Cy3.5 dye is also an internal label, but it is not a phosphoramidite and instead has the dye more loosely attached via a C_6 linker following succinimidyl ester conjugation. Given its internal position, we assume it will tend to be radially directed and with a distance from the helical axis on the order of 10Å. Again, its flexibility in orientation supports the use of the random dipole approximation. The one exception to this is the 4-dye 2:1 dendrimer structure where Cy3.5 ester was replaced with a phosphoramidite (Figure 3H).

The attachment of Cy5.5 is the same as for the Cy3.5, however, it sometimes is an internal label and sometimes a terminal label. In the former case, its positioning is assumed mainly radial, whereas for the latter a preference for an axial extension is assumed. The A488 dye is a terminal

label with a flexible C_6 linker and an assumed axial preference. The A647 dye has the same attachment as the A488, but is internal and has an assumed radial preference.

The particular base sequences used for the designs of this paper are given in Supplementary Tables 6-42, with those for the "star" geometries adapted from Spillmann *et al.* [7] or from Wang and Seeman[8], while the dendrimer sequences were designed *de novo*. To ensure stability the melt temperatures (T_m) were checked using Oligo Analyzer (https://www.idtdna.com/pages/scitools) and the values for the final designs are also listed in Supplementary Tables 6-42. For an acceptable sequence, the melt temperatures of undesired pairings were required to be less than 30°C at a salt concentration corresponding to 2.5X PBS buffer. These sequences were also checked for self-complementarity and cross-complementarity using Operon's Oligo Analysis Tool, with a limit imposed of no more than 5 bases of non-specific complementarity.

The first system considered had *n* Cy3 donors assembled around a Cy5 acceptor with *n* systematically increased in the linear (*n* = 1), bifurcated (*n* = 2), Holliday junction (*n* = 4) and star (*n* = 8) configurations. A variety of such structures were made having donor-acceptor spacing's of approximately $0.75R_0$, $0.85R_0$, $1.0R_0$, $1.25R_0$, and $1.5R_0$ where R_0 ~ 54Å is the Förster distance for the Cy3-Cy5 pair. The second system considered had a configuration of $[\text{Cy3}\rightarrow\text{Cy3.5}\rightarrow\text{Cy5}]_n\rightarrow$Cy5.5 and was designed in a similar fashion, albeit with a 4 dye cascade, where *n* again equals 1, 2, 4, or 8. Given the added complexity, these structures were implemented with only three inter-dye distances, namely 0.5, 1.0 and $1.5\times R_0$, with the value of R_0 varying within each structure according to the particular dye-pairs involved. As already noted, care must be taken in the designs in order to accommodate the dyes. The last set of DNA structures employed dendrimeric designs in which each acceptor is fed by multiple donors in configurations of $[[\text{Cy3}_n\rightarrow\text{Cy3.5}]_n\rightarrow\text{Cy5}]_n\rightarrow$Cy5.5, where *n* equals 2, 3, or 4. Another version of this design involved 5 stages, and with *n* restricted to 2, in the configuration $[[[\text{A488}_2\rightarrow\text{Cy3}]_2\rightarrow\text{Cy3.5}]_2\rightarrow\text{Cy5/A647}]_2\rightarrow$Cy5.5. In all cases, these multi-generational structures were implemented using a branching motif that provided each internal dye with *n*+1 arms connecting *n* donor inputs to one acceptor output. Given the complexity of these structures only the $0.5\times R_0$ spacing was designed and assembled. The structures were designed with 2 long 58 base oligos, one of which was internally labeled at the center with a Cy5.5 and the other had 2

internal Cy5 labels that occur at the branching junction. 40 base oligos were assembled to the 58 base center and doubly internally labeled with Cy3.5. Finally, at the ends were 18 base oligos with 3' and 5' labeled Cy3s.

Supplementary Note 2: Figures-of-merit/metrics

Viewing the fluorophore assemblies of this paper as light-harvesters, it is of central interest to assess their overall performance. To this end, we employ the following metrics or figures-of-merit many of which can be estimated from experiment and all of which can be calculated from the Förster analysis. The latter also allows one to assess ideal performance, and to address questions of mechanism and of how to improve performance.

The terminal enhancement factor (TEF) is a relative measure of performance that compares the output of the terminal acceptor of each construct to that of a reference construct:

$$TEF = \frac{\Phi_A}{\Phi_{A,ref}} \tag{3a}$$

For convenience we take the reference construct to be the unidirectional 1.5×R_0 photonic wire. Having but a single arm and the largest dye spacing, this structure should have the smallest output and so the TEF is generally always an enhancement, *i.e.*, greater than one. It is also important to note that the concentrations (and illumination, etc.) used in the test and reference experiments need to be the same. If not, then (3a) needs to be corrected as

$$TEF = \frac{\Phi_A}{\Phi_{A,ref}} \frac{\rho^{(1)}_{ideal,ref}}{\rho^{(1)}_{ideal}} = \frac{\Phi_A}{\Phi_{A,ref}} \frac{A^{(1)}}{A^{(1)}_{ref}} \frac{\Psi_{ref}}{\Psi} \tag{3b}$$

Another relative measure of performance is the antenna effect (AE) that compares the output of the terminal acceptor of a construct when its primary donors are excited (*i.e.,* the Cy3s at 515 nm) with the output $\Phi_{A,ex}$ when the terminal acceptor alone is excited at its absorption maximum (at 650 nm for Cy5 and 700 nm for Cy5.5)[9]:

$$AE = \frac{\Phi_A}{\Phi_{A,ex}} \tag{4}$$

As a second measure of the antenna performance, we define an antenna gain (AG) where the normalization is to the output of the equivalent (i.e., with the same dye spacing) linear photonic wire $\Phi_{A,lin}$ since the latter represent point-to-point excitonic delivery without antenna action:

$$AG = \frac{\Phi_A}{\Phi_{A,lin}} \tag{5}$$

Obviously AG is the same as TEF with a particular definition for $\Phi_{A,ref}$. As in (3b), (4) and (5) must be corrected if the test and reference experiments have different concentrations, illumination levels, etc.

We define exciton transfer efficiency as the conditional probability that an exciton will transfer from a given excited donor to a given acceptor, with the process of most interest being the end-to-end transfer from a peripheral donor to a terminal acceptor. Like AG, this quantity is harder to estimate experimentally than TEF, AE or AG, but an empirical formula that has been used is

$$E_{\exp} = \frac{\left(\Phi_A - \Phi_A^0\right)/Q_A}{\Phi_D^0/Q_D} \tag{6}$$

where Q_A and Q_D are the QYs of the acceptor and donor, respectively[7,10,11]. In this expression, the denominator gives the number of excited donors (per second) while the numerator is the number of excited acceptors (per second) that did not become excited as a result of direct excitation. If Φ_D and Φ_A are the outputs of the peripheral donors and the terminal acceptor, respectively, then (6) will provide an approximation to the end-to-end efficiency with the proviso that direct excitations of intermediate dyes do not contribute significantly. It should be noted that the latter can be especially significant in all-organic systems that lack a QD's strong absorbance and its ability to be excited at "distant" wavelengths. On the other hand, if we are considering the constructs as light-harvesters, then any collection counts and the anywhere-to-end efficiency of (6) is then the appropriate measure.

An efficiency analogous to that of (6) is also readily estimated from the Förster analysis as the average conditional probability that an exciton generated somewhere within the structure (including on the central dye itself) will result in excitons reaching the focus. The anywhere-to-end efficiency so defined is computed as:

$$E_1 \equiv \left\langle \sum_{m=1}^{N} W_{focus}(\eta_m) \right\rangle \quad (7a)$$

where the angle-brackets indicate the ensemble average and the η_m are given by the values in Supplementary Tables 62-64. The Förster analysis also provides an excellent way of analyzing the complication of direct excitation of intermediate dyes since simulation allows us to have control over just where the initial excitation occurs. Thus the actual end-to-end efficiency can be calculated from

$$E_2 \equiv \left\langle W_{focus}(\eta_{peripheral} = 1) \right\rangle \quad (7b)$$

This quantity is computed using the Förster equations with all the parameters chosen to agree with experiment and then setting the direction excitation of one peripheral dye to one and all others to zero.

Supplementary Note 3: Estimating assembly efficiency for the DNA structures

Assembly yield estimates were obtained using both gel electrophoresis and fast protein liquid chromatography (FPLC). For the gels, the DNA structures were assembled as described above at a concentration of 0.5 µM. 10 µL of solution was mixed with 2 µL loading buffer and then separated in a 3% agarose (low electroendosmosis) gel in 1X Tris-borate-EDTA buffer (TBE, 89 mM Tris base, 89 mM Boric acid, 2 mM EDTA) at a current of ~100 mA while cooling the gels. BioMarker EXT Plus ladder (Bioventures) was also included on the gel to help estimate molecular weight and concentration. Each band in this ladder corresponds to a concentration of 100 ng DNA of DNA. For visualization, the gels were stained with 1X Gel Red dye (Biotium), 10 µL per 100 ml of agarose and visualized with a Gel Logic 2200 imaging system (CareStream Health).

Yields were obtained through image analysis such as that of the representative gel images in Supplementary Figure 9. Predicted sizes for the structures were estimated based on end-to-end length and total number of double-stranded basepairs present see Supplementary Tables 65-66. The intensity and area of each of the distinct bands were measured in each lane and tabulated using Image J (NIH). These were divided by the multiplied area and intensity of the nearest ladder band to account for differences in intensity between DNA sizes. Through analysis of the

observed banding, it is clear we have fully formed structures, partially formed structures, and unformed structures (unreacted oligos). The total area normalized intensity of all bands in a given structure were collated and the percentage of structure in a given category is reported in Supplementary Tables 39 and 53, which show estimates for the 2-dye structures and the 4-dye structures, respectively.

It is apparent from the gel migration that the larger 8-arm stars do not migrate specific to what is expected just from DNA size given their non-linear shape. The latter manifest what appear to be multiple structures which may include cross-linked assemblies. We qualify these also as partial structures, although, they may still retain the ability to engage in efficient FRET. This fact is exacerbated in the dendrimer structures and for those the FPLC method was more effective. We qualify our assembly efficiency values below as just estimates at this time and a more intensive study of their formation efficiency is underway for publication at a later date.

For the FPLC analysis, samples were formed at 0.5 µM at a total volume of 400 µL. The samples were analyzed on an AKTA purifier 10 (GE Life sciences). Samples are analyzed on a Superdex 200 10/300 GL at a flow rate of 0.5 ml/min. Three wavelengths can be collected simultaneously, so each sample with full dyes labeling was run twice. The first run collected wavelengths at 260 nm, 550 nm and 580 nm. The second run collected at 260 nm, 650 nm and 680 nm. The two separate sets of data were normalized to the 260 peak and consolidated into one plot. The FPLC results from the dendrimer (Supplementary Figure 25) show the separation of the formed solution divided again into formed, partial, and unformed peaks. Since the absorption of each fraction is hard to determine, the dye molar extinction coefficient was used to calibrate the intensity of each peak. The FPLC separates according to size. Since the dendrimer structures are close to the same size, the primary peak runs close to the same at 11, 10 and 9.5 mL volume fractions for 2:1, 3:1 and 4:1 dendrimers, respectively. Peaks with all dyes present but not in the fully formed peak as stated above are considered partial formations and peaks where only one or two dyes are present are considered unformed. The larger size presents a peak with a lower fraction volume collection at 10 mL, as compared to 11 ml with the 4-dye 2:1 dendrimer. Given the similar nature of the peaks between the 4-dye and the 5-dye systems, the 2:1 dendrimer was used as a standard for calculating the relative concentration of the two

primary peaks that are seen. Tabulated results for all analysis are indicated in Supplementary Tables 39 and 53.

Supplementary Note 4: spFRET analysis of 0.75×$\boldsymbol{R_0}$ $\mathbf{Cy3_n{\rightarrow}Cy5}$ structures

To better understand the ensemble FRET efficiencies of the linear, bifurcated, Holliday junction and 8-arm star $Cy3_n{\rightarrow}Cy5$ DNA structures, spFRET experiments were performed where each structure was labeled with Cy3 and Cy5, as described above for the $0.75{\times}R_0$ series. Figure 2D compares spFRET histograms for each of the structures. The FRET efficiency is calculated from the ratio $I_A/(I_A+\gamma I_D)$, where I_D and I_A are the photon burst signals from the donor and acceptor channels, respectively. The factor, γ, accounts for the photon detection efficiencies of the two channels, and fluorescence quantum yields of the donor and acceptor. For the experiments described here $\gamma \approx 1$. At initial incident laser powers <75 μW at the objective, we observed that the structures with more than one Cy3 donor showed increased relative intensity of the low FRET peak. This result may be attributed to an increased excitation generation rate as the number of Cy3 donors is increased within a structure, which in turn increases the probability for Cy5 photobleaching. To minimize the photobleaching, the laser power was reduced until the relative intensity of the low-FRET peak became insensitive to further power reduction. The high FRET peak observed for each construct (FRET efficiency ≈ 0.75 – 0.80) indicates that at least a fraction of the ensemble undergoes efficient FRET. In addition, we observe a slight increase in efficiency of the high FRET peak as the number of arms increases from one to eight. A distinguishing feature of the 8-arm histogram is the significantly and larger relative intensity of the low FRET peak, which persists even at the lowest laser power used for excitation. The low FRET peak is asymmetric with a distribution that tails towards intermediate FRET efficiencies. Taken together, these observations suggest that there are both high FRET and low FRET pathways within the more complex 8-arm structure, depending on which Cy3 is initially excited. The relatively large ring-like opening at the center of the 8-arm structure (~30 Å, see Supplementary Figure 51)[8] creates Cy3-Cy5 separations significantly longer than the intended distance of $0.75{\times}R_0$. Therefore, FRET from a Cy3 donor located across the ring from the Cy5 acceptor would be less efficient. This explanation is the simplest that is consistent with the lower overall ensemble FRET efficiency of the 8-arm star structure.

Supplementary Note 5: Förster model

For a detailed understanding of the various structures under consideration it is useful to develop a model of the internal exciton dynamics. This is of most value when one wishes to predict the potential performance of different designs, to apprehend why a particular experimental realization of a design falls short of this ideal, and to explore ways of improving performance. We base such an analysis on a set of coupled rate equations that describe the various energy transfer processes that can occur in a well-mixed solution of the photo-active constructs. Because heterogeneous mixtures of constructs are considered, it is convenient to normalize these governing equations by the total concentration and the variables then become equivalent to probabilities. In particular, if the probability of the ith dye on the kth type of construct within the ensemble being excited at time t is $P_{ik}(t)$, then the system will be governed by the coupled ordinary differential equations:

$$\frac{dP_{ik}}{dt} = \sum_{m=1}^{M} \Delta_{ik}^{m} \eta_m \delta(t) - \frac{P_{ik}}{\tau_{ik}} \left[1 + \sum_{j=1}^{N_k} b_{ij}^{k} \right] + \sum_{j=1}^{N_k} \frac{b_{ij}^{k} P_{jk}}{\tau_{jk}} \qquad i = 1,\ldots,N_k \quad k = 1,\ldots,S \qquad (8a)$$

where N_k is the number of dyes in the kth construct, $\tau_{ik} = \Sigma_m \Delta_{ik}^{m} \tau_m$ where t_m is the lifetime of a dye of type m ($m = 1,\ldots, M$), and Δ_{ik}^{m} is unity if dye i on construct k is of type m and is otherwise zero. The matrix element b_{ij}^{k} specifies the excitonic coupling between the dyes i and j on construct k and the terms containing the δ-functions model direct excitation with η_m being the probability that an absorbed photon creates an exciton on a dye of type m (see below). We shall assume that there are $S \geq 1$ different types of multi-dye FRET-active constructs in the heterogeneous ensemble that are labeled $k = 1, \ldots, S$, with $k = 1$ being the target construct. In addition, we allow for the possibility of there being "constructs" that are not FRET-active (i.e., because the dyes are isolated or are all of the same type) and thus can be treated as separate "free" dyes; these are labeled $k=S+1,\ldots,S+M$, and for them (8a) reduces to

$$\frac{dP_{ik}}{dt} = \eta_{k-S} \delta(t) - \frac{P_{ik}}{\tau_{ik}} \qquad i = 1,\ldots,N_k, \quad k = S+1,\ldots,S+M \qquad (8b)$$

where N_k is the number of free dyes of type k - S. For the multi-dye constructs that obey (8a), according to Förster theory the couplings between the dyes are *via* point dipole-dipole interactions for which

$$b_{ij}^{k} = \left(\frac{R_{0k}^{ij}}{r_{ij}^{k}} \right)^{6} \qquad \text{with} \qquad R_{0k}^{ij} = \sum_{m=1}^{M} \sum_{n=1}^{M} \Delta_{ik}^{m} \Delta_{jk}^{m} R_{0}^{mn} \tag{9}$$

where R_0^{mn} is the Förster distance given in (1), and r_{ij}^{k} is the distance between them.

Since the emission rate from dye i on construct k is given by $Q_{ik}P_{ik}/\tau_{ik}$ where $Q_{ik} = \Sigma_m \Delta_{ik}^m Q_m$ and the Q_m are the quantum yields of the dyes, the total number of photons emitted (per photon absorbed) from dye i on construct k will be $Q_{ik}W_{ik}$ where

$$W_{ik} \equiv \frac{1}{\tau_{ik}} \int_0^{\infty} P_{ik}(t)dt \tag{10}$$

The equations governing the W_{ik} are readily obtained by integrating (8a) and (8b) over time (and using the fact that no dyes are excited prior to the initial excitation or remain excited after infinite time). Equation (8b) yield $W_{ik}\eta_{k-S}$ (for $i=1,\ldots,N_k$, $k=S+1,\ldots,S+M$) directly, while (8a) reduces to S linear algebraic systems of dimension $N_k \times N_k$:

$$W_{ik}\left[1 + \sum_{j=1}^{N_k} b_{ij}^{k}\right] - \sum_{j=1}^{N_k} b_{ij}^{k} W_{jk} = \sum_{m=1}^{M} \Delta_{ik}^{m} \eta_m, \quad i = 1,\ldots,N_k \quad k = 1,\ldots,S \tag{11}$$

each of which can be solved separately for the remaining W_{ik}. Lastly, to connect with experiment we need to relate the quantities in (11) to the PL areas Φ_m of (15) that represent the total emitted energy into the detector per second by dyes of type m. Specifically, we can write

$$\Phi_m = \Psi Q_m \left[\sum_{k=1}^{S} \frac{\rho^{(k)}}{\rho_{ideal}^{(1)}} \sum_{i=1}^{N_k} \Delta_{ik}^{m} W_{ik} + \left(N_{m,ideal}^{(1)} - \sum_{k=1}^{S} \frac{\rho^{(k)} N_m^{(k)}}{\rho_{ideal}^{(1)}} \right) \eta_m \right] \qquad m = 1,\ldots,M \tag{12}$$

where $\rho^{(k)}$ is the molar concentration of construct k, $N_m^{(k)} \equiv \sum_{i=1}^{N_k} \Delta_{ik}^{m}$ is the number of dyes of type m in construct k, and $\Psi \equiv \rho_{ideal}^{(1)} A^{(1)} L \Omega$ is a scaled generation rate (where $A^{(1)}$ is the number of photons absorbed per second by a single target structure, L is the path length, and Ω is a

geometric factor expressing the fraction of emitted photons that make it to the detector). The first term in (12) is the contribution from the multi-dye FRET-active structures in the ensemble while the second term represents the “free” dye contribution. For an ideal assembly, $\rho^{(1)} = \rho^{(1)}_{ideal}$, $N^{(1)} = N^{(1)}_{m,ideal}$ and all other $\rho^{(k)}$ and $N^{(k)}_{m}$ are zero. Using single-dye control experiments and (15), one can measure $\Phi^0_m = \Psi\rho_0^{(m)} Q_m \eta_m / \rho^{(1)}_{ideal}$ where $\rho_0^{(m)}$ is the concentration of the particular dye in the control which is usually chosen to maintain the same stoichiometry, *i.e.*, $\rho_0^{(m)} = \rho^{(1)}_{ideal} N^{(1)}_m$. Then since $\Sigma_m N^{(1)}_m \eta_m = 1$ it follows that

$$\eta_m = \frac{\Phi^0_m}{\Psi Q_m N^{(1)}_m} \qquad \Psi = \sum_{m=1}^{M} \frac{\Phi^0_m}{Q_m} \tag{13}$$

The second equality in (13) provides a way of estimating the normalization, and since to the extent possible all is kept fixed in the experiments (concentrations, illumination, etc.), just one value of Υ can suffice for each value of *M* and dye spacing. However, one should still be aware of the possibility that differences from one experiment to another can cause Υ to change and thereby be a source of discrepancy between simulation and experiment.

Within Förster theory, the main geometrical factor affecting the photophysical response and efficiency is the relative positions of the fluorophores via (10). The orientation of the excited state fluorophore dipoles can also play a role through the dependence in (1) of R_0 on the dipole orientation factor κ^2. Given the flexible attachment of the dyes in our constructs we typically take κ^2 to have its ensemble-averaged random value of 2/3. However, given the fact that natural light-harvesting structures are known to control dipole orientation and that there is at least the possibility of such control in artificial systems (e.g., via double phosphoramidite linkages), we carried out a few simulations in which κ^2 took values other than 2/3.

A first point to be made about the effect of dipole orientation is that even if one had perfect control, the impact would be relatively small because of the 1/6 power dependence in (1). Depending on the relative orientation of the dyes κ^2 can vary from zero to 4, and as shown in Supplementary Figure 30 at best (*i.e.*, for parallel dipoles) R_0 will be only about 35% larger than when randomly oriented.

For our DNA-organized FRET networks, Supplementary Figure 30 tends to exaggerate the size of the possible enhancements that could be derived from control of dipole orientation. A primary reason for this is that even if the dipoles were oriented perfectly parallel along a DNA duplex, this would not be true of the dyes in different DNA arms and so all inter-arm FRET processes would be non-optimal. To investigate things quantitatively we used simulation to study the impact of having oriented dipoles on the anywhere-to-end efficiencies of some of our FRET networks. In particular, in Figure 6E we compare simulations of an 8-arm, 4 dye star network (Figure 1B) in which the dipoles are random ($\kappa^2 = 2/3$) with a case when the dipoles are all parallel *when the structure is flat*. The main plot shows results with ideal formation, whereas the inset assumes "actual" formation as inferred by the curve-fittings discussed in this paper. In both cases, the effect of dipole orientation control on overall FRET efficiency is not large. Moreover, the largest impact is seen when the dye spacing is near $1.0{\times}R_0$ since this is where the Förster coupling is most sensitive to all parameters, and this represents another reason why dipole orientation is a secondary consideration in FRET network design: for maximum efficiency the main factor is dye spacing, and when this is reduced below $1.0{\times}R_0$ the consequences of dipole orientation control are even further reduced.

The results of two other calculations regarding dipole orientation control appear in manuscript Figure 6F. In the main plot, the anywhere-to-end FRET efficiency of the fully formed 8-arm, 4-dye star with dye spacing of $1.0R_0$ is studied as a function of misalignment of the dipoles. In this simulation, the angle of the dipoles with respect to the DNA axes is varied, while the azimuthal angle is random so that as the dipoles incline away from the DNA axis they go out of parallel alignment and the efficiency falls. From this plot we see that one needs to keep the misalignment less than 20° in order to preserve the efficiency gains. The second calculation in Fig. 6F examined more closely the random dipole case by studying two limits investigated in[12]. The first limit, referred to as dynamic averaging, follows the approach of elsewhere in this paper in which $\kappa^2 = 2/3$ as is appropriate if the dipole re-orientation time is *fast* compared to the lifetime. The other limit obtains when the dipole re-orientation time is *slow* compared to the lifetime, so that in a given construct the dipoles will be random but fixed in orientation during the measurement. The averaging that occurs is then over the ensemble and may be referred to as static averaging. The inset of Figure 6F compares these two limits with regards to the FRET

efficiency of linear photonic wires of varying length and with dye spacing of $0.75R_0$. Static averaging is seen to yield significantly lower efficiency; the relevance of this result to our studies (where we mostly assume dynamic averaging) is unknown.

Supplementary Discussion

Data Analysis/Simulations

In order to use the foregoing equations to analyze spectral data, we need values for the various parameters contained in these equations. Some of these parameters such as the quantum yields Q_i and the R_0 values (from (1)) are reasonably well established, while others such as the inter-dye distances r_{ij}^k are less so. In the case of a simple dye-pair it is well known that one can invert the procedure and use the FRET itself to deduce the separation distance [13], but this spectroscopic ruler approach is unworkable for the complex structures considered in this paper. Most intractable of all is the fact that our situations are usually heterogeneous due to the flexibility of the structures, possible self-quenching or photo-bleaching of dyes, inefficient assembly, etc. Given these realities, our goal has to be more modest, and we look merely for sufficient-but-not-necessary interpretations of the data through which we pursue not absolute agreement with data (which would perforce require uninformative curve fitting), but rather to address semi-quantitative questions such as are the systems describable by Förster theory, are the designs performing more or less as expected or is their evidence of problems, and most importantly what lessons can be learned about light-harvester design.

As already discussed, rough estimates of the nearest-neighbor dye spacing's can come from the DNA designs and the distances between the dye attachment points (see Eq. (15) and Supplementary Tables 62-64). However, for better accuracy one must also account for the small distances between the attachment points and the actual dyes (i.e., the locations of the point-dipoles of the Förster approximation) as determined by the dye linkage chemistries and the dye molecules themselves. And while there are sophisticated ways of gauging these dimensions, given the number and complexity of the situations considered in this paper, we instead employ a much less demanding approach. (The most accurate method at present for determining dye positions is one that combines molecular-dynamics (MD) simulations with single-pair FRET measurements[14]. MD simulations can also be used alone, and though less accurate, can still be quite informative[15]. Finally, for protein-based natural light-harvesters, X-ray diffraction has been invaluable.) In particular, we simply assert "reasonable" values for these linker/dye distances, and look for validation in the results obtained when these distances are held fixed across many other structures with the same dyes and linkage chemistries. For most of the dyes

considered, these attachments have significant flexibility and this aspect is represented both by taking the dipole orientation factor κ^2 to have its random value as already noted and by letting the linker orientation also be random over a defined range. For non-nearest-neighbor dyes, things are even more complicated because of the possibility that the interconnecting DNA scaffold can be flexible so that (15) no longer applies e.g., if there is an intervening Holliday junction. In our simulations we treat such flexible junctions by assuming they can take random angles over a specified range, and then capture the aggregate effect through ensemble averaging over many configurations. The most complex situations of this type are those of the 4- and 8-arm stars that have central openings that force the central dye to be asymmetrically located as depicted in Supplementary Figure 31. These openings have diameters of approximately 15Å and 30Å, respectively, which we represent crudely as DNA rings (see Supplementary Figure 32A,B) to which are attached the central dye and the DNA arms. The structural flexibility of the DNA (including of the ring) is then captured entirely by the orientation of the arms with respect to the ring with the angles of attachment again treated as random variables uniformly distributed over a defined range. As an example, a depiction of one configuration of the 4-dye, 8-arm star structure as used in simulation is shown in Supplementary Figure 33. And the importance of including such non-planar structures in the simulations is illustrated in Supplementary 34 where we compare the computed spectrum of the 4-dye, 8-arm star obtained by averaging over the full non-planar ensemble with that found assuming planarity.

Our general approach to the simulations is to proceed from the simplest cases involving two dyes and work up to more complex structures, at each stage comparing the ensemble-averaged spectra derived from modeling with experimental data. Simulating the integrated sub-spectra Φ_m would be equivalent but we prefer fitting the spectra for it's more direct and intuitive connection with experiment. Three levels of simulation are considered and are ideal simulations, parameter adjustments, and low-yield simulations.

For ideal simulations, perfect yield of the target structure is assumed and ideal parameter values including those given in Table 1 and Supplementary Tables 60-64 plus a set of dye/linker distances and a range of junction angles that, as noted above, are kept fixed to reflect the fact that our structures mostly involve the same dyes and linkage chemistries. The only specific fitting done in these simulations is of the multiplicative generation factor Ψ that we adjust from the

values given in Supplementary Table 61, almost always by less than 20%, under the presumption that this accounts for differences between the test and control experiments in sample concentrations, illumination, etc.

For parameter adjustments, if the discrepancies between the ideal simulations and the experimental spectra are relatively small, then we look to account for the differences with plausible adjustments of various parameters such as the dye/linker distances or the R_0 values (that could be affected by local dielectric perturbation[7]). These simulations continue to assume that the target structure is assembling properly, however, small reductions in yield can be considered as a way of representing, for example, the slight fall-off in assembly yield that would result if for example the initial concentrations were not precisely stoichiometric. Given the small contributions of the effects considered, identifying which one(s) is actually responsible is likely impossible within the present work because of its many uncertainties.

For low-yield simulations, should the parameter adjustments be found insufficient to produce agreement with experiment, we conclude that the assumption of perfect yield is flawed. To account for this we allow certain dyes to be inactive or quenched, or to simply be missing from the structure as a result of an incomplete assembly. As a result, the simulated ensemble will now be composed of the actual target structure plus various partial structures and leftover free dyes, all with specified concentrations, and with the aggregate photoemission described by (12). While clearly justifiable in general, the fact that this approach introduces a large number of new parameters (i.e., the concentrations) means it can fit almost any data and so can easily turn into an exercise in curve-fitting with little physical meaning. And unfortunately the electrophoresis/chromatography experiments do not provide a very substantial cross-check. For example, in a star construct that lacks a central dye the individual arms largely decoupled, and this implies that if the arms were physically separated the PL spectra would remain essentially unchanged whereas the gels would look completely different. Conversely, self-quenching of dyes in a structure would cause its spectrum to look entirely different while leaving the electrophoretic mobility unaffected. Given these realities, the quality of the individual fits to data are not especially meaningful, and we must instead judge the physical fidelity of our low-yield models from the reasonableness and simplicity of their basic assumptions and from the consistency and plausibility of the overall understanding.

Because our study of these DNA-organized dye constructs is motivated by their potential for funneling excitonic energy, the cases with the *smallest* dye spacing's are of most interest since their transfer efficiencies should be highest. From the standpoint of understanding, these cases are also most likely to be of value since the photophysical effects of interest should be largest. At the same time, however, one needs to be aware that if the dye spacing's become too small then new complications can arise. This is most likely the case for the Cy5 and Cy5.5 dyes with nominal $0.5R_0$ spacing where the linker attachment points are just ~20Å apart. With the dyes roughly ≤10Å in size and with flexibility in the linkers (especially of the Cy5.5), non-Förster interactions are likely, if not certain, and this can include a self-quenching effect known to occur when multiple Cy5 dyes are in close proximity [16]. Finally, at the other extreme, when the dye spacing's are large and the exciton transfer weak, one expects the spectra to be dominated by the direct PL of the dyes and to be relatively insensitive to errors in the inter-dye spacing's, and so the predictions of ideal simulation should be most accurate.

Simulation Results, Cy3/Cy5 2-dye constructs

In this section we model 1-, 2-, 4-, and 8-arm star structures in which Cy3 dyes are positioned at the distal ends of each of the DNA arms and a single Cy5 dye is located at or near the center as depicted in Supplementary Figure 31. For this dye-pair, R_0 is around 54Å (see Table 1), and the structures were designed to have nominal nearest-neighbor dye spacing's of $0.75R_0$, $0.85R_0$, $1.0R_0$, $1.25R_0$, and $1.5R_0$ for a total of 20 distinct assemblies. The nominal dye spacing's serve as a convenient reference, but as described earlier the actual values used in the simulations are estimated from the attachment point spacing's with small corrections to account for the linker/dye distances. The 1- and 2-arm designs have no intervening bendable junctions in their DNA scaffolds and so their attachment point distances are well estimated by the values computed with (15) and given in Supplementary Table 60A. For the 4-arm and 8-arm designs our treatment is based on the idealized geometries of Supplementary Figures 32A,B in which a wide range of random arm angles is allowed and with the results found to be relatively insensitive to the exact choice. Lastly, we find that fixing the linker/dye distances to be 2Å for the Cy5 dye and 8Å for the Cy3 dye across all simulations works well and is also consistent with the known chemistry as discussed earlier.

As a first set of ideal simulations obtained using the parameter set just described, in Supplementary Figure 35a we compare simulation and experiment for 1-arm linear structures having all five dye-spacing's. As is evident from the figure, excellent agreement is obtained when the nominal dye spacing's are 1.5R_0, 1.25R_0, and 1.0R_0, however, discrepancies appear when these spacing's are reduced to 0.85R_0 and 0.75R_0. Given the small size of the discrepancies, it is easy to adjust various simulation parameters and get improved fits, though as noted previously identifying which adjustment(s) is physical is problematic. For instance, one explanation could be that the inter-dye distance has somehow increased, with the needed additions being just 1Å and 5Å, respectively. Another possibility is that the R_0 value has decreased by similar amounts. This could occur as a result of non-randomness in the dipole orientation factor κ^2 of these phosphoramidite-linked dyes, *e.g.*, a 5Å reduction in R_0 is produced if increases from 0.67 to 1.2. Finally, it could be that there has been a slight drop in the assembly yield of these shorter strand samples that is not detectable by gel electrophoresis (Supplementary Tables 39 and 53) and that could result from slightly non-stoichiometric starting materials; under this assumption, the good fits shown in Supplementary Figure 35b are obtained when the respective yields are taken to be 93% and 82%. No matter which explanation is used, the simulations of Supplementary Figure 35a,b suggest that these constructs are well described by Förster theory.

Moving on to the multi-arm stars, we choose all parameters to be as in the 1-arm case plus for the 4-arm and 8-arm stars we employ the idealizations of Supplementary Figure 35a,b. The simulated spectra are plotted in Supplementary Figure 35c-e, and again we find good agreement with experiment for the larger dye-spacing's but growing discrepancies as these spacing's are reduced. That the disagreements between simulation and experiment are largest for the 4-arm and 8-arm stars and that electrophoresis also shows these cases to have issues with formation efficiency points to this as the cause. A fall-off in dye performance (e.g., by self-quenching) is also a possible interpretation, but this seems less likely as the inter-dye distances are relatively large (>35-40Å). Focusing then on assembly yield, the fact that the structures have just two dye types with a single nominal Cy3-Cy5 dye-spacing in any sample simplifies the interpretation in that, to first order, the ensemble can be treated as if it consisted just of fully formed structures and free Cy3 dye (since the individual arms function essentially independently and there is little

direct excitation of any free Cy5 dyes). Excellent fits to the spectra can be obtained in this way (not shown except in the 1-arm case in Supplementary Figure 35b), and the required assembly yields of the putative fully formed structures are plotted in Supplementary Figure 36 (solid lines) as a function of the dye spacing as measured in DNA base pairs. Also shown in the figure are the yields as estimated by electrophoresis, with both the full (dash with symbols) and full+partial (solid with symbols) formation percentages plotted. The fact that the simulated yields are generally in much better agreement with the full+partial data can be interpreted to mean that the partial structures seen in electrophoresis contribute substantial FRET and so must contain both Cy3 and Cy5 dyes in fragmented assemblies. That the DNA strands containing the Cy5 dyes are much longer than those with the Cy3 dyes makes this conclusion unsurprising. More generally, the rough consistency with the electrophoresis suggests that the low-yield interpretation and its associated fitting parameter are physically meaningful, and especially for the 8-arm assembly where plausible adjustments of other parameters are manifestly insufficient to account for the differences between the ideal and experimental spectra. This is less clear for the other assemblies (and especially the 1-arm linear structure as discussed above) since their ideal spectra are much closer to the data and the interpretation of them as also arising from formation inefficiency (Supplementary Figures 35b and 36), though consistent with the 8-arm treatment, could be incorrect.

To the extent that the above modeling provides an accurate representation of the photophysics of the two-dye star structures, we can now use that understanding to estimate efficiency and gain parameters. The anywhere-to-end efficiencies (E_1) are plotted in Supplementary Figures 37 (solid lines), with the end-to-end efficiencies (E_2) not shown because they are nearly identical due to there being little direct excitation of the Cy5 dye. Also shown in the figure (dashed lines) are the ideal E_1 values that would be obtained if the assembly and dye performance were perfect. There are several observations to be made from this plot. First, that the ideal efficiencies do not depend on the number of arms to first order indicates that the exciton transport occurs essentially independently in each arm. This suggests that homoFRET processes are unimportant, and apart from one exception discussed below, this is confirmed simply by turning off the homoFRET channels in the simulations. A second point to be made concerns the drop in efficiency observed in the 8-arm stars (and to a lesser extent in the 4-arm stars) as the

spacing between the dyes is reduced. Clearly this is due mostly to the decline in assembly yield discussed in connection with Supplementary Figure 36, however, that the close-spaced 4- and 8-arm stars are less efficient even under ideal conditions shows that there is more going on. Specifically there is additional inefficiency that is geometric in origin coming from the large central openings in these structures (see Supplementary Figure 32A,B). These openings cause the central Cy5 dye to be placed off-center, thereby increasing the average inter-dye spacing and lowering efficiency especially for smaller nominal spacing's (as seen in Supplementary Figure 37).

In Supplementary Figure 38 we plot the antenna gains (AG) as computed for the two-dye stars using (5) as a function of the dye spacing and corrected as discussed in connection with (3b). Also shown in the figure are ideal values of AG computed assuming perfect yield and fully active dyes. Since the arms operate essentially independently, it is not surprising that the ideal AG is more or less equal to the number of arms. The drop-off in the actual AG seen in the 4-arm and 8-arm stars for small dye-spacing's is of course mostly due to the formation inefficiency of these structures seen in Supplementary Figure 39. And the drop in all curves at larger dye spacing's (and especially for $1.5R_0$) is due to the Cy5 emission becoming increasingly dominated by direct excitation in that regime.

Finally, as noted earlier, for most purposes the essential information regarding assembly yield is the percentage of Cy3 dyes that are associated with Cy5 dyes no matter whether they are in full or in partial structures. But this would be the case only if all the Cy3 dyes were equivalent and this is not true because of the realities of DNA design and especially for the 4-arm and 8-arm constructs with their asymmetrically placed Cy5 dyes (Supplementary Figure 32A,B). The consequences are best seen in single-pair FRET measurements, which allow one to distinguish different energy-transfer pathways. The effect can also be seen in simulation; for example, in Supplementary Figure 39 we plot the simulated efficiency of each pathway in an 8-arm star with dye spacing of $0.75R_0$ and perfect formation efficiency assumed. In this plot, Cy3 positions 1 and 9 refer to the arm that joins the ring at the point where the Cy5 is located so that it has the shortest Cy3-to-Cy5 distance and highest efficiency. The opposite extreme is path 5 that involves the arm that attaches at the opposite side of the ring and so has the longest Cy3-to-Cy5 distance and the lowest efficiency. Most interesting is the fact that these differences (in both

geometries) are not as large as they would be if not for homoFRET. In particular, homoFRET produces an enhancement when the Cy3 is furthest away, and shows that the homoFRET pathway through multiple Cy3 dyes is making a substantial contribution.

Simulation Results, Photonic wire constructs

We next turn to the multi-dye star constructs considering 12 different DNA templates having 1-, 2-, 4-, and 8-arms and three sets of potential dye spacing's with the nominal values of $0.5R_0$, $1.0R_0$, and $1.5R_0$. On to each DNA scaffold, Cy3, Cy3.5, Cy5, and Cy5.5 dyes are added progressively inward from the periphery, and ending with star structures with differing numbers of arms and dye spacing and each having a Cy5.5 dye at the focus. For these structures the dye attachment distances are as computed using (2) and listed in Supplementary Table 60b. The random arm angles of the 4-arm and 8-arm structures are as in the previous sub-section, and again we take the linker/dye distances for the Cy3 and Cy5 dyes to be 8Å and 2Å, respectively. In addition, we assume the more loosely held Cy3.5 and Cy5.5 dyes have linker/dye distances of 10Å in all structures.

We begin with comparisons between the experimental and simulated results for the 12 constructs with nominal dye spacing of $1.5R_0$, in 1-, 2-, 4-, or 8-arm configuration, and labeled with either two (Cy3 and Cy3.5), three (Cy3, Cy3.5 and Cy5), or four (Cy3, Cy3.5, Cy5 and Cy5.5) dyes. We expect that the large dye spacing's will make the FRET in these structures rather weak so that "ideal" simulations should work well with the results relatively insensitive to formation assembly or dye performance issues. These expectations are largely met by the simulated spectra plotted together with the experimental results in Supplementary Figure 40a-c, where the agreement is seen to be generally excellent. The only significant discrepancy is for the 8-arm star with four dyes in Supplementary Figure 40c (especially around 670nm), and this error is puzzling since it seems unexplainable by either assembly yield (since the prediction *underestimates* the level of FRET) or inter-dye distance errors (since the reductions of roughly 10Å in *each* dye spacing needed to explain the observed FRET seem rather large).

Next we compare experiment and simulation for the 12 star constructs with nominal dye spacing of $1.0R_0$, as before having 1, 2, 4, or 8 arms and labeled with two (Cy3 and Cy3.5), three (Cy3, Cy3.5 and Cy5), or four (Cy3, Cy3.5, Cy5 and Cy5.5) dyes. We start with ideal

simulations in which all parameters including the linker/dye distances remain the same. The results are shown in Supplementary Figure 41a-c, where again the simulations are compared with experiment. The results are again quite good, and especially in the case with just Cy3 and Cy3.5 (Supplementary Figure 41a) where only minor discrepancies are seen, and for which even better agreement can easily be achieved with small parameter adjustments. An illustration appears in Supplementary Figure 42a where the yield has again been used as the fitting parameter (as in Supplementary Figure 35b) and only small adjustments are needed to obtain excellent fits. As additional dyes are included, the "ideal" emission is no longer as accurate, and especially when the Cy5.5 is included (Supplementary Figure 41c). In these cases the parameter adjustments needed start to become larger than seems consistent with the perfect yield assumption, and this conclusion us supported by the electrophoresis data. We therefore proceed with "low-yield" simulations, and using yield as the fitting parameter, we obtain the results shown in Supplementary Figure 42b-c that are now in excellent agreement with experiment. To understand these results it needs to be explained that the yield as expressed in the percentage labels in these figures refers to the fractions of the ensemble made up of the target structure (the rightmost percentage) plus one or two other partial structures (the other percentages). In particular, we assert that the only partial structures present in the ensemble are ones with each dye in *full* complement but with just two (Cy3 and Cy3.5, first percentage) or three (Cy3, Cy3.5 and Cy5, second percentage) dye types present, and with all the remaining dyes that are not incorporated into these structures (and therefore not participating in FRET) being treated as free dyes. This representation of the partial structures is meant to capture the composite contribution of a wide variety of potential non-fully-formed structures, e.g., by possibly clustering multiple separated arms into complete stars. But it should be noted that here the treatment is less solid because there is just one Cy5.5 in the 4-dye structures and so the approach misses the fact that separated arms containing a Cy5.5 act differently than the full target structure. Now, as before, the assumption of reduced yield brings extra fitting parameters (i.e., the yield percentages of each structure) that make good agreement with experimental spectra like those in Supplementary Figure 42 neither surprising nor especially meaningful. It is potentially of more value in assessing physical content to look at the yields obtained from the spectral fittings (Supplementary Figure 42a-c). Such a plot appears in Supplementary Figure 42d where we

show the computed yields for the target structure (dashed lines without symbols) and for the sum of all FRET-contributing structures (solid lines without symbols). Also shown in the plot are the full (orange dashed line with symbols) and full+partial (orange solid line with symbols) formation percentages as obtained from electrophoresis. Several aspects of this plot deserve comment. First, that the simulated total yields are always high is another indication that the Cy3 and Cy3.5 dyes always assemble with high fidelity and that the non-ideal behavior is entirely associated with the Cy5 and Cy5.5 dyes. Second, that none of the simulations track the gel-measured full-formation curve suggests that there is considerable FRET occurring in the partially formed structures seen in electrophoresis. Third, that all of the simulated yields are relatively independent of the number of arms is consistent with a formation inefficiency (or inactive/photobleached dyes) explanation in these relatively uncrowded structures. This also argues against possible self-quenching of the dyes as an explanation for lowered yields; that the dye spacing's in these structures are fairly large (>45Å) also argues against this mechanism. Finally, that the 4-dye yield is low (~50%) suggests that it is a real but not understood consequence of formation inefficiency (or inactive/photobleached Cy5.5, or perhaps of Cy5 in the presence of Cy5.5), and is not an artifact of our assuming that there are no partial structures containing Cy5.5 dyes. If partial structures containing Cy5.5 played much of a role this would raise the number of the other partial structures and free dyes and lower the output of the Cy5.5 dyes. As a result, the 4-dye yield needed in our simulations to fit the Cy5.5 emission in our experiments would have to go up as the number of arms increased. That the yield is instead flat suggests this is not happening. Of course, it could be that the actual yield of the full structures is lower and that this is offset by the wrong treatment! In any event, while less certain than the previous cases considered, the experiments on 1.0R_0 multi-dye stars do seem understandable in terms of Förster theory.

The last set of multi-dye star constructs are the 12 structures that have the minimum nominal dye spacing of 0.5R_0, and are again in either the 1-, 2-, 4-, or 8-arm configuration and labeled with two (Cy3 and Cy3.5), three (Cy3, Cy3.5 and Cy5), or four (Cy3, Cy3.5, Cy5 and Cy5.5) dyes. All parameters are chosen as earlier and the ideal simulation results are plotted together with the experimental spectra for the two, three, and four dye cases in Supplementary Figure 43a-c, respectively.

In general, the results are similar to those with $1.0R_0$ spacing in that the agreement in the case of the two-dye constructs (Supplementary Figure 43a) is reasonable good, but in the other cases (Supplementary Figure 43b,c) the results are far worse. Focusing first on the two-dye constructs, we observe that the best results are curiously obtained for the 4-arm and 8-arm structures, but note that this is probably not meaningful given the small differences involved and the fact that minor changes in the linker/dye lengths or other parameters would lead to other conclusions. Indeed, looking to account for the discrepancies in Supplementary Figure 43a with small parameter adjustments, in Supplementary Figure 44a we see that plausibly small changes in the yield (as might occur from errors in stoichiometry) result in excellent fits to all of the data. Clearly, the much larger differences seen in the three (Supplementary Figure 43b) and four (Supplementary Figure 43c) dye cases cannot be fit in a similar way and one must consider low-yield as an explanation, for which there is also electrophoretic evidence. The curve-fits obtained using low-yield simulation are shown in Supplementary Figure 44b-c and again it is no surprise that excellent agreement is obtained. The partial structures assumed to exist in the ensemble are the same as those of Supplementary Figure 42b,c with the percentages listed having the same meanings as previously. And again these partial structures are meant to represent the aggregate response of the wide variety of partial structures that could exist in the experiment. To judge the sensibleness of the results, in Supplementary Figure 44d we again plot the spectra-derived yields using the same format as in Supplementary Figure 42d. As before, the fact that the total yield of full+partial structures is high in all cases indicates that the assembly of the Cy3 and Cy3.5 dyes occurs quite efficiently, that the partial structures measured electrophoretically contribute to the Cy3-Cy3.5 FRET, and that the performance issues are entirely associated with the Cy5 and Cy5.5 dyes. In contrast to the $1.0R_0$ case, the yield of fully assembled structures with three and four dyes (dashed curves in Supplementary Figure 44d) do vary with the number of arms and qualitatively track the gel results. The correspondence with the gel results suggests that this is due to a crowding effect in the 4-arm and 8-arm structures that impairs hybridization rather than to a self-quenching that has been reported to occur between Cy5s when these dyes are in close proximity. To first order, to have a functioning four-dye construct requires assembly of the three-dye construct, and the fact that the yield of the latter is poor shows that a weak Cy5 assembly is probably mostly responsible for the four-dye result as well. This is also supported

by the fact that the DNA to which the Cy5 is attached is considerably shorter than that of the Cy5.5. Why the spectra-derived (but not gel-derived) yields are lowered also when the structure has one or two arms is less clear. It could be that with these short strands, the gel is not distinguishing a full one-arm structure from one without the Cy5 hybridized. As with the $1.0R_0$ situation, the foregoing suggests that (with appropriate caveats) the $0.5R_0$ spectra are also consistent with Förster theory and likely involve a greatly reduced formation efficiency of the Cy5 dye.

Assuming the above has indeed provided a plausible understanding of the photophysics of the multi-dye stars, we can now estimate their efficiency and gain parameters. In doing so, we focus on the complete four-dye structures that have a Cy5.5 at the center to which excitonic energy is being delivered. The results for the anywhere-to-end efficiency E_1 as computed from (7a) are plotted in Supplementary Figure 45a with both actual and ideal results shown. The ideal results clearly display the expected behavior of the FRET efficiency rising strongly as the dye spacing is reduced. In addition, the fact that the ideal curves are relatively flat is further evidence of the independence of the arms in terms of energy transfer. That there is some rise in the ideal curves for the $1.0R_0$ and $0.5R_0$ cases shows, however, that there is a portion of the energy transfer that occurs on parallel paths (see below). The actual efficiencies are of course greatly reduced in the $1.0R_0$ and $0.5R_0$ cases due to yield issues. As we have seen these problems are especially severe in the $0.5R_0$ case and with multiple arms; in those cases, the efficiency is seen to fall even below that with the $1.0R_0$ spacing. The ideal end-to-efficiencies E_2 are not shown in the plot but are roughly 10% less that E_1 for the narrower dye spacing's. Interestingly, for the $1.5R_0$ constructs, E_1 is in the range of 1.3-2.0% (as seen in Supplementary Figure 45a), whereas E_2 is 0.1-0.3%, thus indicating that most of their E_1 arises from direct excitation of the terminal dye.

To explore more carefully the effect of parallel paths, in Supplementary Figure 45b we plot the ideal E_2 for the dye spacing's of $0.5R_0$ and $1.0R_0$ and compare simulations in which all FRET processes are permitted with ones in which the FRET is restricted to occurring only on the direct paths connected by DNA. As expected, there is no difference between these quantities when the constructs have one or two arms. However, in the case of 4- and 8-arm designs, a growing component along parallel paths (*i.e.*, the difference between the curves) is seen.

The antenna gain AG for the complete four-dye star structures, again calculated from (5) with the correction made as in (3b), is plotted in Supplementary Figure 46 with both ideal and actual curves shown. The ideal curve is close to the unity slope that would be expected if all arms operated independently; that the slope is slightly higher again reflects the contribution of parallel paths. Of course the actual AG is much lower, and again this is due to the low yield of these structures.

Simulation Results, Dendrimers

The third category of light-harvesting structures studied was DNA dendrimer assemblies, with only the case of a nominal dye spacing of $0.5R_0$ being investigated. Designs with dendrimer branching ratios of 2:1, 3:1, and 4:1 were examined, and as with the multi-dye stars, on each template the Cy3, Cy3.5, Cy5, and Cy5.5 dyes were added progressively inward from the periphery ending with three structures (with the three different branching ratios) having a Cy5.5 dye at the focus. In addition, two other 2:1 dendrimers were studied that had five dyes (instead of four), with one having the set [A488, Cy3, Cy3.5, Cy5, and Cy5.5] while the other had [A488, Cy3, Cy3.5, A647, and Cy5.5]. The ideal simulations were performed exactly as before with the parameters as in Table 1 and Supplementary Tables 60-64 and the same values for the linker/dye distances. The variation among arm angles is again treated as a random variable over a specific range with the results not especially sensitive to the choice of this range.

Starting with the dendrimers having potentially four dyes, we plot the ideal simulations along with the experimental data in Supplementary Figure 47a-c. The correspondence is even worse than before, with even the Cy3-Cy3.5 structures not seeming to show very close agreement. Nevertheless, attempting to fit the Cy3-Cy3.5 dendrimer data using small parameter adjustments, we do find that small reductions in the yield do allow the experimental spectra to be fit quite nicely as is seen in Supplementary Figure 48a. So again it seems that the Cy3 and Cy3.5 dyes are assembling nearly perfectly in these structures. The other cases clearly require abandonment of the assumption of perfect assembly (just as was the case in other similar designs we have considered). How to do the low-yield simulations is less clear than before because the dendrimers lack the radial geometry of the stars and as a result there seems no obvious way of

representing the partial structures with just a few meaningful fitting parameters. Absent a better procedure we use the same approach as earlier of having the partial structures be full apart from missing entire layers of the inner dyes (Cy5 and/or Cy5.5). The results are plotted in Supplementary Figure 47b,c with the meaning of the percentage labels as before. Given the additional fitting parameters, the fits are of course excellent, but any real meaning must be looked for in the yield numbers. A plot of these yields appears in Supplementary Figure 47d, along with the experimental formation efficiencies derived from electrophoresis. That the yield on the full+partial structures is high in both simulation and experiment can again be taken as evidence of the assembly efficiency of the Cy3 and Cy3.5 dyes in all constructs including the partial structures observed in electrophoresis. The three- and four-dye yields are much lower, and without a strong correlation with the branching ratio, again suggest that the main effect is formation inefficiency with self-quenching likely playing no role. Although the nominal spacing in these structures and the third group of multi-dye stars was $0.5R_0$, the Cy5-Cy5.5 attachment distance in the dendrimer is ~35Å whereas in the stars it was ~20Å. This is another argument against a self-quenching mechanism. As with the multi-dye stars, to first order achieving a four-dye dendrimer requires the ability to assemble a three-dye dendrimer. That the yield of latter is greatly reduced and is similar in both magnitude and trend to the four-dye curve suggests that the Cy5 dye is again the source of the problem.

The ideal simulations for the 5-dye dendrimers appear in Supplementary Figure 49a,b along with the corresponding experimental data. Generally, the agreement is again not so good except for the structures with just a few dye types present. Proceeding to low-yield simulations using the approach outlined in the previous paragraph, we obtain the curve-fitting results shown in Supplementary Figure 49c,d. The most interesting observation from these plots is that both the two-dye and the three-dye curves are well fit without too large a parameter adjustment. This suggests that the formation efficiencies of the A488, Cy3, and Cy3.5 are all reasonably good. However, the hope that the A647 dye (Supplementary Figure 49d) might behave better than the Cy5 (Supplementary Figure 49c) is not borne out; in both cases the yield drops precipitously at that stage.

We next turn to estimating the end-to-end efficiency (E_2) for the four-dye dendrimers with branching ratios of 2:1, 3:1, and 4:1. The results are shown in Supplementary Figure 50a,

together with the corresponding result for the dendrimer with a 1:1 branching ratio, which is simply the one-arm star discussed earlier (Supplementary Figures 41c and 42c); it should be noted that this is not a perfect analog in that its dye spacing's are somewhat different from the other dendrimer structures. The solid blue curve in Supplementary Figure 50a is for the actual structures as modeled above, while the long-dash red curve is for the ideal structure with perfect yield. Obviously the strong decline in the actual efficiency is due to the poor yield seen experimentally. In the ideal case, the efficiency rises with increasing branching ratio by about 30%, although the 3:1 and 4:1 cases are not especially different. The reason for both the rise and the saturation is the parallel paths in the structure. To investigate this further we performed some additional simulations in which the FRET was restricted to being only between dyes on a given branch (Supplementary Figure 50b, right side) or further limited only to nearest neighbors on a given branch (Supplementary Figure 50b, left side). These simulations show that the efficiency improvement with branching ratio comes from two sources. When only nearest-neighbor dye couplings are included, then one finds that there is no efficiency enhancement resulting from branching ratio at all as seen in Supplementary Figure 50a (dashed green curve); in fact, a slight drop in E_2 is observed due to variations in the DNA strand lengths. But when couplings are allowed just between all dyes on the same branch (but not separate branches) as depicted in Supplementary Figure 50b (right side), then one finds a large portion of the enhancement of the full ideal curve (that also includes inter-branch contributions) is realized (purple small-dash curve). Thus both intra-branch and inter-branch parallel paths contribute to the efficiency enhancement and these make the dendrimers inherently more efficient than the star constructs.

Lastly in Supplementary Figure 50c we plot the antenna gain of the actual and ideal 4-dye dendrimers, again including the linear 1-arm structure as the 1:1 branching ratio point. As expected, there is much potential for dramatic (exponential) increases in collection capacity with a dendrimer design, with the 4:1 dendrimer ideally producing a gain of nearly 400. Of course, as implemented by us the realized AG is far worse due to the yield issues, so much so that AG for the 4:1 dendrimer actually falls.

Supplementary Methods

Design of DNA sequences

The DNA in these experiments were designed synthetic sequences that were purchased from Integrated DNA Technologies (Coralville, IA) with the exception of the Cy3.5, and internal labeled Cy5.5-functionalized strands which were purchased from Operon Biotechnologies, Inc. (Huntsville, Alabama); see Supplementary Tables 1-37 for specific sequences and predicted melting temperatures (T_m). Sequences used for the linear, bifurcated, and Holliday junction $[Cy3\rightarrow Cy3.5\rightarrow Cy5]_n\rightarrow Cy5.5$ photonic wires were adapted from ref[7]. The sequences for the center of the 8-arm stars and for all of the $Cy3_n\rightarrow Cy5$: single FRET step systems were modified from the 48mer structures detailed in Wang and Seeman[7]. The dendrimer sequences were designed *de novo*. All structures were visualized prior to ordering using NanoEngineer[3], which is a coarse grained modeling program. While not providing energy minimized structures, the visualization does allow for the identification of problematic junctions, so that they can be minimized by adjustment of segment length before a set of sequences is completed. The DNA sequences were designed such that the inter-dye spacing's were proportional to the desired Förster spacing, *e.g.* 0.5×, 1.0× and 1.5×R_0 for the $[Cy3\rightarrow Cy3.5\rightarrow Cy5]_n\rightarrow Cy5.5$ photonic wires series. The R_0 values used for each dye pair were from references[7, 10].

To design the DNA structures to assemble the dyes at the appropriate spacing's' the following formula was used[17]:

$$R_{DA} = \sqrt{(3.4*N+K)^2 + \left(L_D^2 + L_A^2 - 2L_D L_A \cos(\phi + 34.3*N)\right)} \qquad (14)$$

where the final spacing is R_{DA}, N is the number of separating bases, K is either 0 or 3.4 depending on whether the dye is on the same DNA helix or the opposite, and L_D and L_A are both 1.7, accounting for the 0.7 nm six carbon linker and 1 nm for half the width of the DNA helix. The 34.3 factor comes from the 360° of rotation divided by 10.5, the number of bases in a full turn. This accounts for the linker to which the dye is attached to the DNA, the width of the DNA molecule itself and the radial position around the DNA helix. In Supplementary Table 67 an array of representative calculations is shown.

The dyes were placed either on a 3' end (preferred), 5' end or an internal T* label (T* = amino C6-dT, see Figure S1). The $Cy3_n \rightarrow Cy5$ system implements an internal T labeling at the edge of the center junction for the Holliday junction and 8-arm star or at the center of the structure for the linear and bifurcated structures. The Cy3 dyes are all end-labeled. For the bifurcated, Holliday junction and 8-arm star the Cy3 is double labeled on the 3' and 5' ends. A variety of spacing's were investigated, including 0.75, 0.87, 1.0, 1.25 and $1.5\times R_0$. For the $[Cy3 \rightarrow Cy3.5 \rightarrow Cy5]_n \rightarrow Cy5.5$ systems, whenever possible the constructs were a template motif with the smaller dye labeled oligos assembled onto the long strand. The $0.5\times R_0$ system required the use of a staggered or concatenated DNA construction (no template) to afford the correct spacing for oligo assembly. For the Holliday junction and 8-arm star structures, the center templates had to be labeled with internal Cy5 in addition to the internal Cy5.5 label to account for the close spacing. The dendrimer structures relied on internal spacing for all dyes except the Cy3. Double labeling of the dyes was used in most cases to allow the needed dye numbers to be assembled.

The sequences were designed in such a way to maximize the base overlap so that there was a minimum of 9 bases before a nick or junction occurs. To further afford stability each portion of the structure was tested for melting temperature using Oligo Analyzer (https://www.idtdna.com/pages/scitools). A minimum melt temperature of 30°C at a salt concentration corresponding to 2.5X PBS buffer was required. The oligos were also checked for self-complementarity and cross-complementarity with non-desired sequences uses Operon's Oligo Analysis Tool (www.operon.com/tools/oligo-analysis-tool.aspx). Here a maximum of 5 bases of non-specific complementarity was set as the limit.

The $[Cy3 \rightarrow Cy3.5 \rightarrow Cy5]_n \rightarrow Cy5.5$ system was designed to assemble the dyes similar to the 2-way system, albeit in a 4 dye $D1_n$-$D2_n$-$D3_n$-A cascade, where n again equals 1, 2, 4, or 8. Given the added complexity, these structures are only measure at three different Forster distances, 0.5, 1.0 and $1.5\times R_0$. The 1.0 and $1.5\times R_0$ spacing structures are designed similar to the 2-dye system. To account for the additional space needed for the cascading FRET dyes, one side of the double stranded arm is extended. This occurs on the linear and bifurcated structures and on each arm of the Holliday and 8-arm star. The extended arms then act as a template for the smaller dye labeled oligos to be assembled onto. Given the close distances needed in the $0.5\times R_0$

spacing, the assembly proceeds differently. For the linear structure, 4 strands concatenate together to form the structure. Each of these strands contains one dye. The bifurcated structure also uses concatenated strands but requires the Cy5 to be double labeled and one of the oligos to be double labeled with Cy3 on the 3' end and Cy5.5 on the 5' end. All other oligos contain one dye either end- or internally -labeled as indicated in within individual sequences. The Holliday and 8-arm structures contain an internal Cy5.5 at the center junction. Two of the remaining central oligos, for the 4-way and Holliday and 8-arm structures contain double internally labeled Cy5 dyes. The Cy3 and Cy3.5 are assembled similar to the linear structures in a concatenated manner at the end of the central oligos.

The last set of dendrimeric DNA structures, $[[Cy3_n \rightarrow Cy3.5]_n \rightarrow Cy5]_n \rightarrow Cy5.5$, aimed to create a system where each acceptor has multiple donors according to the following: $D1_n^3$ – $D2_n^2$ – $D3_n$ – A, where n equal 2, 3, or 4. In order to facilitate this type of structure an exponential branching motif is used. The dendrimer system in the center begins with a branch of n arms and then each subsequent branch contains n+1 arms. Given the complexity of these structures only the $0.5 \times R_0$ spacing was designed and assembled. The structures are designed with 2 long 58 base oligos, one of which is internally labeled at the center with Cy5.5 and the other has 2 internal Cy5 labels that occur at the branching junction. 40 base oligos are assembled to the 58 base center and are double internally labeled with Cy3.5. Finally, the ends are 18 base oligos with 3' and 5' labeled Cy3. Some predictions of DNA structure end-to-end length and base separation calculations can be found in Supplementary Tables 65-67.

Spectral Decomposition

For each construct, the basic data set consisted of its PL spectrum plus direct excitation spectra for molar equivalents of each of its constituent fluorophores (assembled on the DNA) and of the background. The first step in analysis of such data was to decompose the full PL spectrum into its individual fluorophore contributions, a task that was generally straightforward given their spectral separation (Supplementary Figure 53). The regression procedure used was much like that in our previous work [7, 10], including the use of the Multipeak Fitting Tool in Igor Pro (v. 6.31)[18], and was carried out starting with the primary donor and removing each successive contribution before moving on to the next dye. The quality of the fits, as judged by plots of the

residuals, was generally quite good. Finally, for many purposes what is desired is the total emission output from each fluorophore. This was obtained by numerically integrating the individual dye contributions with the output from dye *m* denoted by Φ_m; the analogous values from the control experiments on the individual dyes are denoted Φ_m^0. In terms of this decomposition, the PL spectra of the full structure ($G(\lambda)$) and of the controls ($G_m(\lambda)$) have the respective representations

$$G(\lambda) = \sum_{m=1}^{M} f_m(\lambda)\Phi_m \qquad G_m(\lambda) f_m(\lambda)\Phi_m^0 \tag{15}$$

where $f_m(\lambda)$is the normalized emission spectrum of dye *m*.

Instrumental fluorescence response *versus* dye concentration.

In order to verify that the concentrations/volumes of dyes we were using did not suffer from inner filtering effects, we tested our Tecan Fluorometer for a linear response for excitation of each dye over a concentration range that spanned from 2 orders of magnitude above and below the ~1 μM working concentrations to be found within our structures. All responses using our typical range of instrumental settings and sample volumes were within a linear regime. Supplementary Figure 52 shows representative data from Cy3 and Cy3.5 fluorescence (excitation at 515 nm) collected from 1.0×R_0 linear control structures.

Experimental Setup for spFRET analysis of 0.75×R_0 $Cy3_n$→Cy5 structures

Single-pair FRET (spFRET) experiments[19] were carried out using an Axiovert inverted microscope (Zeiss). Laser excitation was obtained using the 515 nm line of an argon ion laser that was coupled into a single mode optical fiber. The output from the fiber was then tightly focused into the sample solution using a 100X Neofluar objective (1.4 N.A., Zeiss). The DNA structures were placed in individual sample wells of an eight-well sample tray (Thermo-Scientific) at a concentration 30-50 pM in 2.5 X PBS. The freely diffusing single DNA structures were excited as they passed through the laser focus. To reduce photobleaching of the dyes an oxygen scavenging system was used consisting of 4 mg/ml of glucose, 2 mM trolox (Sigma Aldrich), 1 mg/ml glucose oxidase (Sigma Aldrich), and 0.04 mg/ml catalase (Sigma Aldrich)[20]. To inhibit DNA melting the solutions were kept near 5°C using a water-cooled metal jacket

placed around the sample tray. The fluorescence from the samples was focused onto a 75 micron pinhole to reject the out-of-focus emission. After the pinhole, the Cy3 and Cy5 fluorescence was separated using a dichroic filter (FF640-Di01, Semrock) and then detected using a single photon counting avalanche photodiode detector module (SPCM-ARQH-14, Excelitas) in each channel. The fluorescence burst signal from each detector was collected using a counter/timer board (PCI-6602, National Instruments). The laser power was adjusted to give a maximum burst level of about 100 counts. For structures with a single Cy3 donor, the optical power was typically less than 75 μW before the objective. For other structures, the laser power was adjusted as explained below. The signals from each channel were processed into FRET histograms using custom designed software.

Supplementary References

1. Lakowicz J. R. Principles of Fluorescence Spectroscopy. Springer (2006).

2. Clegg R. M., Murchie A. I. H., Zechel A., Lilley D. M. J. Observing the helical geometry of double-stranded DNA in solution by fluorescence resonance energy-transfer. *P. Natl. Acad. Sci. USA* **90**, 2994-2998 (1993).

3. Nanoengineer. This software tool, originally produced by Nanorex, Inc., remains available, e.g., http://nanoengineer-1.software.informer.com/.

4. Iqbal A., *et al.* Orientation dependence in fluorescent energy transfer between Cy3 and Cy5 terminally attached to double-stranded nuclelic acids. *P. Natl. Acad. Sci. USA* **105**, 11176-11181 (2008).

5. Norman D. G., Grainger R. J., Uhrin D., Lilley D. M. J. Location of cyanine-3 on double-stranded DNA: Importance for fluorescence resonance energy transfer studies. *Biochemistry-Us* **39**, 6317-6324 (2000).

6. Sanborn M. E., Connolly B. K., Gurunathan K., Levitus M. Fluorescence properties and photophysics of the sulfoindocyanine Cy3 linked covalently to DNA. *J. Phys. Chem. B* **111**, 11064-11074 (2007).

7. Spillmann C. M., *et al.* Achieving effective terminal exciton delivery in quantum dot antenna-sensitized multistep DNA photonic wires. *ACS Nano* **7**, 7101-7118 (2013).

8. Wang X., Seeman N. C. Assembly and characterization of 8-arm and 12-arm DNA branched junctions. *J. Am. Chem. Soc.* **129**, 8169-8176 (2007).

9. Dutta P. K., Varghese R., Nangreave J., Lin S., Yan H., Liu Y. DNA-directed artificial light-harvesting antenna. *J. Am. Chem. Soc.* **133**, 11985-11993 (2011).

10. Boeneman K., *et al.* Self-assembled quantum dot-sensitized multivalent DNA photonic wires. *J. Am. Chem. Soc.* **132**, 18177-18190 (2010).

11. Hannestad J. K., Sandin P., Albinsson B. Self-assembled DNA photonic wire for long-range energy transfer. *J. Am. Chem. Soc.* **130**, 15889-15895 (2008).

12. Vogel S. S., van der Meer B. W., Blank P. S. Estimating the distance separating fluorescent protein FRET pairs. *Methods* **66**, 131-138 (2014).

13. Stryer L. Fluorescence energy-transfer as a spectroscopic ruler. *Annu. Rev. Biochem.* **47**, 819-846 (1978).

14. Kalinin S., *et al.* A toolkit and benchmark study for FRET-restrained high-precision structural modeling. *Nat. Methods* **9**, 1218-U1129 (2012).

15. Dolghih E., Roitberg A. E., Krause J. L. Fluorescence resonance energy transfer in dye-labeled DNA. *J. Photoch. Photobio. A* **190**, 321-327 (2007).

16. Gruber H. J., *et al.* Anomalous fluorescence enhancement of Cy3 and Cy3.5 versus anomalous fluorescence loss of Cy5 and Cy7 upon covalent linking to IgG and noncovalent binding to avidin. *Bioconjugate Chem.* **11**, 696-704 (2000).

17. Dietrich A., Buschmann V., Muller C., Sauer M. Fluorescence resonance energy transfer (FRET) and competing processes in donor–acceptor substituted DNA strands: a comparative study of ensemble and single-molecule data. *Rev. Mol. Biotechnol.* **82**, 211-231 (2002).

18. Medintz I. L., *et al.* Multiplex charge-transfer interactions between quantum dots and peptide-bridged ruthenium complexes. *Anal. Chem.* **81**, 4831-4839 (2009).

19. Dahan M., Deniz A. A., Ha T. J., Chemla D. S., Schultz P. G., Weiss S. Ratiometric measurement and identification of single diffusing molecules. *Chem. Phys.* **247**, 85-106 (1999).

20. Laurence T. A., Kong X. X., Jager M., Weiss S. Probing structural heterogeneities and fluctuations of nucleic acids and denatured proteins. *P. Natl. Acad. Sci. USA* **102**, 17348-17353 (2005).
